# Unraveling cradle-to-grave disease trajectories from multilayer comorbidity networks

**DOI:** 10.1038/s41746-024-01015-w

**Published:** 2024-03-07

**Authors:** Elma Dervić, Johannes Sorger, Liuhuaying Yang, Michael Leutner, Alexander Kautzky, Stefan Thurner, Alexandra Kautzky-Willer, Peter Klimek

**Affiliations:** 1 Complexity Science Hub Vienna, Vienna, Austria; 2 Supply Chain Intelligence Institute Austria (ASCII), Vienna, Austria; 3 Medical University of Vienna, Section for Science of Complex Systems, CeMSIIS, Vienna, Austria; 4 Medical University of Vienna, Department of Internal Medicine III, Clinical Division of Endocrinology and Metabolism, Vienna, Austria; 5 Medical University of Vienna, Department of Psychiatry and Psychotherapy, Vienna, Austria; 6 Santa Fe Institute, Santa Fe, NM USA; 7 Gender Institute, Gars am Kamp, Austria

**Keywords:** Epidemiology, Diseases

## Abstract

We aim to comprehensively identify typical life-spanning trajectories and critical events that impact patients’ hospital utilization and mortality. We use a unique dataset containing 44 million records of almost all inpatient stays from 2003 to 2014 in Austria to investigate disease trajectories. We develop a new, multilayer disease network approach to quantitatively analyze how cooccurrences of two or more diagnoses form and evolve over the life course of patients. Nodes represent diagnoses in age groups of ten years; each age group makes up a layer of the comorbidity multilayer network. Inter-layer links encode a significant correlation between diagnoses (*p* < 0.001, relative risk > 1.5), while intra-layers links encode correlations between diagnoses across different age groups. We use an unsupervised clustering algorithm for detecting typical disease trajectories as overlapping clusters in the multilayer comorbidity network. We identify critical events in a patient’s career as points where initially overlapping trajectories start to diverge towards different states. We identified 1260 distinct disease trajectories (618 for females, 642 for males) that on average contain 9 (IQR 2–6) different diagnoses that cover over up to 70 years (mean 23 years). We found 70 pairs of diverging trajectories that share some diagnoses at younger ages but develop into markedly different groups of diagnoses at older ages. The disease trajectory framework can help us to identify critical events as specific combinations of risk factors that put patients at high risk for different diagnoses decades later. Our findings enable a data-driven integration of personalized life-course perspectives into clinical decision-making.

## Introduction

Multimorbidity, the occurrence of two or more diseases in one patient, is a frequent phenomenon ^1,2^. Today’s reality of a 100-year lifespan brings a shifting multimorbidity burden and increased healthcare and long-term care costs ^3,4^. It was estimated that more than 50 million people in Europe show more than one chronic condition ^5^. In ^6^, authors estimated that 16–57% of adults in developed countries are diagnosed with more than one chronic disease and predicted a dramatic rise of multimorbidity rates in the next years. The WHO World Report on Ageing and Health emphasizes the importance of research to better understand the dynamics and consequences of aging ^7^. Studies on multimorbidity patterns may contribute to successful aging by the prevention of disease progression by identifying critical events that lead to a rapid deterioration of health ^8,9^.

As diseases tend to co-occur and interact with each other (in a way that can worsen the course of both), they cannot be studied separately from each other ^10^. The analysis of multimorbidity has recently been catalyzed by the massive collection of patient health information on diagnoses, medication, and results of laboratory tests in electronic health records (EHR), and other clinical registries. Comorbidity networks have been established as tools to analyze multimorbidity in such datasets ^11,12^. Age and sex-specific analyses can further be conducted to address age- and sex-dependent associations between diagnoses ^13,14^. These works confirm that patients mainly develop diseases in close network proximity to disorders they already suffer.

The concept of disease trajectories has been proposed to formally describe the progression of multimorbidity over time. Disease trajectories are frequently occurring patterns or sequences of diagnoses at particular times and are typically extracted from the medical history of millions of patients. Thus, apart from the pairwise disease associations, uncovering complex disease patterns and assessing their temporal directionality is crucial for estimating disease progression, developing prediction models ^15,16^, analyzing trajectories ^17,18^ and their temporal patterns using clustering algorithms ^19,20^. Many studies used data on in-hospital stays to construct such trajectories. A summary of applications of machine learning tools to understand the structure and formation of multimorbidity in the population was given in ^21^. Topological data analysis, offering a robust methodology to understand the high-dimensional structures, is also employed in the examination of disease trajectories ^22–25^. However, studies of multimorbidity patterns over the full life span of patients, from cradle to grave, remain scarce, as studies frequently take cross-sectional approaches ^2,26^.

Longitudinal analysis of multimorbidity requires large population-wide disease registries which span over multiple years, if not decades. Such analyses are challenging as they require custom-made methods and that are often computationally challenging ^17^. Taken together, a life span perspective on multimorbidities addressing the need for more comprehensive knowledge on disease trajectories and their critical events is largely missing to date ^27^.

Here, we propose a novel approach to dynamic comorbidity networks from longitudinal population-wide healthcare data to comprehensively identify disease trajectories in an entire population. A multilayer comorbidity network is constructed where nodes correspond to diagnoses, layers to age groups, intralayer links to disease co-occurrences, and interlayer links encode the directionality of disease pairs (which diagnosis tends to occur first). We identify temporal disease trajectories as communities in this multilayer network. In some cases, these tightly connected communities share some nodes and be referred to as overlapping communities.

The central assumption of our approach is that communities of nodes in the comorbidity network represent patients’ disease trajectories. We identify overlapping communities rather than exclusive clusters as the same diseases (nodes) can naturally be part of different disease trajectories, i.e. sleep disorders in patients with and without obesity and diabetes mellitus type 2. We further try to identify critical events as points along trajectories, where two initially identical trajectories start to diverge and will lead to different outcomes in terms of disease burden (hospital utilization) and mortality.

**Fig. 1:**
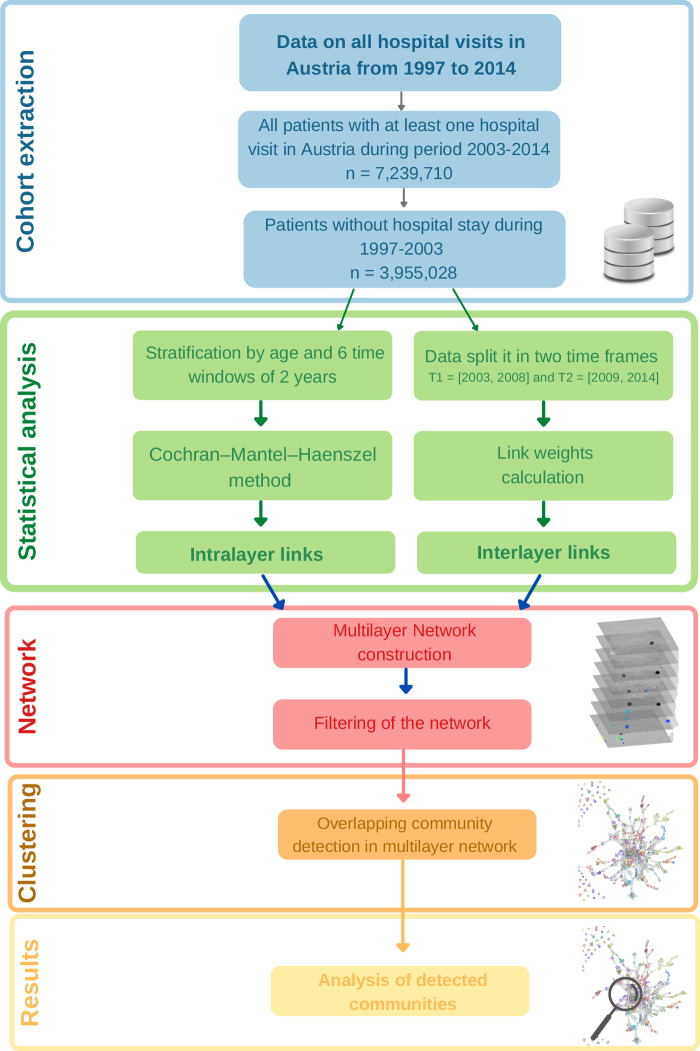
Workflow of the research presented in this article.

Figure 1 illustrates the suggested methodology of this large-scale disease trajectory study. We analyzed data from an electronic health registry covering almost all of 8.9 million Austrians with more than 44 million in-hospital stays over 17 years, from 1997-2014. To ensure the comparability of the health status of our study population, we restricted the analysis to patients who were “healthy" at the beginning of the observed period between 2003 and 2014. Therefore, in the first step of the analysis, we identified as the study population all patients with at least one hospital stay between 1997 and 2002 with a diagnosis from the range A00-N99 (in total 1081 diagnoses). Moreover, in the early 2000s, Austria transitioned from the previous ICD coding system to ICD-10 2001. It was crucial to avoid combining various classification systems as it would have compromised the reliability of the analysis, Fig. 1 (blue box).

In a next step we then constructed a multilayer comorbidity network to explore how different disease conditions co-occur and develop over time. We separated our data into 10-year age groups. For every age group we introduced a layer in the multilayer comorbidity network. In this network, two types of link can be found, links that connect nodes in the same layer (intralayer links) and links that connect nodes from different layers (interlayer links). All identified significant correlations of diagnoses in the same age group are defined as intralayer links, while interlayer links represent the correlation between diagnoses in different age groups, Fig. 1 (green box). Nodes without any intralayer links were removed, Fig. 1 (red box).

We used an algorithm based on the local optimization of a fitness function presented in ^28^ to identify overlapping communities in the multilayer network, Fig. 1 (orange box). One of the primary criteria for selecting this community detection algorithm was its ability to operate in an unsupervised manner, without the need to prestate the number of expected communities. Additionally, computational costs were an issue, given the size and density of our input network. Some community detection algorithms increase their complexity with the number of links in the network ^29^, rendering them highly unpractical for our rather dense networks. Algorithms like Bigclam, Demon, and COPRA work by spreading a signal through the network, which can be computationally expensive for networks with a large number of links. Note that in our framework the detected communities typically encompass more than one age layer. We analyzed the age structure of the detected overlapping communities and the number of chapters of diagnoses inside the communities. More concretely, we conceptualize disease trajectories as groups of diagnoses that occur at different age groups (layers in the network) and that are more closely connected to other diagnoses in the same community compared to diagnoses outside of the community.

As disease trajectories can overlap, this enables us to comprehensively study relationships between disease trajectories across more than one age group. We defined pairs of trajectories as converging if they do not overlap (no shared diagnoses) in younger age groups while they have a nonzero overlap in older age groups. Additionally, diverging pairs of trajectories overlap at the beginning, in younger age groups, but have different pathways in older age groups.

From this we can identify critical events in patient careers. Critical events are defined as combinations of diagnoses in a specific age group, mainly chronic conditions, that signal that the disease trajectories are about to diverge towards paths that lead to different levels of mortality or lengths of hospital stays in the following age groups. Critical events can be thought of as bifurcation points of disease trajectories that can lead to trajectories associated with strongly varying outcomes. These events can support the identification of patients at risk for more severe multimorbidity trajectories and associated adverse outcomes in the next decade and thereby provide leverage points for targeted preventive actions.

## Results

### Multilayer comorbidity network

**Fig. 2:**
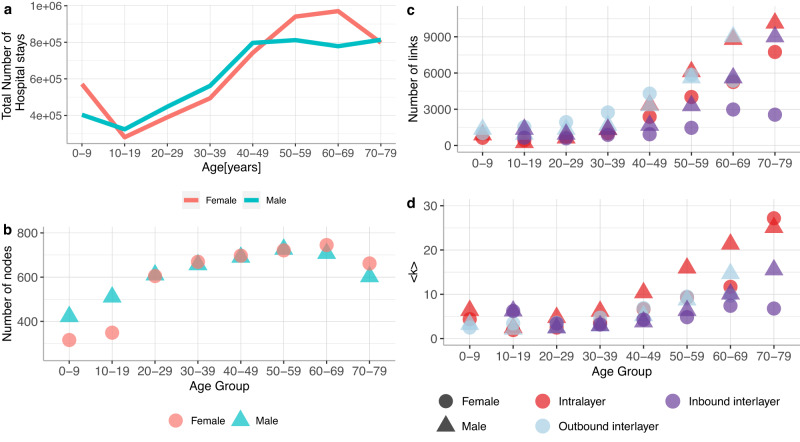
Summary statistics of the multilayer comorbidity network. Total number of hospital stays per age group, Network properties: number of nodes, number of all inter- and intralayer links and, the average degrees < *k* > .

We constructed the multilayer comorbidity network based on hospital data, basic characteristics of the database are shown in Figure S1. We used all 3-digits ICD10 codes from the range A00-N99 and one more newly introduced code for patients without any diagnosis, in total 1082 codes. Nodes in the constructed network are ICD10 codes appearing in one of eight different age groups, i.e. E66-0-9, E66-10-19, etc. Hence, we used 8,648 nodes to construct a multilayer comorbidity network with eight layers (one for each ten years age group, 0–9, 10–19,... 70–79 years old). We filtered the network by removing nodes without any intralayer links. This reduced the network from 8,648 nodes to 4,923 nodes for males and 4,764 nodes for females. The average degree in the filtered male network is 11.6 SD 39.7, for the female network the average degree is 15.8 SD 46. The number of hospital stays, Fig. 2a, and nodes *N* Fig. 2b increases with age, reaches a peak at ages 60 to 69, and decreases for older ages. We see similar age trends in Fig. 2c the total number of links and Fig. 2d the average degree for intralayer as well as in- or outbound interlayer links for males and females. Network properties are presented in Table 1. A comprehensive analysis of the network properties for each respective layer of the multilayer network is depicted in Supplementary, Figures 2 and 4.

**Table 1:** Network properties of the multilayer comorbidity network

Sex	Degree	Assortativity	Average Path Length	Betweenness	Closeness	Clustering Coefficient	Density	Modularity
Female	23.10	− 0.25	3.10	1117.43	0.00023	0.33	0.00024	0.75
Male	31.63	− 0.29	2.43	889.69	0.00014	0.32	0.00014	0.73

### Trajectories

**Fig. 3:**
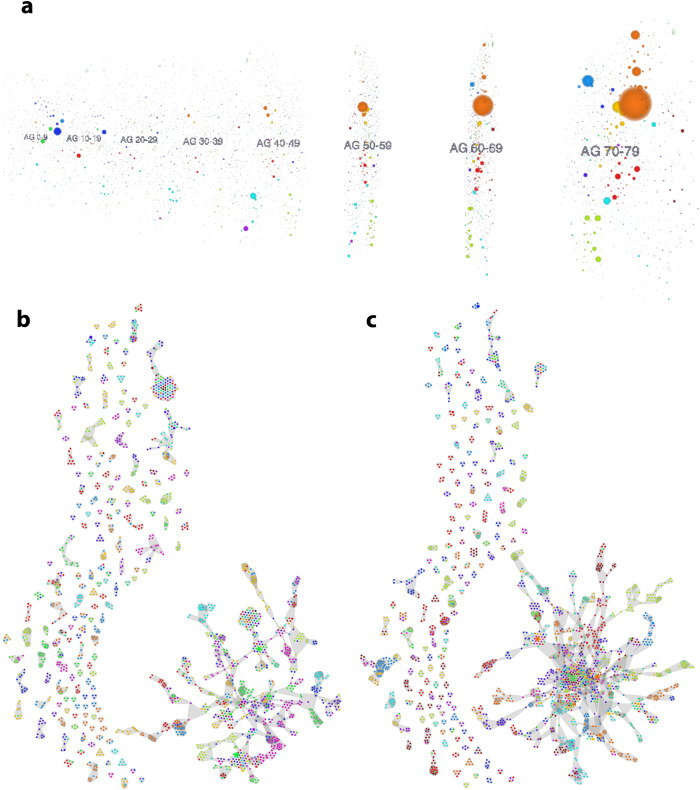
Visualization of disease trajectories. **a** Multilayer comorbidity network of female patients. Nodes represent diagnoses in age groups of ten years; each age group makes up a layer of the comorbidity multilayer network. The node color indicates the diagnose chapter; the diagnosis prevalence of the node in the network scales node size, Supplementary Fig. 3. All identified trajectories of female **b** and male **c** patients. The members of the trajectories are nodes of a multilayer comorbidity network (diagnose + 10 years age group). The Y-axis approximates the age groups of nodes; the node color indicates the diagnose chapter, and each gray area around nodes is one trajectory. More detailed visualization of these plots can be found in the interactive web application: **a** https://vis.csh.ac.at/netviewer/ and **b**, **c** https://vis.csh.ac.at/comorbidity_network_graphics/.

The unsupervised community detection algorithm discovered 642 distinct disease trajectories in the male and 618 in the female network. The remaining are listed in Supplementary Tables 5–6, and shown in Fig. 3. To evaluate the robustness of these findings and due to stochastic elements in the community detection algorithm, we performed the analysis three times with different random seeds (given the algorithm’s computational intensity) and observed identical results in each realization. These trajectories contain on average 9 (IQR 2–6) different diagnoses that range over up to 7 age groups (mean: 2.3 age groups), meaning that these trajectories range on average over 20–30 years and in some cases over up to 70 years of life. Besides trivial examples like a trajectory with the only diagnosis being K51 (ulcerative colitis) in each age group in males, we also found more complex trajectories spanning 70 years. For instance, for female patients, there is a trajectory that starts with personality disorder (F61) at the age of 20–29y. Over the following decades there is an accumulation of mental disorders including depression (F33), post-traumatic stress disorder (F43) and eating disorders (F50) in 50–59y, followed by anxiety disorders (F40) and a few more non-chronic diagnoses in 60–69y.

**Fig. 4:**
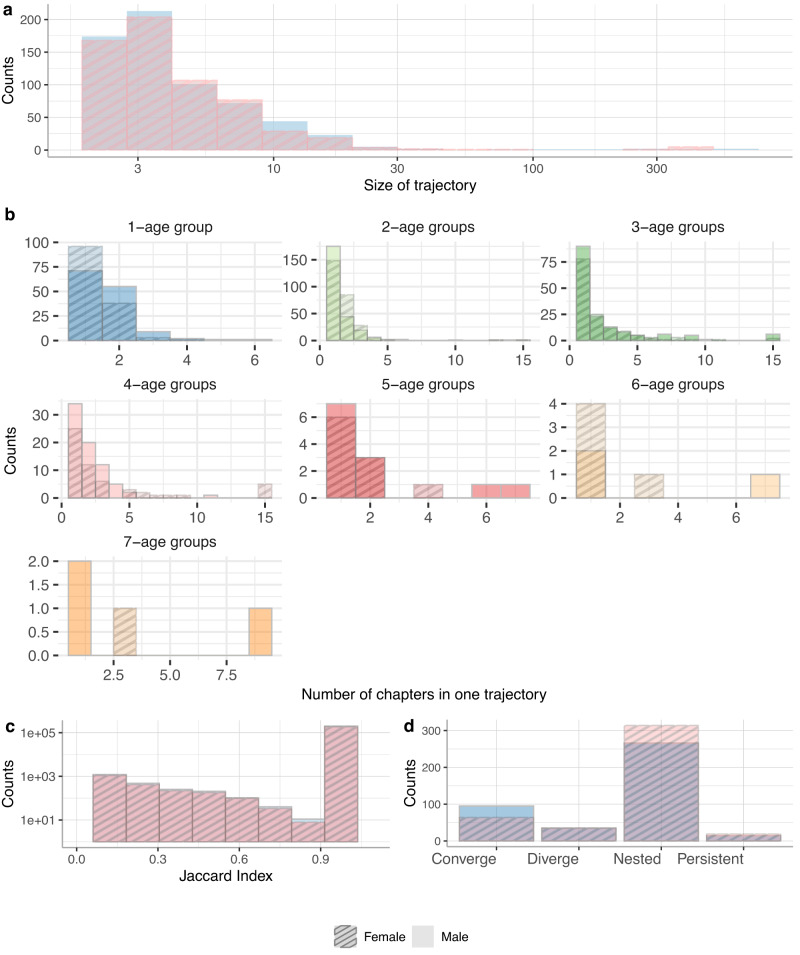
Properties of trajectories. We show for males and females **a** the distribution of the sizes of the trajectories, **b** the distributions of the number of different ICD chapters per each group of trajectories, i.e. trajectories which span over one age group - first blue plot, **c** the histogram of the pairwise Jaccard index and **d** frequency statistics of different types of trajectory pairs.

The distribution of the size of the trajectories (number of diagnoses-age tuples) is presented in Fig. 4a. Most trajectories contain between 3 and 5 diagnoses-age combinations; while a few trajectories contain more than a hundred elements. We split trajectories into seven groups based on the number of age groups in the trajectory and analyzed the number of different disease chapters in one trajectory Fig. 4b. This shows that trajectories typically span heterogeneous chapters of ICD codes, meaning that they often span diagnoses affecting quite different organ systems. We calculated the Jaccard index to inspect the pairwise similarity and dissimilarity of trajectories; see the distribution of this index in Fig. 4c. Jaccard indices range between zero and one, indicating varying degrees of similarity between two trajectories. The most common relationship among pairs of trajectories is nested, which explains the peak at one in the Jaccard index. Figure 4d shows frequency statistics of different types of trajectory pairs. We show a grid of scatterplots that comprehensively summarizes the relationships between size, number of ICD codes, number of ICD chapters, and number of age groups of trajectories in females and males in Supplementary Fig. 7 and online at https://vis.csh.ac.at/comorbidity_network_graphics/matrix_cluster/. As expected, there is a strong correlation between size, the number of ICD chapters, and specific ICD codes across both genders. Additionally, the size of the trajectories demonstrates a significant correlation with the number of incoming and outgoing links.

To validate the interpretations of trajectories within the context of established literature, we undertook a comprehensive literature survey utilizing the PubMed database. We quantified the associated publications for each disease pair within a given trajectory (spanning two or more ICD chapters). For each pair from the trajectories, we search the PubMed articles using the query ’(disease1) AND (disease2) and (correlation or association)’, as detailed in Supplementary Tables 8–9. From this analysis, we observed 2.2% of trajectories(ten trajectories) in males and 3.6% (16 trajectories) in females, wherein certain diseases lacked evident associations with other diseases in the same trajectory as per the PubMed records. For example, such trajectories consist of uncertain neoplasm of respiratory organs (D38) in the 60s and 70s and lung cancer (C34) in the 70s in females, and kidney cancer (C64) in 40s and 50s together with uncertain neoplasm of urinary organs (D41) in 40s and 40s in males. However, these trajectories are plausible, even though we could not find them in the literature, as they contain similar or the same diseases marked with different ICD10 codes.

**Fig. 5:**
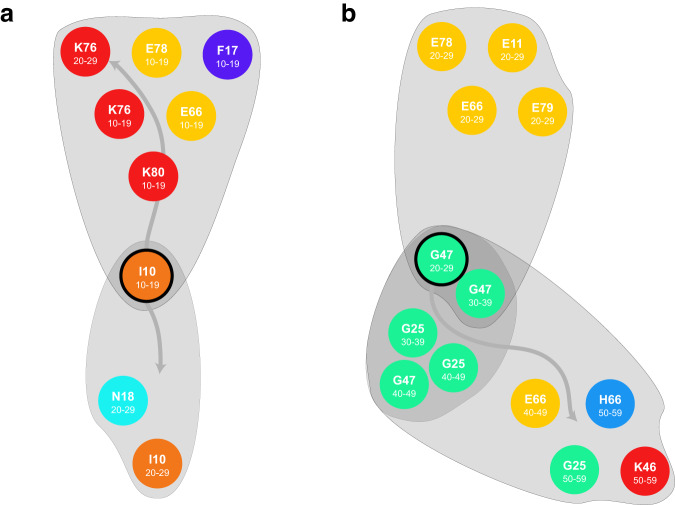
Two examples of diverging trajectories. **a** departing from hypertension (I10) at the age of 10–19y in females diverges to the “kidney-trajectory" and the “metabolic trajectory" **b** departing from sleep disorders (G47) at the age of 20–29y in males diverges to metabolic trajectory with diabetes mellitus type 2 (E11), obesity (E66), lipid disorders (E78) and hyperuricemia (E79) and path with movement disorders or otitis media (G25), obesity (E66) and abdominal hernia (K46).

In Fig. 5 we show a more detailed view of some of the trajectories from Fig. 3. We show two examples of trajectories (gray areas) departing from (A) hypertension (I10) at the age of 10–19y in females and (B) sleep disorders (G47) at an age of 20–29y in males. In both cases, different combinations of other diagnoses appear in subsequent decades. The hypertension trajectory diverged into chronic kidney diseases (2289 patients) or (1027 patients) a combination of metabolic (obesity, disorders of lipoprotein metabolism) and digestive disorders (liver diseases, cholelithiasis) with nicotine abuse. The sleep disorder trajectory diverged either toward the metabolic syndrome (including obesity and type 2 diabetes) in 115 patients or towards a combination of movement disorders, hernia, obesity, and diseases of the middle ear (316 patients).

In total, we identified 35 pairs of such diverging trajectories in females and 35 in males; see Fig. 4d). On average, diverging trajectories have 2.9 SD 0.8 age groups, 3.5 SD 1.8 different diagnoses chapters, and 8.1 SD 4.7 different diseases for females, and for males 3.0 SD 1 age groups, 3.5 SD 2.9 different diagnosis chapters, and 11 SD 11 different diseases. While there are 64 pairs of converging trajectories in females and 95 in males, converging trajectories in females have: 2.8 SD 0.9 age groups, 4.2 SD 3.2 different diagnoses chapters, and 26 SD 79 different diseases, in males: 3 SD 1 age groups, 3.8 SD 3.5 different diagnoses chapters, and 22 SD 68 different diseases. Some of the trajectories are persistent (16 pairs of trajectories in females, 14 in males).

The most frequent relationship between trajectories was the complete overlap of shorter and longer trajectories, which we defined as nested. We found 314 pairs of nested trajectories among female trajectories and 266 in male trajectories, Supplementary Tables 10–11.

We designed and implemented an online visualization tool that allows a user to interactively explore the comorbidity network structure and the underlying diagnose data, https://vis.csh.ac.at/netviewer/.

### Outcomes of trajectories

**Fig. 6:**
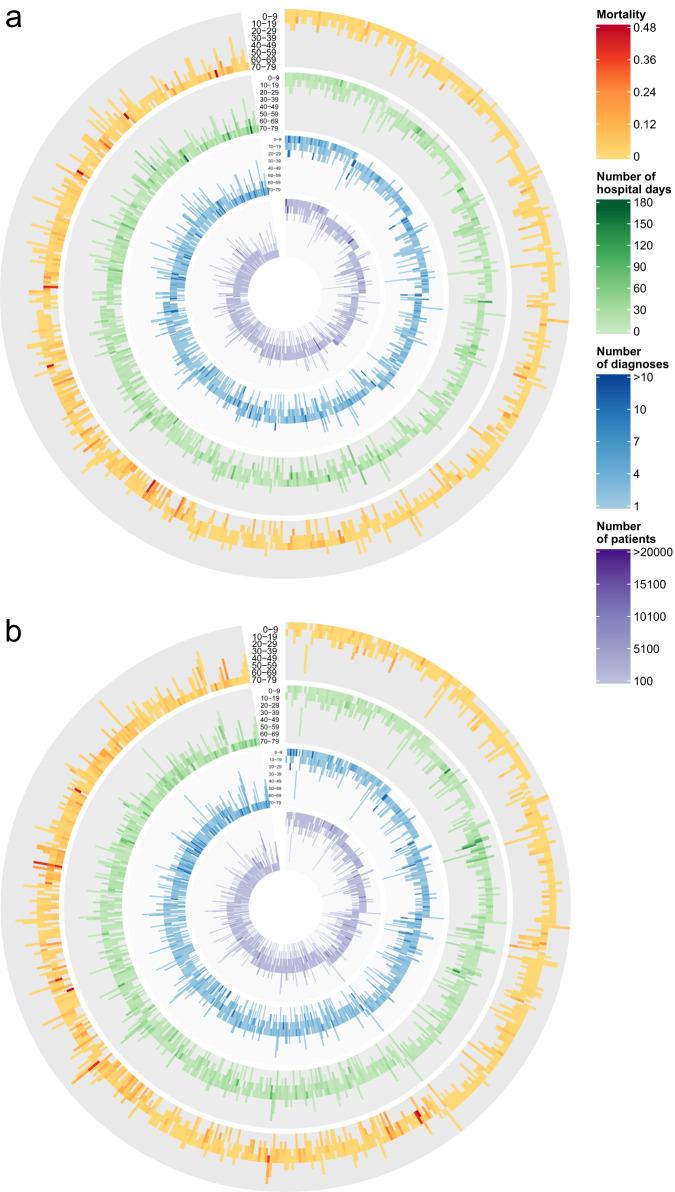
Outcomes of trajectories that span more than one age group. **a** Females **b** Males, the outer yellow circle shows mortality of each trajectory in each age group, the green circle presents an estimation of the average number of hospital days of patients of the trajectory, while the blue circle presents an estimation of the average number of diagnoses, the inner purple circle shows a number of patients who are following each trajectory in each age group. Each trajectory is represented by a single line within each circle, which is further divided based on the age groups the trajectory encompasses.

For every trajectory, we calculated (in-hospital) mortality and the number of days spent in the hospital for each age group, Fig. 6. In-hospital mortality for each trajectory is shown in the yellow outer circle. The analysis reveals notable variations in mortality rates across trajectories, with younger age groups generally exhibiting lower mortality. Moreover, it is evident that certain trajectories undergo significant shifts in mortality as they progress into older age groups. The green circle represents the average duration of hospitalization for trajectories, while the blue circle denotes the number of diagnoses, and the purple inner circle signifies the count of patients who followed at least 50% of a given trajectory. Notably, the green circle highlights discernible differences in the number of hospital days among different trajectories. Some trajectories have a clearly higher number of hospital days compared to other trajectories; these trajectories mainly consist of mental and behavioral disorders (F chapter) and infectious and parasitic diseases (B chapter) in males, while in females, besides these we see diseases of musculoskeletal and connective tissue (M chapter) and diseases of the nervous system (G chapter).

We also compared outcomes of diverging trajectories; some examples are shown in Table 2 (extended tables in Supplementary, Table 12–13). We calculated an average number of hospital diagnoses, hospital days, and hospital stays for each age group in each trajectory over all patients following these trajectories. We calculated the ratio of each outcome of trajectories in each diverging pair to check if these trajectories develop into different outcomes in terms of disease burden and mortality. For example, both trajectories from the pair starting with N81 in 50s are characterized with a similar average number of hospital diagnoses in the 20s, while in the 30s patients of the second trajectory have, on average, 24% more hospital diagnoses. In the same example, we see that patients of the first trajectory, on average have spent more days in hospital and have more hospital stays in the 20s (ratio of average number of days spent in hospital = 1.547/hospital stays = 1.548), but in 30s patients of the second trajectory, have spent more days in hospital and also more hospital stays (ratio of average number of days spent in hospital = 0.331/hospital stays = 0.551), Table 2.

**Table 2:** Outcomes of trajectories, four examples of diverging pairs of trajectories

Same diagnoses of trajectories, before they diverge	Exclusive diagnoses of the first trajectory	Exclusive diagnoses of the second trajectory	Age group	Ratio of average number (first vs. second trajectory) of number of		
				hospital diagnoses	days spent in hospital	hospital stays
Females						
I25-40-49	I21-40-49 I20-50-59	I42-50-59 I44-50-59	40–49	0.806	0.92	0.953
I42-40-49	I21-50-59 I24-50-59	I50-50-59 I51-50-59	50–59	0.685	0.522	0.713
I50-40-49		I42-60-69 I44-60-69				
		I44-70-79				
N81-50-59	N80-50-59 N84-50-59	N81-60-69 N99-60-69	50–59	1.052	0.862	1.013
	N85-50-59 N88-50-59		60–69	1.258	1.255	1.377
	N95-50-59 N80-60-69					
Males						
G47-20-29	E11-20-29 E66-20-29	G25-30-39 E66-40-49	20–29	1.021	1.547	1.548
G47-30-39	E78-20-29 E79-20-29	G25-40-49 G47-40-49	30–39	0.76	0.331	0.551
		G25-50-59 H66-50-59				
		K46-50-59				
G47-20-29	E11-20-29 E66-20-29	G25-30-39 G25-40-49	20–29	1.021	1.547	1.548
G47-30-39	E78-20-29 E79-20-29	G47-40-49	30–39	0.76	0.331	0.551

Columns same diagnoses of trajectories show initial overlap of trajectories, and exclusive diagnoses of the first and the second trajectory show to which states trajectories diverge. The ratio of the average number (first vs. second trajectory) of number of hospital diagnoses/ days spent in hospital/ hospital stays for each age group is presented for each age group.

## Discussion and conclusion

In this work, we introduced a novel method to identify life-course disease trajectories, in some cases spanning up to 70 years of life, in terms of sequences and combinations of hospital diagnoses that form and change over time. Our comprehensive analysis identified 642 disease trajectories in males and 618 in females ranging over the entire diagnostic spectrum (41% of males and 42% of female trajectories contained diagnoses from more than one ICD chapter). While the most common length of these trajectories was two diagnoses for both sexes, on average they contained 5.3 SD 5.1 and 5.4 SD 5.5 diagnoses for males and females, respectively, emphasizing the heterogeneous and widespread nature of multimorbidity in the general population.

There is a substantial variation in the number of patients that follow a trajectory. We count patients for each trajectory for each age group if they have at least 50% of diagnoses from a trajectory. In general, shorter trajectories tend to be followed by more patients (more than 10,000 patients per trajectory per age group) than longer, more specific ones that typically contain approximately a hundred patients. The number of patients in a trajectory typically increases with age.

The trajectories foster the rapid identification of critical events. These can take the form of bifurcation points where a trajectory “splits up” into multiple diverging trajectories at a specific age group. More concretely, we found 35 pairs of diverging trajectories for females and 35 pairs for males. For example, in females diagnosed with arterial hypertension (I10) between 10 and 19 years, two major trajectories were identified by the model. The first trajectory lead to the additional diagnosis of chronic kidney disease (N18) at an age of 20-29 years. This is clinically relevant as the number of pediatric arterial hypertension is increasing worldwide ^30^ and it is well known that aHTN is closely related to chronic kidney disease. There is a growing number of overweight and obesity during childhood worldwide ^31^. Obesity is closely related to the development of arterial hypertension, diabetes mellitus type 2 or dyslipidemia ^32^. The early onset of arterial hypertension for example is a major risk factor for the development of further diseases. In the present study we could show that some patients follow a trajectory that is related to an increased risk of developing chronic kidney disease, which increases mortality rate still in earlier ages ^33^. Therefore we can also refer to the following metabolic trajectory which is also a major health problem. However, there is lack of information in the specific trajectories which we show in the present study, pointing out that there are cohorts which develop chronic kidney disease under arterial hypertension and others who do not. Our results point out that there are some specific trajectories which should get more attention especially from clinical side of view - hence it should be identified whether the reason for the differences in the development of comorbidities are a result of differences in compliance, therapy or in health care. From a clinical point of view, a strict monitoring for arterial hypertension should be established especially in children at high risk, such as obese children or children with the metabolic syndrome. Arterial hypertension does not only mean increased risk for chronic kidney disease, but also other complications such as cardiovascular disease. The second trajectory was characterized by patients with the metabolic syndrome; these patients were disproportionally diagnosed with obesity (E66), lipid disorders (E78), steatosis hepatis (K76), cholelithiasis (K80) and nicotine abuse (N17) in their further life. In general, we therefore have two trajectories in females initially diagnosed with arterial hypertension, which are in principal dangerous conditions - the “kidney-trajectory” and the “metabolic trajectory”. We found that approximately 2289 patients follow the “kidney-” and 1027 patients follow the metabolic trajectory. These trajectories are mostly important as metabolic diseases belong to the most common diseases worldwide and also chronic kidney disease is a disease which is related to multi morbidity and increased mortality rate.

In a different example we found that sleeping disorders (G47) in males diagnosed in the age groups between 20-39 years were also followed by a metabolic trajectory which was defined by an over-representation of later diagnoses of diabetes mellitus type 2 (E11), obesity (E66), lipid disorders (E78) and hyperuricemia (E79). Among the sleeping disorders related to organic causes coded by G47, obstructive sleep apnea is a frequent comorbidity of obesity and other disorders related to the metabolic syndrome. The trajectory identified here suggests that a diagnosis of G47 typically precedes diagnosis of these metabolic disorders, calling for more timely identification of metabolic decompensation. The other trajectory, diverging from sleeping disorders, is characterized by a higher chance of being diagnosed with movement disorders (G25) or otitis media (H66), obesity (E66) and abdominal hernia (K46). While the link to obesity, a known risk for abdominal hernias, is shared between both trajectories, the association with movement disorders suggest a more neurologically impaired group of patients for whom organic sleeping disorders may be an early marker for risk of developing neurodegenerative disorders such as Parkinson disease. Obstructive sleep apnea was previously identified as common comorbidity and potential causal risk factor due to reduced brain oxygenation ^34^. While no conclusion on causality can be drawn, the trajetory identified here supports sleeping disorders as an early risk factor. We found substantial differences in the average number of diagnoses and hospital days between patients of different branches of these diverging trajectories. While patients who followed these two trajectories showed similar average numbers of diagnoses at age 20–29 (3.3 diagnoses in both cases), patients who followed a metabolic trajectory had, on average, 3.9 diagnoses ten years later while patients who followed the other trajectory had, on average, 5.1 diagnoses. The number of sleeping disorders is on the rise and these results show that patients with sleeping disorders have to be monitored for several diseases in different trajectories. Our analysis also identified several instances were diverging trajectories differed substantially in their mortality, in some cases of up to 18 times.

In terms of mortality we identify trajectories that develop into a combination of diagnoses with high mortality in older age groups. For instance, a trajectory consisting of chronic bronchitis and COPD at an age of 40–49y, bronchiectasis and intraoperative and postprocedural complications at 50–59y and finally in sequelae of tuberculosis, inflammatory polyneuropathy, conjunctivitis, bronchitis, bronchiectasis, eosinophilia and again intraoperative and postprocedural complications in 60–69y in males had eight times higher mortality in the age group 60–69y compared to its’ mortality ten years earlier (mortality increased from 0.089 in 40–49y to 0.013 before jumping to 0.11 in 60–69y). Trajectories with the highest mortality usually contain cancer diagnoses, but cardiovascular or respiratory diseases also feature in the trajectories with high mortality.

### Strengths and limitations

Strengths of this study include its comprehensive population-wide in-hospital database, containing information on about 9 million individuals. Non-systematic errors, such as randomly missing diagnoses, have little impact on our research because of the volume of the data set. However, this study has some limitations caused by data quality and limitations in data availability, in particular, the lack of information on outpatient visits, medication and lifestyle. Consequently, we cannot evaluate the outcomes of outpatient visits, blood tests, examinations, or imaging because primary care diagnoses are not recorded in this dataset; only hospital diagnoses coded with ICD10 codes were available for analysis. It further remains to be seen whether similar trajectories can be identified in populations of other countries than Austria or by means of different community detection algorithms.

Another drawback is that the database was designed for billing purposes, so diagnoses that did not result in financial compensation were frequently not reported. Therefore, we have to point out that some diseases, such as alcohol-related disorders or nicotine dependence, are often not recorded correctly in our data. Further, socio-economic indicators for individual patients were also not available in the dataset, leaving it yet to be explored how socio-economic status impacts on these trajectories. An additional constraint associated with the dataset is the exclusive availability of in-hospital mortality data. On a methodological level, it is also important to bear in mind that a constructed multilayer comorbidity network has two types of links (with normalized links weights); but these types are not distinguishable by the used community detection algorithms.

In summary, we presented a novel and statistically grounded way of studying disease progression over time based on a population-wide and decade-spanning data set of hospital diagnoses. We proposed an age multilayer comorbidity network as a base for our modeling approach. We showed that this kind of network is a promising approach for better understanding disease trajectories and their dynamics as patients age. While some of the identified trajectories in this study have been described in previously published studies, many novel disease trajectories and their decades-long time dynamics have been revealed.

A better understanding of diseases, their correlations and the sequences in they occur has the potential to improve the prevention of focal diseases. Early detection and identification of a patient’s projected disease trajectory might enable prompt and timely treatments next to targeted preventive action. Consequently, that will help transition health systems from single-disease models to more effective life-spanning and individualized multimorbidity models^35,36^. Our potential future research may focus on constructing trajectories derived from hypergraph-based comorbidity networks^37–39^.

## Data and methods

### Data

The analyzed dataset spans 17 years of nationwide in-hospital data from all hospitals in Austria. Each hospital stay is recorded with primary and secondary diagnoses, age in the resolution of 5 years, sex, admission and release date, release type (i.e., release, transfer, death...), Supplementary Fig. 1. This dataset covers the period from 1997 until 2014 and the vast majority of Austria’s population with 8.9 unique patients. Diagnoses are coded with the three-digit International Classification of Diseases, 10th Revision (ICD-10) codes. We restricted our analysis to 1081 codes from A00 to N99, excluding codes describing health encounters that can not be directly related to diseases (i.e., O00-O9A—Pregnancy, childbirth, and the puerperium, S00-T88 - Injury, poisoning and certain other consequences of external causes...). The data always reports a primary diagnosis as the main reason for hospitalization, along with a variable number of secondary diagnoses.

In this study, we assigned equal importance to both primary and secondary diagnoses^19,40^. To ensure that our study population’s health state was comparable at the beginning of the observation period and not in the middle of connected hospitalization episodes, we introduced a wash-out period and limited the analysis to patients who had no hospital visits between 1997 and 2002. Consequently, excluding these patients also ensured that analyzed data has only one ICD coding system, as in the early 2000s, Austria updated its ICD coding system to ICD-10 2001^19,40,41^.

### Ethics

We made secondary use of a research database containing medical claims records, which is securely managed by the Federal Ministry of Health. It is important to note that measures have been implemented to guarantee the anonymity of individuals within this database. It is a consolidated research database accessible only to authorized partners who adhere to stringent data protection policies. Our use of this data is conducted in collaboration with data provider and follows established agreements.

The data in this database do not include any personal identifiers, such as names, postal codes, or dates of birth. Additionally, all members of our research team have committed to maintaining confidentiality and complying with relevant data protection regulations through a signed agreement.

### Multilayer comorbidity network

Formally, we construct the multilayer comorbidity network given by the tensor ${M}_{i,j}^{\alpha ,\beta }$ where *i* and *j* refer to nodes (diagnoses) on layers (age groups) *α* and *β*, respectively. We refer to entries in *M* with *α* = *β* as intralayer links and with *α* ≠ *β* as interlayer links. The analysis was performed separately for male and female patients.

### Intralayer links

Intralayer links give the correlation between diagnoses within the same age group. The analyzed dataset was stratified by six time windows of two years each, from 2003 to 2014. A contingency table is created for each pair of diagnoses in each stratum (for each sex and age group, the intralayer analysis includes six strata, each covering two calendar years). We used all contingency tables with more than four patients in each subgroup to compute relative risks (RR) and the p-value for rejecting the null hypothesis that the co-occurrence of two analysed diagnoses is statistically independent ^41^. A weighted average of the estimates of the risk ratios and odds ratios across the stratified data were calculated by using the Cochran-Mantel-Haenszel method ^42^.

Subsequently, all correlations with RR higher than 1.5 and p-value smaller than 0.05 were extracted and presented as intralayer links ^14^. These links are bidirectional, and we use a normalized RR as the link weight. The normalization of RR was done such that the sum of all total weights of all intralayer links with the same target was one.

### Interlayer links

To estimate directionality or time order in pairs of diagnoses, we split the observation period in two time frames *T*1 = [2003, 2008] and *T*2 = [2009, 2014]. This choice of splitting the data ensures equally long observation periods for diagnoses in T1 and T2. We investigate if a patient diagnosed with *i* in *T*1 elevates the risk of being diagnosed with *j* in *T*2 and compute the interlayer link weight as

1 $${M}_{i,j}^{\alpha \ne \beta }=\frac{P({j}_{T2}^{\beta }| {i}_{T1}^{\alpha })}{P({j}_{T2}^{\beta })}.$$

### Overlapping community detection in multilayer network

We deleted all nodes without at least one inbound and one outbound link. Further, we normalized all link weights to range from 0 to 1 by dividing each link’s weight by the sum of all links of the same type of a target node

2 $${M}_{ij}^{\alpha \beta }=\frac{{M}_{ij}^{\alpha \beta }}{{\sum }_{j}{M}_{ij}^{\alpha \beta }}.$$

The algorithm for detecting the overlapping and hierarchical community structure in complex networks proposed in ^28^ was applied. This unsupervised clustering algorithm does not have a predefined number of communities. The detection procedure is initiated starting with a random node, which represents one community by itself.

A community’s fitness is defined as ${f}_{G}=\frac{{k}_{in}^{G}}{{({k}_{in}^{G}+{k}_{out}^{G})}^{a}}$,where ${k}_{in}^{G}$ are the total internal degrees of the nodes in the community *G* and ${k}_{out}^{G}$ are the total external degrees of the nodes in the community *G*.

As long as the *f*_*G*_ improves, neighboring nodes are added, or nodes that already are community members get removed in a step-wise manner.

The resolution parameter *a* enables us to uncover different hierarchical levels of a system, the natural choice is *a* = 1. Fitness is calculated at each step. Once the fitness cannot be increased anymore by a node removal or addition step, that community is “completed" and “closed." The community detection process ends when all nodes have been assigned to at least one community. To parallelize and optimize this computationally costly process, we identify the community of every node and delete duplicates among the discovered communities.

Identified communities usually consist of diseases in different age groups that tend to co-occur more frequently among themselves than diseases that are not part of the community. Hence, these communities represent typical disease trajectories; we denote a trajectory *X* as a set of diagnosis-age tuples, *X* = {(*i*_1_, *α*_1_), (*i*_2_, *α*_2_), (*i*_3_, *α*_3_). . . }, where *i* is an ICD10 code ranging from [*A*00, *N*99] and *α* is the age group from [1, 8].

We measure the similarity of trajectories by the Jaccard coefficient between two trajectories consisting of tuples with diagnoses *i* and age groups *α*, (*i*, *α*). That is, two trajectories have a non-zero overlap if they share diagnoses within the same age groups.

### Identifying converging and diverging trajectories

**Fig. 7:**
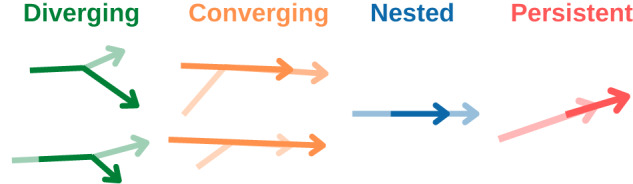
Visual representation of pairwise relations between pair of trajectories.

We performed a comprehensive classification with respect to all pairwise relations between every pair of trajectories. Provided that two trajectories share at least one diagnosis, they can be related in one of four different ways, namely (i) diverging, (ii) converging, (iii) nested, or (iv) persistent, Fig. 7.

Diverging trajectories have some overlapping elements at younger ages, but they develop into markedly different sets of diagnoses at older ages.

More formally, trajectories *X* = {(*i*_11_, *α*_11_), (*i*_12_, *α*_12_), (*i*_13_, *α*_13_). . . } and *Y* = {(*i*_21_, *α*_21_), (*i*_22_, *α*_22_), (*i*_23_, *α*_23_). . . } are diverging if it holds that

3 $$\begin{array}{r}\left\{\{({i}_{1i},{\alpha }_{1i})\in X| {\alpha }_{1i}={\alpha }_{\min }^{X}\left.\right)\}\cap \{({i}_{2i},{\alpha }_{2i})\in Y| {\alpha }_{2i}={\alpha }_{\min }^{X}\left.\right)\}\right\}\,\cup \\ \left\{\{({i}_{1i},{\alpha }_{1i})\in X| {\alpha }_{1i}={\alpha }_{\min }^{Y}\left.\right)\}\cap \{({i}_{2i},{\alpha }_{2i})\in Y| {\alpha }_{2i}={\alpha }_{\min }^{Y}\left.\right)\}\right\}\,\ne {{\emptyset}}\,{{{\rm{and}}}}\\ \{({i}_{1i},{\alpha }_{1i})\in X| {\alpha }_{1i} > {\alpha }_{\min }^{X}\left.\right)\}\,\ne \,\{({i}_{2i},{\alpha }_{2i})\in Y| {\alpha }_{2i} > {\alpha }_{\min }^{X}\left.\right)\}\,{{{\rm{and}}}}\\ \{({i}_{1i},{\alpha }_{1i})\in X| {\alpha }_{1i} > {\alpha }_{\min }^{Y}\left.\right)\}\,\ne \,\{({i}_{2i},{\alpha }_{2i})\in Y| {\alpha }_{2i} > {\alpha }_{\min }^{Y}\left.\right)\},\end{array}$$

where $${\alpha }_{\min }^{X}=\mathop{\min }\limits_{(i,\alpha )\in X}\,\alpha \,\,,\,{\alpha }_{\min }^{Y}=\mathop{\min }\limits_{(i,\alpha )\in Y}\,\alpha$$.

Converging trajectories overlap at older ages but are clearly different at younger ages. Trajectories *X* and *Y* are converging if it holds that

4 $$\begin{array}{r}\left\{\{({i}_{1i},{\alpha }_{1i})\in X| {\alpha }_{1i}={\alpha }_{\max }^{X}\left.\right)\}\cap \{({i}_{2i},{\alpha }_{2i})\in Y| {\alpha }_{2i}={\alpha }_{\max }^{X}\left.\right)\}\right\}\,\cup \\ \left\{\{({i}_{1i},{\alpha }_{1i})\in X| {\alpha }_{1i}={\alpha }_{\max }^{Y}\left.\right)\}\cap \{({i}_{2i},{\alpha }_{2i})\in Y| {\alpha }_{2i}={\alpha }_{\max }^{Y}\left.\right)\}\right\}\,\ne {{\emptyset}}\,{{{\rm{and}}}}\\ \{({i}_{1i},{\alpha }_{1i})\in X| {\alpha }_{1i} < {\alpha }_{\max }^{X}\left.\right)\}\,\ne \,\{({i}_{2i},{\alpha }_{2i})\in Y| {\alpha }_{2i} < {\alpha }_{\max }^{X}\left.\right)\}\,{{{\rm{and}}}}\\ \{({i}_{1i},{\alpha }_{1i})\in X| \left.{\alpha }_{1i} < {\alpha }_{\max }^{Y}\right)\}\,\ne \,\{({i}_{2i},{\alpha }_{2i})\in Y| \left.{\alpha }_{2i} < {\alpha }_{\max }^{Y}\right)\},\end{array}$$

where $${\alpha }_{\max }^{X}=\mathop{\max }\limits_{(i,\alpha )\in X}\,\alpha \,\,,\,{\alpha }_{\max }^{Y}=\mathop{\max }\limits_{(i,\alpha )\in Y}\,\alpha$$.

Two trajectories are nested if one of them is a subset of another one, *X* ⊂ *Y* or *Y* ⊂ *X*. Persistent trajectories *X* and *Y* can overlap in the highest age group of *X* and lowest age group of *Y*, or vice versa.

### Identifying critical events

We define critical events by one or a combination of diagnoses and age groups where two trajectories begin to diverge and where one of the diverging trajectories has patients with a considerably higher number of diagnoses, higher mortality or more extended hospital stays in the subsequent age group(s) compared to the other diverging trajectory. Mortality of a trajectory for a certain age group is calculated as *M* = ∑_*i*_*m*_i_*∏_*j*≠*i*_(1 − *m*_*j*_), where *m* is the in-hospital mortality of a diagnosis (defined as the percentage of patients diagnosed with the diagnose in a specific age group who die in-hospital) which is a member of a trajectory. The length of hospital stay of a trajectory in a certain age group is defined as the average number of days spent in hospital for patients who are diagnosed with at least half of all diagnoses from a trajectory.

### Supplementary information

SUPPLEMENTAL MATERIAL

## Data Availability

The raw and processed patient data are not available due to privacy laws. The dataset is safeguarded by the Austrian Federal Ministry of Health and made accessible to research institutions under strict data protection regulations. To gain access to this data, researchers have to find individual arrangements with the Austrian Federal Ministry of Health.

## Code availability

Custom code for the analysis is available and per request from the authors.

## References

[1] Han X (2021). Disease trajectories and mortality among individuals diagnosed with depression: a community-based cohort study in UK Biobank. Mol. Psychiatry.

[2] Cezard G, McHale C, Sullivan F, Bowles J, Keenan K (2021). Studying trajectories of multimorbidity: a systematic scoping review of longitudinal approaches and evidence. BMJ Open.

[3] World Health Organization - Ageing and health. Last accessed May 01, 2022 from https://www.who.int/news-room/fact-sheets/detail/ageing-and-health.

[4] Ageing Europe: Looking at the lives of older people in the eu, 2019 edition from https://ec.europa.eu/eurostat/.

[5] Struckmann V (2014). Caring for people with multiple chronic conditions in Europe. Eurohealth.

[6] Hajat C, Stein E (2018). The global burden of multiple chronic conditions: a narrative review. Prev. Med. Rep..

[7] Organization, W. World report on ageing and health. (World Health Organization,2015)

[8] Rowe J, Kahn R (1997). Successful aging. Gerontologist.

[9] Kudesia P (2021). The incidence of multimorbidity and patterns in accumulation of chronic conditions: a systematic review. J. Multimorb. Comorb..

[10] Di Angelantonio E (2015). Association of cardiometabolic multimorbidity with mortality. Jama.

[11] Strauss V, Jones P, Kadam U, Jordan K (2014). Distinct trajectories of multimorbidity in primary care were identified using latent class growth analysis. J. Clin. Epidemiol..

[12] Fotouhi B, Momeni N, Riolo M, Buckeridge D (2018). Statistical methods for constructing disease comorbidity networks from longitudinal inpatient data. Appl. Netw. Sci..

[13] Jeong E, Ko K, Oh S, Han H (2017). Network-based analysis of diagnosis progression patterns using claims data. Sci. Rep..

[14] Chmiel A, Klimek P, Thurner S (2014). Spreading of diseases through comorbidity networks across life and gender. N. J. Phys..

[15] Violá C (2020). Five-year trajectories of multimorbidity patterns in an elderly Mediterranean population using Hidden Markov Models. Sci. Rep..

[16] Prados-Torres A (2018). Cohort profile: the epidemiology of chronic diseases and multimorbidity. The EpiChron cohort study. Int. J. Epidemiol..

[17] Siggaard T (2020). Disease trajectory browser for exploring temporal, population-wide disease progression patterns in 7.2 million Danish patients. Nat. Commun..

[18] Jensen A (2014). Temporal disease trajectories condensed from population-wide registry data covering 6.2 million patients. Nat. Commun..

[19] Haug N (2020). High-risk multimorbidity patterns on the road to cardiovascular mortality. BMC Med..

[20] Giannoula A, Gutierrez-Sacristá n A, Bravo Á, Sanz F, Furlong L (2018). Identifying temporal patterns in patient disease trajectories using dynamic time warping: a population-based study. Sci. Rep..

[21] Hassaine A, Salimi-Khorshidi G, Canoy D, Rahimi K (2020). Untangling the complexity of multimorbidity with machine learning. Mech. Ageing Dev..

[22] Chazal F, Michel B (2021). An introduction to topological data analysis: fundamental and practical aspects for data scientists. Front. Artif. Intell..

[23] Dagliati A (2020). Using topological data analysis and pseudo time series to infer temporal phenotypes from electronic health records. Artif. Intellig. Med..

[24] Tucker A, Garway-Heath D (2009). The pseudotemporal bootstrap for predicting glaucoma from cross-sectional visual field data. IEEE Trans. Inf. Technol. Biomed..

[25] Campbell K, Yau C (2018). Uncovering pseudotemporal trajectories with covariates from single cell and bulk expression data. Nat. Commun..

[26] Hsu H (2015). Trajectories of multimorbidity and impacts on successful aging. Exp. Gerontol..

[27] Vos R, Akker M, Boesten J, Robertson C, Metsemakers J (2015). Trajectories of multimorbidity: exploring patterns of multimorbidity in patients with more than ten chronic health problems in life course. BMC Family Pract..

[28] Lancichinetti A, Fortunato S, Kerté sz J (2009). Detecting the overlapping and hierarchical community structure in complex networks. N. J. Phys..

[29] Javed M, Younis M, Latif S, Qadir J, Baig A (2018). Community detection in networks: a multidisciplinary review. J. Netw. Comput. Appl..

[30] Ashraf M, Irshad M, Parry N (2020). Pediatric hypertension: an updated review. Clin. Hypertension.

[31] World Health Organization, https://www.who.int/news-room/fact-sheets/detail/obesity-and-overweight#::~text=The.

[32] Juonala M (2011). Childhood adiposity, adult adiposity, and cardiovascular risk factors. N. Engl. J. Med..

[33] Bikbov B (2020). Global, regional, and national burden of chronic kidney disease, 1990-2017: a systematic analysis for the Global Burden of Disease Study 2017. Lancet.

[34] Schulte E, Winkelmann J (2011). When Parkinson’s disease patients go to sleep: specific sleep disturbances related to Parkinson’s disease. J. Neurol..

[35] Zou S, Wang Z, Bhura M, Tang K (2022). Association of multimorbidity of non-communicable diseases with mortality: a 10-year prospective study of 0.5 million Chinese adults. Public Health.

[36] Fortunato S, Hric D (2016). Community detection in networks: a user guide. Phys. Rep..

[37] Sun, Z. et al. EHR2HG: Modeling of EHRs Data Based on Hypergraphs for Disease Prediction. *2022 IEEE International Conference On Bioinformatics And Biomedicine (BIBM)*. pp. 1730–1733 (2022).

[38] Billings, J. et al. Simplex2vec embeddings for community detection in simplicial complexes. *ArXiv Preprint ArXiv:1906.09068*. (2019).

[39] Patania A, Vaccarino F, Petri G (2017). Topological analysis of data. EPJ Data Sci..

[40] Deischinger C (2020). Diabetes mellitus is associated with a higher risk for major depressive disorder in women than in men. BMJ Open Diab. Res. Care.

[41] Dervic E (2021). The effect of cardiovascular comorbidities on women compared to men: longitudinal retrospective analysis. JMIR Cardio.

[42] Kuritz S, Landis J, Koch G (1988). A general overview of Mantel-Haenszel methods: applications and recent developments. Ann. Rev. Public Health.

