# Supplementary material for: Unraveling cradle-to-grave disease trajectories from multilayer comorbidity networks
Source: NPJ Digit Med. 2024 Mar 7;7:56. doi: 10.1038/s41746-024-01015-w (PMC10920888; doi:10.1038/s41746-024-01015-w)
Supplement: Supplementary file 1 — SUPPLEMENTAL MATERIAL [file 41746_2024_1015_MOESM1_ESM.pdf]

Unravelling cradle-to-grave disease trajectories from multilayer comorbidity networks

Elma Dervić [1,2,3] , Johannes Sorger[1] , Liuhuaying Yang[1] , Michael Leutner[4] , Alexander Kautzky[5] , Stefan Thurner [1,3,6] , Alexandra Kautzky-Willer[4,7] , Peter Klimek[2,3,1,*]

[1]Complexity Science Hub Vienna, Josefstadter Straße 39, 1080 Vienna, Austria;
[2]Supply Chain Intelligence Institute Austria (ASCII), Josefstadter Straße 39, 1080 Vienna, Austria;
[3]Medical University of Vienna, Section for Science of Complex Systems, CeMSIIS, Spitalgasse 23, 1090 Vienna, Austria;
[4]Medical University of Vienna, Department of Internal Medicine III, Clinical Division of Endocrinology and Metabolism, Wahringer Gurtel 18–20, A-1090 Vienna, Austria;
[5]Medical University of Vienna, Department of Psychiatry and Psychotherapy, Wahringer Gurtel 18-20, A-1090 Vienna, Austria;
[6]Santa Fe Institute, 1399 Hyde Park Road, Santa Fe, NM 87501, USA. 7Gender Institute, A-3571 Gars am Kamp, Austria

Suplementary Information

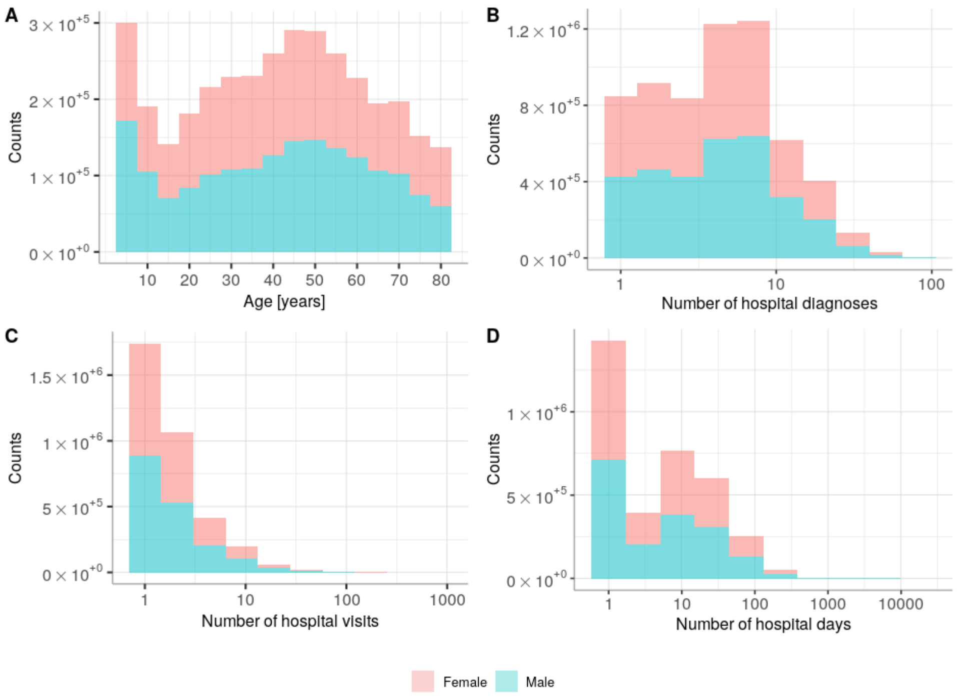

Supplementary Figure 1: Distribution of (A) age of patients, (B) average number of hospital diagnoses of patients, (C) average number of hospital visits of patients, (D) average number of hospital days of patients

Figure 2 shows an overview of interlayer links aggregated on the level of a chapter (of ICD10 codes) for each layer (age group) for female patients. The size of nodes is proportional to the prevalence of diagnoses from the chapter. Link weight between chapters A and B is the number of links oriented from chapter A- to B divided by the total number of outgoing links from chapter A. More detailed visualization of this plot can be found in the interactive WEB application: https://vis.csh.ac.at/comorbidity_network_graphics/mlcn_chapters.html

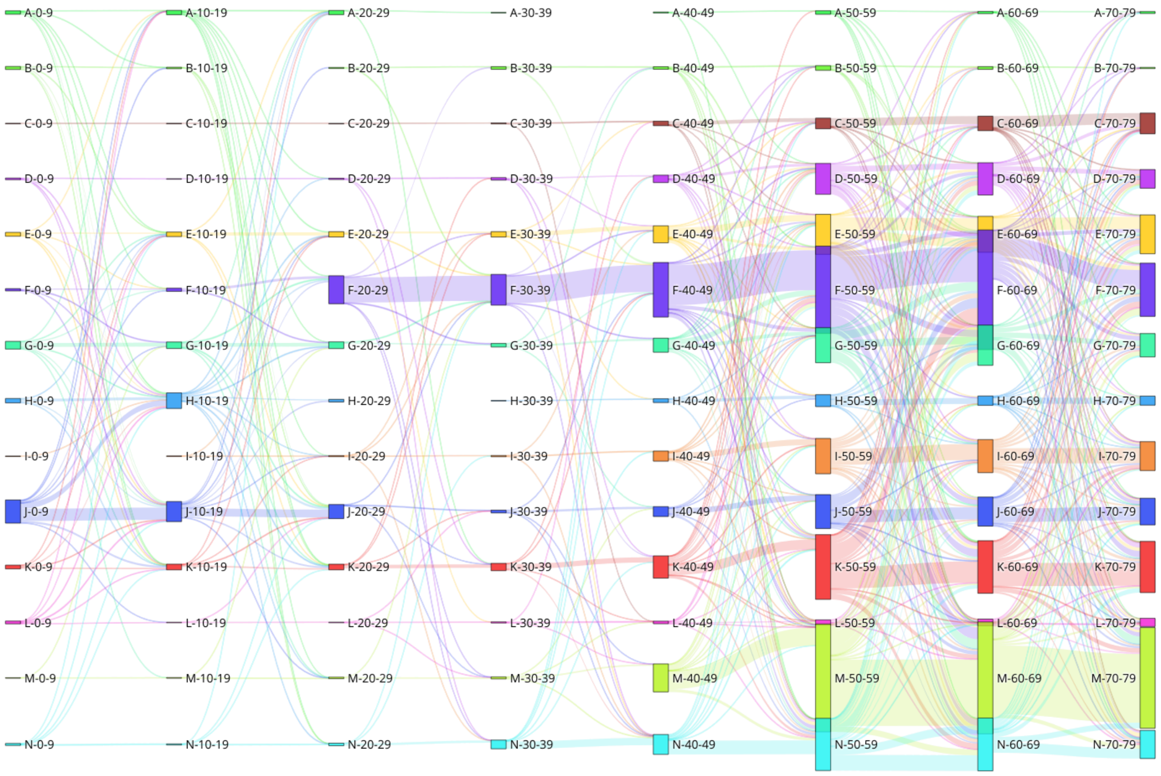

Supplementary Figure 2: Interlayer links grouped by nodesICD chapters, female patients. More detailed visualization of this plot can be found in the interactive WEB application: https://vis.csh.ac.at/comorbidity_network_graphics/mlcn_chapters.html

Visualisation tool

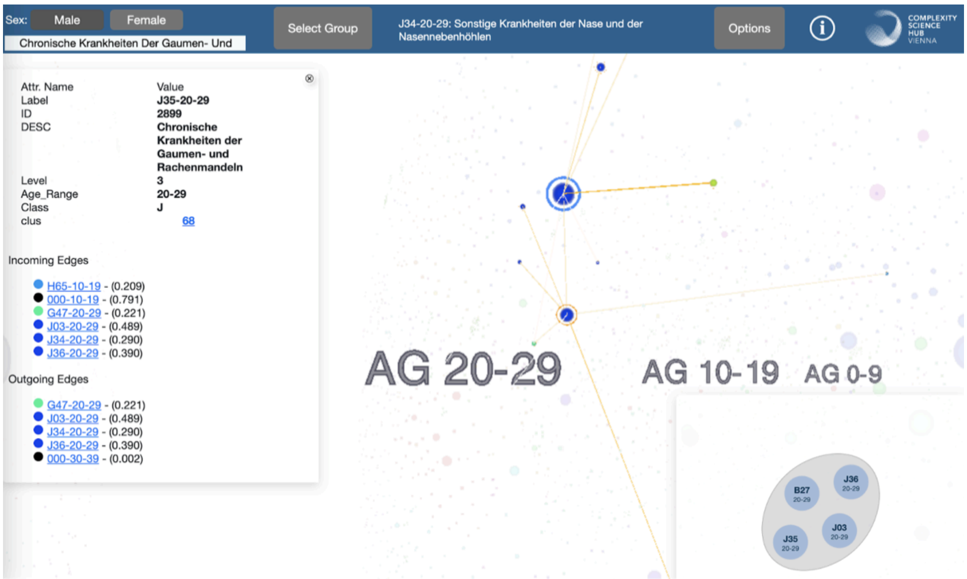

Supplementary Figure 3: Screenshot from developed visualisation tool

We designed and implemented an online visualization tool that allows a user to interactively explore the comorbidity network structure and the underlying diagnose data, https://vis.csh.ac.at/netviewer/. The tool positions the comorbidity network in a hierarchical fashion - one hierarchy layer for each age group; within each layer the nodes represent diagnoses, labelled by ICD10 code. A layer places nodes on a 2D plane within 3D space and signifies the associated age group by a label. The node color indicates the diagnose chapter; node size is scaled by the diagnosis prevalence of the node in the network. Connections between nodes indicate the likelihood that a pair of diseases is co-occurring in the link color (from white (unlikely) to orange (very likely)). A multitude of options to explore network properties and structure are at the user's disposal. A user can search for diagnoses (top left), or select one of the pre-computed communities (Select Group Button). When selecting a node or a community the camera in the main view automatically transforms to a position that puts all selected network elements into view. A two dimensional representation of the current community is displayed in the bottom right corner. When selecting a node in the main 3D view or in the 2D community view, information on detailed node attributes are displayed in the info box on the left. The info box also displays a list of a node's community memberships as well as incoming and outgoing edges of the currently selected node, along with the edge weights. Selecting a community or an edge automatically transforms the view to the selected element.

With this set of visual information and interactions, the user can easily browse node attributes, inspect community compositions or follow disease paths in the network.

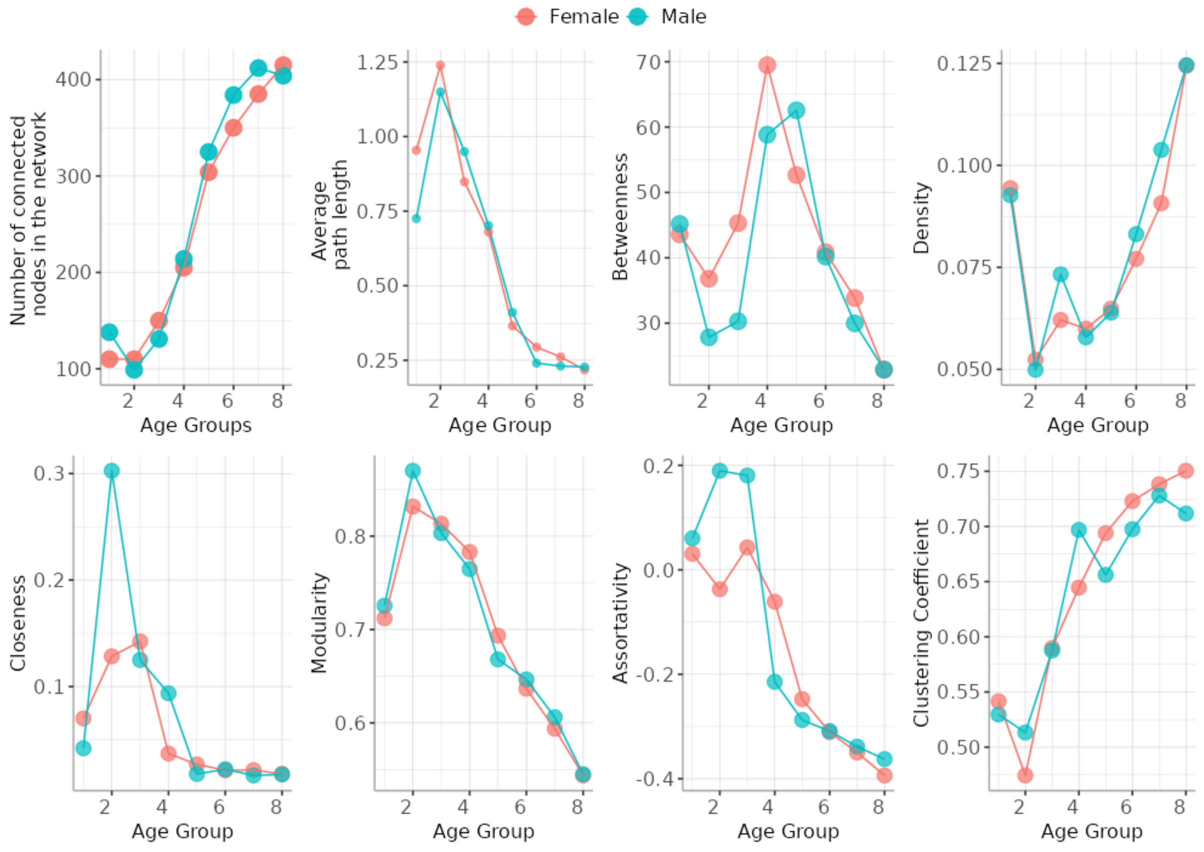

Supplementary Figure 4: Network properties

In Figure 4, we show the network properties (number of nodes, average path length, betweenness, density, closeness, modularity, assortativity, and clustering coefficient) for each age layer for males and females, respectively. It can be seen that the network undergoes a massive topological restructuring as the underlying patient cohorts age. The networks become increasingly dense with age (with the exception of the youngest age group), which is associated with an increase in the clustering coefficient and a decrease in the average path length. Together with the reduction of modularity, this suggests that for younger age groups, the networks consist mostly of well-separated disease communities, which become increasingly connected and bridged by high-degree nodes with increasing age, coinciding with a reversal from an assortative to a disassortative topology. Interestingly, the betweenness peaks around age 40-50, suggesting that this is the age at which the first bridges between the initially segregated clusters emerge. As the number of such bridges increases with age, the betweenness decreases. Overall, there are few differences between the properties of male and female networks, especially at older ages.

Supplementary Table 5: All identified communities in males. Communities smaller than 100 members and bigger than 2 members

Community_ID	Members of community
1	K35-10-19, K37-10-19, K56-10-19, K65-10-19
2	A04__0-9, A08__0-9, B09__0-9, F50__0-9, J10__0-9
3	A09__0-9, H73__0-9, K92__0-9
4	A41__0-9, C91__0-9, N10__0-9, A41-10-19, C91-10-19
5	A49__0-9, B27__0-9, J03__0-9, J06__0-9, J11__0-9, J38__0-9, L04__0-9, L50__0-9, J38-10-19
6	B00__0-9, B08__0-9, B34__0-9, B37__0-9, B99__0-9, D50__0-9, H10__0-9, J00__0-9, J02__0-9, J04__0-9, J05__0-9, J12__0-9, J15__0-9, J21__0-9, J22__0-9, J31__0-9, J38__0-9, J40__0-9, K52__0-9, L22__0-9
7	L20-30-39, L20-40-49, L20-50-59, L20-60-69
8	B27__0-9, J03__0-9, L04__0-9
9	D50-10-19, K50-10-19, D50-20-29
10	B97__0-9, J20__0-9, J30__0-9, J42__0-9, L30__0-9, D80-10-19
11	C71__0-9, C79-10-19, H53-10-19
12	D61__0-9, D69__0-9, D70__0-9
13	D80__0-9, E45__0-9, E61__0-9, G00__0-9, H02__0-9, H04__0-9, J01__0-9, J16__0-9, K00__0-9, K76__0-9, L27__0-9, M43__0-9, N44__0-9, H65-10-19, J35-10-19, J36-10-19, H65-20-29
14	E03__0-9, E03-10-19, E03-20-29
15	E10__0-9, E14__0-9, E16__0-9, E10-10-19, E14-10-19, E16-10-19
16	E23__0-9, E23-10-19, E23-20-29
17	E34__0-9, E34-10-19, E34-20-29
18	E66__0-9, E66-10-19, E78-10-19, F17-10-19, I10-10-19, K76-10-19
19	E84__0-9, E84-10-19, K86-10-19, E84-20-29, K86-20-29, E84-30-39
20	E88__0-9, J96-10-19, J96-20-29
21	F41__0-9, K02__0-9, K05__0-9, K10__0-9, L03__0-9
22	F43__0-9, F81__0-9, F43-10-19, F70-10-19, F81-10-19, F90-10-19, F93-10-19
23	E88__0-9, F79__0-9, G25__0-9, G41__0-9, I47__0-9, J69__0-9, G40-10-19, G41-10-19, J96-10-19, J96-20-29
24	F80__0-9, F82__0-9, F80-10-19, F82-10-19
25	F90__0-9, F91__0-9, F92__0-9, F93__0-9
26	F91__0-9, F91-10-19, F91-20-29
27	G40__0-9, G41__0-9, G47__0-9, G81__0-9, G91__0-9, G41-10-19, G47-10-19, G81-10-19, G91-10-19
28	G71__0-9, G71-10-19, G71-20-29
29	M22-10-19, M23-10-19, M24-10-19, M25-10-19, M65-10-19, M67-10-19
30	G93-10-19, G93-20-29, G93-30-39
31	H50__0-9, H52__0-9, H53__0-9, H50-10-19, H52-10-19
32	H60__0-9, H66__0-9, H70__0-9, H92__0-9, J11__0-9, K04__0-9
33	H61__0-9, H65__0-9, H74__0-9, J34__0-9, J39__0-9
34	H68__0-9, H69__0-9, J32__0-9, J35__0-9, J36__0-9
35	H70__0-9, K04__0-9, K08__0-9, K12__0-9
36	H90__0-9, H91__0-9, H90-10-19, H91-10-19
37	J30__0-9, J30-10-19, J30-20-29
38	J04__0-9, J05__0-9, J31__0-9, J40__0-9, J31-10-19

39	K21__0-9, K21-10-19, K29-10-19, K44-10-19, K52-10-19
40	J45__0-9, J46__0-9, J46-10-19
41	K40__0-9, K42__0-9, N43__0-9, K40-10-19, N43-10-19
42	K59__0-9, K60__0-9, K61__0-9
43	K90__0-9, K90-10-19, K90-20-29, K90-30-39
44	L01__0-9, L20__0-9, L20-10-19, L20-20-29, L20-30-39, L20-40-49, L20-50-59, L20-60-69
45	L90__0-9, N35__0-9, N47__0-9, N48__0-9
46	M21__0-9, M21-10-19, M21-20-29
47	N12__0-9, N39__0-9, N39-10-19, N39-20-29
48	N10__0-9, N13__0-9, N28__0-9, N28-10-19
49	N31__0-9, N31-10-19, N31-20-29
50	B27-10-19, J03-10-19, J36-10-19
51	C71-10-19, C71-20-29, C71-30-39, D43-30-39
52	D69-10-19, D69-20-29, D69-30-39
53	E84-10-19, E84-20-29, K86-20-29, E84-30-39
54	E87-10-19, F10-10-19, F17-10-19
55	F11-10-19, F13-10-19, F19-10-19
56	F32-10-19, F33-10-19, F41-10-19, F45-10-19
57	E87-10-19, F10-10-19, F12-10-19, F17-10-19, F20-10-19, F23-10-19, F60-10-19, F60-20-29, F63-20-29
58	F70-10-19, F70-20-29, F70-30-39, F70-40-49
59	F91__0-9, F90-10-19, F91-10-19, F93-10-19, F98-10-19, F91-20-29
60	G80-10-19, G82-10-19, G80-20-29, G82-20-29, G80-30-39, G80-40-49
61	I10-10-19, I10-20-29, I15-30-39, I15-40-49
62	F80__0-9, F82__0-9, F83__0-9, F84__0-9, F89__0-9, F80-10-19, F82-10-19, F89-10-19, F89-20-29
63	J32-10-19, J33-10-19, J34-10-19, M95-10-19
64	K76-10-19, K76-20-29, K76-30-39, K65-40-49
65	N44-10-19, N45-10-19, N50-10-19
66	A63-20-29, N47-20-29, N48-20-29
67	B20-30-39, B24-30-39, B20-40-49, B24-40-49
68	B27-20-29, J03-20-29, J35-20-29, J36-20-29
69	F40-30-39, F40-40-49, F40-50-59
70	C62-20-29, C77-20-29, C78-20-29, D40-20-29
71	D16-20-29, L05-20-29, D16-30-39, L05-30-39
72	N18__0-9, N18-10-19, N18-20-29, D64-30-39, N19-30-39
73	E10__0-9, E14__0-9, E16__0-9, E10-10-19, E14-10-19, E16-10-19, E10-20-29, E14-20-29, E16-20-29
74	E11-20-29, E66-20-29, E78-20-29, E79-20-29, G47-20-29, G47-30-39
75	E78-20-29, E79-20-29, E79-30-39
76	F10-20-29, K70-20-29, K71-20-29, K71-30-39
77	F11-10-19, F13-10-19, F19-10-19, B17-20-29, B18-20-29, B24-20-29, F11-20-29, F13-20-29, F14-20-29, B94-30-39, F15-30-39
78	F20-10-19, F23-10-19, F12-20-29, F15-20-29, F20-20-29, F23-20-29, F25-20-29, F31-20-29, F20-30-39, F23-30-39, F25-30-39, F20-40-49, F25-40-49

79	B94-20-29, B16-30-39, B17-30-39, B18-30-39, F11-30-39, F12-30-39, F13-30-39, F14-30-39, F19-30-39, K73-30-39, B17-40-49, B18-40-49, B94-40-49, F11-40-49, F12-40-49, F14-40-49, F19-40-49, K73-40-49, F12-50-59, K73-50-59
80	F17-20-29, J06-20-29, J93-20-29
81	F19-20-29, F90-20-29, B94-30-39
82	F20-10-19, F23-10-19, F20-20-29, F23-20-29, F25-20-29, F20-30-39, F23-30-39, F25-30-39
83	F31-20-29, F31-30-39, F31-40-49, F31-50-59, F31-60-69, F31-70-79
84	F32-20-29, F40-20-29, F63-20-29
85	F41-20-29, F45-20-29, F41-30-39, F45-30-39
86	F42-20-29, F42-30-39, F42-40-49
87	F20-10-19, F23-10-19, F20-20-29, F23-20-29, F25-20-29, F20-30-39, F23-30-39, F25-30-39, F20-40-49, F25-40-49
88	G80-20-29, G80-30-39, G80-40-49
89	G82-20-29, G82-30-39, G82-40-49
90	H83-20-29, H91-20-29, H93-20-29
91	I26-20-29, I80-20-29, I26-30-39, I80-30-39
92	I42-20-29, I42-30-39, I50-30-39
93	J01-20-29, J32-20-29, J33-20-29
94	I26-20-29, I80-20-29, J18-20-29, J90-20-29, I26-30-39, I80-30-39
95	K01-20-29, K07-20-29, K09-20-29
96	K02-20-29, K04-20-29, K08-20-29, K12-20-29
97	K03-20-29, K01-30-39, K02-30-39, K04-30-39, K07-30-39, K08-30-39, K09-30-39, K12-30-39
98	K51-10-19, K25-20-29, K26-20-29, K29-20-29, K51-20-29, K58-20-29, K59-20-29
99	K35-20-29, K37-20-29, K65-20-29
100	D50-10-19, K50-10-19, D50-20-29, K50-20-29, K56-20-29, K63-20-29, K56-30-39
101	L02-20-29, L03-20-29, L72-20-29
102	K74-20-29, I85-30-39, K74-30-39
103	K80-20-29, K81-20-29, K85-20-29
104	M17-20-29, M23-20-29, M24-20-29, M25-20-29, M65-20-29, M67-20-29, M93-20-29, M94-20-29
105	M24-20-29, M25-20-29, M65-20-29, M67-20-29
106	M42-20-29, M51-20-29, M53-20-29, M54-20-29
107	M81-20-29, M81-30-39, M81-40-49
108	N19-20-29, I10-30-39, I11-30-39, I34-30-39, I61-30-39, I67-30-39, I70-30-39, I71-40-49, L97-40-49
109	N13-20-29, N20-20-29, N23-20-29, N13-30-39, N20-30-39, N23-30-39
110	N18__0-9, N18-10-19, N18-20-29, D64-30-39
111	B16-30-39, K73-30-39, B17-40-49, B18-40-49, K73-40-49, B17-50-59, B18-50-59, C22-50-59, K73-50-59, B17-60-69, K73-60-69
112	B16-30-39, B17-30-39, B18-30-39, K73-30-39, B17-40-49, B18-40-49, B94-40-49, K73-40-49, B17-50-59, B18-50-59, C22-50-59, K73-50-59, B17-60-69, K73-60-69
113	C34-30-39, C34-40-49, D38-40-49
114	C62-30-39, C77-30-39, C78-30-39, C79-30-39, D40-30-39, C62-40-49, C77-40-49, C62-50-59, C62-60-69
115	C73-30-39, C73-40-49, E89-40-49
116	C81-40-49, C81-50-59, C81-60-69, C81-70-79
117	I84-20-29, K62-20-29, D12-30-39, I84-30-39, K62-30-39

118	F70-10-19, F70-20-29, D53-30-39, F10-30-39, F34-30-39, F70-30-39, D53-40-49, F05-40-49, F34-40-49, F70-40-49, G31-40-49, H10-40-49, K72-40-49, L21-40-49, D53-50-59
119	F70-10-19, F70-20-29, D75-30-39, F70-30-39, D52-40-49, F10-40-49, F22-40-49, F23-40-49, F70-40-49, G31-40-49, L21-40-49, C09-50-59, C10-50-59, C13-50-59, E53-50-59, F22-50-59, F55-50-59, H10-50-59, I86-50-59, N62-50-59, F22-60-69, F22-70-79
120	D86-30-39, D86-40-49, D86-50-59
121	E04-30-39, E05-30-39, E04-40-49, E05-40-49, E89-50-59
122	E10-30-39, E16-30-39, H36-30-39, E10-40-49, E16-40-49, H36-40-49
123	E13-30-39, H40-30-39, E11-40-49, E13-40-49, G63-40-49, H25-40-49, H26-40-49, H40-40-49, M86-40-49, N47-40-49, N48-40-49, I79-50-59, L03-50-59, M86-50-59
124	I83-30-39, I87-30-39, I83-40-49, I87-40-49, I87-50-59
125	K29-30-39, K31-30-39, K51-30-39, K58-30-39, K59-30-39, K59-40-49
126	G35-20-29, E78-30-39, E87-30-39, G35-30-39, G45-30-39, H81-30-39, M42-30-39, M47-30-39, M47-40-49
127	E88-30-39, E88-40-49, E88-50-59
128	F07-20-29, F07-30-39, F06-40-49, F07-40-49
129	B94-20-29, F17-30-39, G45-30-39, J06-30-39, J15-30-39, J20-30-39, J93-30-39, L05-40-49
130	F63-30-39, F63-40-49, F63-50-59
131	F61-40-49, F11-50-59, F13-50-59, F33-50-59, F43-50-59, F60-50-59, F61-50-59, F11-60-69, F60-60-69
132	G25-30-39, G25-40-49, G25-50-59
133	G40-40-49, G41-40-49, G81-40-49, J69-40-49, F70-50-59, G41-50-59, F70-60-69
134	M42-20-29, M51-20-29, M53-20-29, M54-20-29, G54-30-39, M54-30-39, M62-30-39, M99-30-39
135	H33-30-39, H35-30-39, H43-40-49, H33-40-49
136	H33-30-39, H35-30-39, H36-30-39, H43-40-49, H33-40-49, H34-40-49, H35-40-49, H36-40-49, H43-50-59, H33-50-59, H35-50-59, H36-50-59, H43-60-69, H26-60-69, H33-60-69
137	H50-30-39, H52-30-39, H53-30-39, H52-40-49, H53-40-49, H52-50-59, H53-50-59
138	H53-30-39, H53-40-49, H53-50-59, H53-60-69, H53-70-79
139	H91-30-39, H93-30-39, H90-40-49, H91-40-49, H93-40-49, H90-50-59, H91-50-59, H93-50-59, H90-60-69, H91-60-69, H93-60-69
140	I20-30-39, I21-30-39, I25-30-39, I20-40-49, I21-40-49, I24-40-49
141	I34-30-39, I07-40-49, I34-40-49, I36-40-49
142	I35-30-39, I71-30-39, I35-40-49, I71-40-49, I35-50-59, I71-50-59
143	I45-30-39, I45-40-49, I45-50-59
144	I73-30-39, I73-40-49, I74-40-49
145	J01-30-39, J32-30-39, J33-30-39, J01-40-49, J32-40-49, J33-40-49
146	J03-30-39, J35-30-39, J36-30-39
147	J18-30-39, J90-30-39, J96-30-39, J15-40-49
148	J30-30-39, J30-40-49, J30-50-59, J30-60-69
149	J44-30-39, J44-40-49, J98-40-49, J98-50-59
150	K01-30-39, K07-30-39, K09-30-39
151	K20-30-39, K21-40-49, K25-40-49, K26-40-49, D62-50-59, K20-50-59, K25-50-59, K26-50-59, K20-60-69, K20-70-79
152	K21-30-39, K25-30-39, K26-30-39, K31-30-39
153	K40-30-39, K42-30-39, K43-30-39
154	D50-30-39, K50-30-39, K56-30-39, D50-40-49, K50-40-49, K56-40-49, K50-50-59, K50-60-69, K50-70-79
155	K60-20-29, K61-20-29, L02-20-29, L03-20-29, L72-20-29, K60-30-39, K61-30-39

156	K63-30-39, D12-40-49, I84-40-49, K60-40-49, K61-40-49, K62-40-49, K63-40-49, D12-50-59, I84-50-59, K60-50-59, K61-50-59, K62-50-59, K64-50-59, D21-60-69, K64-60-69
157	K80-30-39, K81-30-39, K82-30-39, K83-30-39
158	K92-30-39, D62-40-49, K92-40-49
159	D17-30-39, L02-30-39, L03-30-39, L72-30-39
160	M16-30-39, M87-30-39, M16-40-49, M87-40-49
161	M17-30-39, M23-30-39, M66-30-39, M71-30-39, M93-30-39
162	M75-30-39, M75-40-49, M77-40-49, M76-50-59, M77-50-59
163	M25-30-39, M25-40-49, M66-40-49, M87-40-49
164	M42-30-39, M47-30-39, M47-40-49
165	M43-30-39, M48-30-39, M54-40-49, G54-50-59, H61-50-59
166	M50-30-39, M53-30-39, M50-40-49
167	M19-30-39, M24-30-39, M25-30-39, M65-30-39, M67-30-39, M75-30-39, M19-40-49, M24-40-49, M65-40-49, M67-40-49, M75-40-49, M77-40-49, M66-50-59, M75-50-59, M76-50-59, M77-50-59
168	N18-40-49, B99-50-59, N03-50-59, N25-50-59, N26-50-59, N05-60-69, N26-60-69, N26-70-79
169	N31__0-9, N31-10-19, N31-20-29, N31-30-39, N39-30-39, N41-30-39
170	A46-30-39, B35-30-39, A46-40-49, B35-40-49, I89-50-59
171	B16-40-49, B16-50-59, B16-60-69
172	B37-40-49, J18-40-49, J86-40-49
173	C15-40-49, C16-40-49, C15-50-59, C16-50-59
174	C18-40-49, C19-40-49, C20-40-49
175	C21-40-49, C19-50-59, C20-50-59, C21-50-59, K91-50-59, C21-60-69, C21-70-79
176	K74-20-29, I85-30-39, K74-30-39, C22-40-49, I85-40-49, K31-40-49, K72-40-49, K74-40-49, B99-50-59, K72-50-59
177	C32-40-49, J37-40-49, J38-40-49, C32-50-59, J37-50-59, J38-50-59
178	C43-40-49, D22-40-49, C43-50-59, D22-50-59
179	C61-40-49, N32-40-49, N40-40-49, C61-50-59, D40-50-59, N32-50-59
180	C64-40-49, D41-40-49, C64-50-59, D41-50-59
181	C71-40-49, D43-40-49, C71-50-59, D43-50-59
182	C82-40-49, C83-40-49, C85-40-49, C82-50-59, C83-50-59, C85-50-59
183	C90-40-49, C90-50-59, C90-60-69, C90-70-79
184	C92-40-49, C92-50-59, C92-60-69, D46-60-69, C92-70-79, D46-70-79
185	D12-40-49, I84-40-49, K60-40-49, K61-40-49, K62-40-49, D12-50-59, I84-50-59, K60-50-59, K61-50-59, K62-50-59, K64-50-59, D21-60-69, K64-60-69
186	D13-40-49, K63-50-59, D12-60-69, D13-60-69, D13-70-79
187	D17-40-49, D23-40-49, D48-40-49
188	D18-40-49, D18-50-59, D18-60-69, D18-70-79
189	M45-20-29, E13-30-39, M45-30-39, D35-40-49, I10-40-49, I12-40-49, I72-40-49, I77-40-49, K20-40-49, L98-40-49, M45-40-49, N05-40-49, I12-50-59, I13-50-59, M45-50-59, N05-50-59, M45-60-69, N05-60-69, M45-70-79
190	M54-50-59, G54-60-69, L50-60-69, M71-60-69, M76-60-69
191	D68-40-49, I26-40-49, I80-40-49, I82-40-49, D68-50-59, I26-50-59, I80-50-59, I82-50-59, D68-60-69
192	D75-40-49, D75-50-59, D75-60-69, D75-70-79
193	E06-40-49, E06-50-59, E06-60-69, E06-70-79
194	E10-30-39, E16-30-39, H36-30-39, E16-40-49
195	E12-40-49, E78-40-49, G45-40-49, G51-40-49, H81-40-49, I72-40-49, I77-40-49, K75-40-49, G45-50-59

196	K60-40-49, K61-40-49, K60-50-59, K61-50-59
197	E88-30-39, E88-40-49, F54-40-49, H47-40-49, E66-50-59, E88-50-59, E61-60-69, L08-60-69
198	G47-20-29, G25-30-39, G47-30-39, E66-40-49, G25-40-49, G47-40-49, G25-50-59, H66-50-59, K46-50-59
199	E73-40-49, E73-50-59, E73-60-69, E73-70-79
200	F09-40-49, F63-40-49, F03-50-59, F10-50-59, F63-50-59, G31-50-59, L21-50-59, D52-60-69, E51-60-69, E53-60-69, I62-60-69, J39-60-69, K02-60-69, K12-60-69, M46-60-69
201	J37-40-49, J38-40-49, J37-50-59, J38-50-59, J37-60-69, J37-70-79
202	F22-50-59, F22-60-69, F22-70-79
203	F32-40-49, F48-40-49, B37-50-59, F51-50-59, K02-50-59, K04-50-59, K08-50-59
204	F34-40-49, F34-50-59, F34-60-69, F34-70-79
205	D52-40-49, F54-40-49, F55-40-49, J41-40-49, L71-40-49, F17-50-59, J41-50-59, J42-50-59, J95-50-59, J13-60-69, J39-60-69, K04-60-69, K08-60-69, K12-60-69, L08-60-69, N62-60-69
206	F79-50-59, F79-60-69, F79-70-79
207	G35-20-29, G35-30-39, G35-40-49, N31-40-49, N31-50-59
208	G43-40-49, G43-50-59, G43-60-69
209	G46-40-49, I63-40-49, I64-40-49, I66-40-49
210	M43-30-39, M48-30-39, G55-40-49, M51-40-49, M54-40-49, M96-40-49, G54-50-59, G55-50-59, H61-50-59, M51-50-59, M96-50-59, M96-60-69
211	M96-40-49, M96-50-59, M96-60-69
212	F17-40-49, G58-40-49, J01-40-49, J37-40-49, J38-40-49, J42-40-49, J43-40-49, J93-40-49, J02-50-59, J37-50-59, J38-50-59, J37-60-69, J37-70-79
213	G91-40-49, I60-40-49, G91-50-59, I60-50-59
214	G95-40-49, G95-50-59, G95-60-69, G95-70-79
215	H40-30-39, H25-40-49, H26-40-49, H40-40-49
216	H91-30-39, H93-30-39, H81-40-49, H90-40-49, H91-40-49, H93-40-49, H81-50-59, H90-50-59, H91-50-59, H93-50-59
217	I20-30-39, I21-30-39, I25-30-39, I20-40-49, I21-40-49, I24-40-49, I25-40-49, I20-50-59, I21-50-59, I24-50-59, B00-60-69, I24-60-69
218	I12-40-49, I13-40-49, N25-40-49, D44-50-59, E72-50-59, G61-50-59, I10-50-59, I12-50-59, I13-50-59, I30-50-59, I62-50-59, I99-50-59, J22-50-59, J91-50-59, L23-50-59, L27-50-59, L73-50-59, M35-50-59, M46-50-59, M80-50-59, N05-50-59, C31-60-69, E26-60-69, L05-60-69, N05-60-69, N12-60-69
219	I25-40-49, I27-40-49, I31-40-49, A04-50-59, I27-50-59
220	I35-40-49, I71-40-49, I35-50-59, I71-50-59, I72-50-59, I71-60-69, I72-60-69, I71-70-79, I72-70-79
221	I42-20-29, I42-30-39, I50-30-39, I42-40-49, I44-40-49, I50-40-49, I51-40-49, I44-50-59
222	G93-10-19, G93-20-29, G93-30-39, G93-40-49, I46-40-49
223	G91-40-49, I60-40-49, I61-40-49, G91-50-59, I60-50-59, I61-50-59
224	I65-40-49, I65-50-59, G46-60-69, I66-60-69
225	D12-40-49, I84-40-49, K60-40-49, K61-40-49, K60-50-59, K61-50-59, K64-50-59
226	I98-40-49, I98-50-59, K74-50-59, I86-60-69
227	J34-40-49, J35-40-49, J36-40-49, M95-40-49
228	J41-40-49, J44-50-59, J47-50-59, J95-50-59, B90-60-69, G61-60-69, H10-60-69, J40-60-69, J47-60-69, J82-60-69, J95-60-69
229	J43-40-49, J43-50-59, J93-60-69
230	K02-50-59, K04-50-59, K08-50-59
231	K02-40-49, K04-40-49, K08-40-49, K12-40-49, K02-50-59, K04-50-59, K08-50-59

232	K20-30-39, K22-30-39, K21-40-49, K22-40-49, K25-40-49, K26-40-49, K44-40-49, K20-50-59, K26-50-59, K20-60-69, K20-70-79
233	K25-40-49, K26-40-49, K26-50-59
234	K40-40-49, K66-40-49, N43-40-49
235	K42-40-49, K43-40-49, D17-50-59, K40-50-59, K42-50-59, K43-50-59, K66-50-59, L72-50-59
236	K52-30-39, A09-40-49, E86-40-49, K52-40-49
237	K58-40-49, K58-50-59, K58-60-69, K58-70-79
238	K25-40-49, K26-40-49, K70-40-49, D52-50-59, D62-50-59, F05-50-59, I98-50-59, K25-50-59, K26-50-59
239	K76-40-49, A04-50-59, D61-50-59, E55-50-59, K46-50-59, K55-50-59, K75-50-59, K55-60-69
240	K80-40-49, K81-40-49, K82-40-49, K83-40-49, K81-50-59
241	K90-40-49, K90-50-59, K90-60-69, K90-70-79
242	L02-40-49, L03-40-49, L72-40-49
243	A46-30-39, B35-30-39, A46-40-49, B35-40-49, A46-50-59, B35-50-59, I89-50-59, L30-50-59, B35-60-69, L30-60-69
244	L71-40-49, K70-50-59, D53-60-69, F05-60-69, G31-60-69, G31-70-79
245	L89-40-49, L89-50-59, L89-60-69
246	L97-40-49, L98-40-49, L97-50-59, L98-50-59
247	M11-40-49, M23-40-49, M71-40-49, M93-40-49
248	M11-40-49, M17-40-49, M21-40-49, M22-40-49, M23-40-49, M71-40-49, M93-40-49, M94-40-49, M21-50-59, M22-50-59, M94-50-59
249	M20-40-49, M20-50-59, M20-60-69, M20-70-79
250	M31-40-49, M31-50-59, M31-60-69, M31-70-79
251	M42-30-39, M47-30-39, M41-40-49, M42-40-49, M43-40-49, M47-40-49, M48-40-49, M41-50-59, M42-50-59, M43-50-59, M48-50-59
252	M50-30-39, M53-30-39, M50-40-49, M53-40-49, M62-40-49, M62-50-59
253	M70-40-49, E79-50-59, M16-50-59, M70-50-59, M87-50-59, M87-60-69
254	M72-40-49, M72-50-59, M72-60-69
255	M77-40-49, M76-50-59, M77-50-59
256	L98-40-49, M86-40-49, L98-50-59, M86-50-59
257	N03-40-49, A49-50-59, A69-50-59, E78-50-59, F54-50-59, G46-50-59, G58-50-59, H49-50-59, H81-50-59, I05-50-59, I66-50-59, I99-50-59, L27-50-59, M15-50-59, F23-60-69, F44-60-69, J03-60-69
258	N04-40-49, N04-50-59, N04-60-69
259	N47-60-69, N48-60-69, N47-70-79, N48-70-79
260	N13-40-49, N20-40-49, N23-40-49, N13-50-59, N20-50-59, N23-50-59, N20-60-69, N21-60-69, N23-60-69
261	N25-40-49, N26-40-49, N18-50-59, N25-50-59, N26-50-59, A04-60-69, B02-60-69, N03-60-69, N26-60-69, N26-70-79
262	F54-40-49, N26-40-49, I25-50-59, I97-50-59, I13-60-69, I30-60-69, I33-60-69, K04-60-69
263	N28-40-49, N28-50-59, N28-60-69
264	N39-50-59, N45-50-59, A04-60-69, N30-60-69, N45-60-69
265	N32-40-49, N35-40-49, N32-50-59, N35-50-59
266	N39-40-49, N45-40-49, I95-50-59
267	N41-40-49, N41-50-59, N42-50-59
268	A08-50-59, I25-60-69, I97-60-69, I99-60-69, J22-60-69, B34-70-79, C75-70-79, D14-70-79, F51-70-79, F52-70-79, G12-70-79, G98-70-79, J85-70-79, K02-70-79
269	A41-50-59, J80-50-59, K65-50-59

270	A49-50-59, K29-50-59, K50-50-59, K51-50-59, K75-50-59, D73-60-69, E61-60-69, K50-60-69, L50-60-69, N62-60-69, K50-70-79
271	B02-50-59, J18-60-69, J86-60-69, B00-70-79, B96-70-79, C79-70-79, D72-70-79, D73-70-79, M84-70-79
272	B16-30-39, K73-30-39, B17-40-49, B18-40-49, K73-40-49, B17-50-59, B18-50-59, C22-50-59, F19-50-59, K73-50-59, B17-60-69, F19-60-69, K73-60-69
273	B37-50-59, C34-50-59, C38-50-59, C71-50-59, D43-50-59, J82-50-59, J93-50-59
274	B90-50-59, J47-50-59, J44-60-69, J47-60-69, A16-70-79, B44-70-79, B90-70-79, D52-70-79, G58-70-79, J04-70-79, J21-70-79, J39-70-79, L27-70-79, L28-70-79, M00-70-79
275	C04-50-59, C04-60-69, C04-70-79
276	C09-50-59, C09-60-69, C10-60-69, C09-70-79, C10-70-79
277	C18-40-49, C19-40-49, C20-40-49, C18-50-59, C19-50-59
278	C21-50-59, C21-60-69, C21-70-79
279	C21-50-59, C18-60-69, C19-60-69, C20-60-69, C21-60-69, C48-60-69, K36-60-69, K91-60-69, C19-70-79, C20-70-79, C21-70-79, C48-70-79, K36-70-79, K91-70-79
280	C49-50-59, C49-60-69, C49-70-79
281	C67-40-49, C67-50-59, C68-50-59, C67-60-69, C68-60-69, D41-60-69, N30-60-69, C65-70-79, C68-70-79
282	C71-50-59, D43-50-59, C71-60-69, D43-60-69, C71-70-79, D43-70-79
283	C73-50-59, E89-50-59, C73-60-69, E89-60-69, C73-70-79, E89-70-79
284	D17-50-59, K40-50-59, K66-50-59, L72-50-59
285	D32-50-59, D32-60-69, D32-70-79
286	D35-50-59, D35-60-69, D35-70-79
287	D47-50-59, D47-60-69, D47-70-79
288	D59-50-59, D59-60-69, D59-70-79
289	E83-40-49, D64-50-59, E03-50-59, E21-50-59, E83-50-59, A49-60-69, E83-60-69, E83-70-79
290	K51-30-39, K51-40-49, D73-50-59, J04-50-59, K51-50-59, B96-60-69, K29-60-69, K51-60-69, A69-70-79, D16-70-79, D21-70-79, K51-70-79, L50-70-79, N10-70-79, N62-70-79
291	L89-40-49, N08-40-49, E11-50-59, E12-50-59, E55-50-59, L89-50-59, G61-60-69, I13-60-69, I33-60-69, L28-60-69, L89-60-69
292	E10-50-59, E13-50-59, E16-50-59, E16-60-69
293	E83-40-49, E83-50-59, E83-60-69, E83-70-79
294	G93-10-19, G93-20-29, G93-30-39, G93-40-49, I46-40-49, G93-50-59, I46-50-59
295	F07-20-29, F07-30-39, F06-40-49, F07-40-49, F06-50-59, F07-50-59
296	H53-30-39, H52-40-49, H53-40-49, H52-50-59, H53-50-59, H43-60-69, H02-60-69, H26-60-69, H33-60-69, H52-60-69, H53-60-69, H43-70-79, H02-70-79, H26-70-79, H33-70-79, H52-70-79, H53-70-79
297	G35-60-69, N47-60-69, N48-60-69, G35-70-79, N47-70-79, N48-70-79
298	G44-30-39, F45-40-49, G44-40-49, F45-50-59, G44-50-59
299	G47-50-59, J30-50-59, F51-60-69, J30-60-69
300	G91-40-49, I60-40-49, G91-50-59, I60-50-59, G91-60-69, G91-70-79
301	G20-50-59, F02-60-69, G20-60-69, G21-60-69, G21-70-79
302	G30-50-59, F00-60-69, G30-60-69
303	G40-50-59, G41-50-59, F01-60-69, F71-60-69, G41-60-69
304	G51-50-59, G51-60-69, G51-70-79
305	M43-30-39, M48-30-39, M51-40-49, M54-40-49, M96-40-49, G54-50-59, H61-50-59, M96-50-59, M96-60-69
306	G82-20-29, G82-30-39, G82-40-49, G82-50-59, G83-60-69

307	G99-50-59, J31-50-59, J94-50-59, K45-50-59, E66-60-69, G62-60-69, G61-70-79, H16-70-79, L71-70-79, M93-70-79
308	H02-50-59, H25-50-59, H26-50-59, H27-50-59, H34-50-59, H34-60-69, H34-70-79
309	H34-50-59, H34-60-69, H34-70-79
310	H40-50-59, H47-50-59, H40-60-69, H47-60-69, H40-70-79, H47-70-79
311	H44-50-59, H44-60-69, H44-70-79
312	H54-50-59, H54-60-69, H54-70-79
313	H90-50-59, H91-50-59, H93-50-59, H90-60-69, H91-60-69, H93-60-69, H90-70-79, H91-70-79, H93-70-79
314	I07-50-59, I34-50-59, I36-50-59, I07-60-69, I08-60-69, I34-60-69, I36-60-69, I07-70-79, I34-70-79, I36-70-79
315	I20-50-59, B00-60-69, K04-60-69
316	I31-50-59, I31-60-69, I31-70-79
317	I34-50-59, I36-50-59, I08-60-69
318	I48-20-29, I48-30-39, I42-40-49, I44-40-49, I48-40-49, I07-50-59, I34-50-59, I36-50-59, I44-50-59, I08-60-69
319	I48-50-59, I05-60-69, I05-70-79
320	I50-50-59, J81-50-59, B99-60-69, G93-60-69, I46-60-69
321	I77-50-59, I77-60-69, I77-70-79
322	I71-50-59, I72-50-59, I71-60-69, I72-60-69, I71-70-79, I72-70-79
323	K60-40-49, K61-40-49, I84-50-59, K60-50-59, K61-50-59, D21-60-69
324	J01-50-59, J32-50-59, J33-50-59, J01-60-69
325	B37-40-49, J18-50-59, J40-50-59, J86-50-59, J93-50-59, B02-60-69, L72-60-69
326	J32-60-69, J33-60-69, J34-60-69, J32-70-79, J33-70-79, J34-70-79
327	J34-50-59, J35-50-59, M95-50-59
328	K20-50-59, K20-60-69, K20-70-79
329	C44-40-49, C44-50-59, L57-50-59, C44-60-69, D23-60-69, L57-60-69, L57-70-79
330	D12-60-69, D13-60-69, D13-70-79
331	K55-50-59, K55-60-69, K55-70-79
332	K59-30-39, K59-40-49, K56-50-59, K59-50-59, K65-50-59, K91-50-59
333	K80-50-59, K81-50-59, K82-50-59
334	L28-50-59, K76-60-69, B96-70-79, D73-70-79, E51-70-79, G58-70-79, M89-70-79
335	L57-50-59, L57-60-69, L57-70-79
336	M07-50-59, L40-60-69, L40-70-79
337	M15-50-59, M15-60-69, M15-70-79
338	M22-70-79, M23-70-79, M24-70-79, M67-70-79, M94-70-79
339	M35-50-59, M35-60-69, M35-70-79
340	M42-30-39, M47-30-39, M42-40-49, M43-40-49, M47-40-49, M48-40-49, M43-50-59, M48-50-59
341	M45-20-29, M45-30-39, M45-40-49, M45-50-59, M45-60-69, M45-70-79
342	M81-50-59, M80-60-69, M85-60-69, M80-70-79
343	M89-50-59, F32-60-69, F60-70-79, G83-70-79
344	M17-40-49, M21-40-49, M22-40-49, M17-50-59, M21-50-59, M22-50-59
345	N08-50-59, N08-60-69, N08-70-79
346	N10-50-59, N26-50-59, N18-60-69, N26-60-69, B02-70-79, B25-70-79, H10-70-79, I13-70-79, N05-70-79, N26-70-79
347	N13-50-59, N20-50-59, N23-50-59, N13-60-69, N20-60-69, N21-60-69, N23-60-69, N21-70-79

348	N32-40-49, N40-40-49, N41-40-49, N21-50-59, N40-50-59, N41-50-59, N42-50-59, N41-60-69, N42-60-69
349	N26-50-59, N26-60-69, N26-70-79
350	N43-50-59, N43-60-69, N43-70-79
351	A02-60-69, A18-60-69, A26-60-69, A40-60-69, A48-60-69, A69-60-69, A84-60-69, B07-60-69, B36-60-69, B44-60-69, B49-60-69, B86-60-69, B91-60-69, C00-60-69, C06-60-69, C11-60-69, C12-60-69, C14-60-69, C26-60-69, C39-60-69, C41-60-69, C45-60-69, C50-60-69, C60-60-69, C66-60-69, C69-60-69, C76-60-69, C84-60-69, C88-60-69, D01-60-69, D03-60-69, D04-60-69, D07-60-69, D11-60-69, D15-60-69, D31-60-69, D34-60-69, D51-60-69, D89-60-69, E07-60-69, E22-60-69, E23-60-69, E27-60-69, E41-60-69, E46-60-69, E74-60-69, E85-60-69, F52-60-69, G06-60-69, G24-60-69, G52-60-69, G90-60-69, G92-60-69, H21-60-69, H31-60-69, H55-60-69, H61-60-69, H70-60-69, J02-60-69, J10-60-69, J11-60-69, J12-60-69, J21-60-69, J61-60-69, J85-60-69, K11-60-69, K28-60-69, K41-60-69, L25-60-69, L58-60-69, L60-60-69, L80-60-69, L81-60-69, M12-60-69, M60-60-69, N11-60-69, N34-60-69, N36-60-69, N50-60-69, 000-70-79
352	K57-30-39, K57-40-49, K57-50-59, A08-60-69
353	G20-50-59, F02-60-69, G20-60-69, G21-60-69, F02-70-79, G20-70-79, G21-70-79
354	A41-60-69, B37-60-69, J80-60-69
355	A46-60-69, B35-60-69, A46-70-79, B35-70-79
356	B18-60-69, C22-60-69, C24-60-69, K73-70-79
357	C01-50-59, C02-50-59, C01-60-69, C02-60-69, C01-70-79, C02-70-79
358	C01-60-69, C02-60-69, C01-70-79, C02-70-79
359	C13-60-69, C32-60-69, J38-60-69, C13-70-79, C32-70-79, J38-70-79
360	C15-40-49, C16-40-49, C15-50-59, C16-50-59, C15-60-69, C16-60-69
361	C25-40-49, C25-50-59, C24-60-69, C25-60-69, C25-70-79
362	C71-60-69, D43-60-69, C71-70-79, D43-70-79
363	C65-60-69, D30-60-69, C66-70-79, C67-70-79, C68-70-79
364	C65-60-69, C67-60-69, C68-60-69, D30-60-69, N30-60-69, C65-70-79, C66-70-79, C67-70-79, C68-70-79
365	G82-50-59, C79-60-69, G82-60-69, G83-60-69, M84-60-69, G82-70-79
366	C91-40-49, C91-50-59, C82-60-69, C83-60-69, C85-60-69, C91-60-69, D72-60-69, C82-70-79, C83-70-79, C85-70-79, C91-70-79, D80-70-79
367	C91-40-49, C91-50-59, C91-60-69, D80-70-79
368	D17-60-69, K40-60-69, K46-60-69
369	N43-50-59, D29-60-69, D30-60-69, N43-60-69, N45-60-69, D30-70-79, N02-70-79, N39-70-79, N40-70-79, N43-70-79, N45-70-79, N99-70-79
370	D72-60-69, C83-70-79, C85-70-79, C91-70-79
371	D86-30-39, D86-40-49, D86-50-59, D86-60-69, D86-70-79
372	E12-50-59, E12-60-69, E12-70-79
373	E10-50-59, E13-50-59, E16-50-59, E10-60-69, E13-60-69, E16-60-69, I79-60-69, E16-70-79, I79-70-79
374	E68-60-69, J16-60-69, L95-60-69, C45-70-79, D89-70-79, E01-70-79, E72-70-79, G90-70-79, I10-70-79, I22-70-79, I33-70-79, I60-70-79, I78-70-79, J04-70-79, J13-70-79, J16-70-79, K27-70-79, L08-70-79, L20-70-79, L21-70-79, L23-70-79, L29-70-79, L50-70-79, L71-70-79, L85-70-79, M05-70-79, M18-70-79, M31-70-79, M45-70-79, M85-70-79, M96-70-79, N62-70-79
375	E61-50-59, G57-50-59, E78-60-69, F34-60-69, G43-60-69, G44-60-69, G57-60-69, G58-60-69, G83-60-69, H49-60-69, I08-60-69, I22-60-69, I99-60-69, C75-70-79, D11-70-79, F40-70-79, G44-70-79, G71-70-79, H00-70-79, J85-70-79, K13-70-79
376	M70-50-59, E79-60-69, L82-60-69, M18-60-69, M70-60-69, H61-70-79, L21-70-79, M54-70-79, M70-70-79, M79-70-79
377	F06-60-69, F07-60-69, F06-70-79, F07-70-79
378	F10-60-69, F13-60-69, F31-60-69, F60-60-69, G31-60-69, D53-70-79, E51-70-79, F13-70-79, F31-70-79, G31-70-79, K08-70-79
379	F13-60-69, F33-60-69, F41-60-69, F43-60-69, F45-60-69, F13-70-79, F32-70-79, F33-70-79, F41-70-79, F43-70-79, F45-70-79

380	H43-60-69, H26-60-69, H33-60-69, H43-70-79, H26-70-79, H33-70-79
381	J04-50-59, F17-60-69, D51-70-79, H49-70-79, J82-70-79, K04-70-79, K12-70-79
382	F32-50-59, G20-50-59, G35-50-59, F48-60-69, F61-60-69, G35-60-69, K02-60-69, N47-60-69, N48-60-69, G35-70-79, N47-70-79, N48-70-79
383	F54-60-69, G60-60-69, H57-60-69, I38-60-69, F09-70-79, I25-70-79, I97-70-79, I99-70-79, J01-70-79, J41-70-79, M46-70-79
384	F01-50-59, G40-60-69, G41-60-69, I62-60-69, G40-70-79, G41-70-79
385	G82-50-59, G82-60-69, G83-60-69, G82-70-79
386	H90-50-59, H91-50-59, H93-50-59, H00-60-69, H16-60-69, H81-60-69, H90-60-69, H91-60-69, H93-60-69, I38-60-69, K30-60-69, M40-60-69, E78-70-79, F34-70-79, G57-70-79, H10-70-79, H81-70-79, H90-70-79, H91-70-79, H93-70-79, I06-70-79
387	H02-60-69, H02-70-79, H26-70-79
388	H44-50-59, H11-60-69, H17-60-69, H18-60-69, H25-60-69, H27-60-69, H44-60-69, H50-60-69, H11-70-79, H17-70-79, H18-70-79, H21-70-79, H27-70-79, H44-70-79
389	E10-70-79, E13-70-79, E16-70-79, H43-70-79, H26-70-79, H33-70-79, H35-70-79, H36-70-79
390	G91-40-49, I60-40-49, D73-50-59, F52-50-59, G30-50-59, G91-50-59, G99-50-59, I60-50-59, L28-50-59, E11-60-69, E55-60-69, F00-60-69, F05-60-69, G30-60-69, G91-60-69, H49-60-69, A26-70-79, A48-70-79, C76-70-79, D34-70-79, G91-70-79, H59-70-79, H60-70-79, L08-70-79, L28-70-79, M00-70-79, N04-70-79
391	H53-30-39, H52-40-49, H53-40-49, H52-50-59, H53-50-59, H52-60-69, H53-60-69, H52-70-79, H53-70-79
392	I77-50-59, H57-60-69, I77-60-69, I70-70-79, I73-70-79, I74-70-79, I77-70-79, L02-70-79, L03-70-79
393	H90-60-69, H93-60-69, H90-70-79, H91-70-79, H93-70-79
394	B36-60-69, D34-60-69, E72-60-69, F09-60-69, G90-60-69, I10-60-69, I30-60-69, I33-60-69, I60-60-69, I78-60-69, J13-60-69, J30-60-69, K73-60-69, L08-60-69, L21-60-69, L23-60-69, L27-60-69, L29-60-69, L71-60-69, L85-60-69, N02-60-69, N04-60-69, C05-70-79, C74-70-79, D15-70-79, E26-70-79, E29-70-79, F55-70-79, G37-70-79, G96-70-79, H72-70-79, I68-70-79, J35-70-79, K09-70-79, L70-70-79, M14-70-79, N03-70-79, N49-70-79
395	I26-60-69, I80-60-69, I82-60-69, I26-70-79, I80-70-79, I82-70-79
396	J84-40-49, J84-50-59, I07-60-69, I27-60-69, I34-60-69, I36-60-69, J84-60-69, I07-70-79, I27-70-79, I34-70-79, I36-70-79, J84-70-79
397	I48-60-69, A48-70-79, C76-70-79, H18-70-79, H61-70-79, I33-70-79, J30-70-79, K04-70-79
398	I08-50-59, I31-50-59, F54-60-69, G60-60-69, H57-60-69, I31-60-69, I38-60-69, I50-60-69, D52-70-79, F09-70-79, I13-70-79, I25-70-79, I31-70-79, I97-70-79, I99-70-79, J01-70-79, J41-70-79, M46-70-79
399	K60-60-69, K61-60-69, K60-70-79, K61-70-79
400	C15-60-69, C16-60-69, C15-70-79, C16-70-79
401	F09-60-69, J62-60-69, J92-60-69, J44-70-79, J47-70-79, J62-70-79, K20-70-79
402	C34-60-69, C38-60-69, C71-60-69, D43-60-69, J82-60-69, C71-70-79, D43-70-79
403	J84-40-49, J84-50-59, J84-60-69, J84-70-79
404	J90-60-69, J91-60-69, J94-60-69
405	K83-60-69, B18-70-79, C22-70-79, C24-70-79, C25-70-79, K80-70-79, K81-70-79, K82-70-79, K83-70-79, K85-70-79, K86-70-79
406	C21-50-59, C19-60-69, C20-60-69, C21-60-69, K36-60-69, C19-70-79, C20-70-79, C21-70-79, K36-70-79
407	K43-60-69, K56-60-69, K66-60-69, K91-60-69, A08-70-79, K91-70-79
408	K83-60-69, K85-60-69, K86-60-69, B18-70-79, C22-70-79, C24-70-79, C25-70-79, K80-70-79, K81-70-79, K82-70-79, K83-70-79, K85-70-79, K86-70-79
409	K51-10-19, K51-20-29, K51-30-39, K51-40-49, K51-50-59, K51-60-69, K51-70-79
410	I98-60-69, I85-70-79, I98-70-79, K70-70-79, K72-70-79, K74-70-79
411	G31-60-69, F10-70-79, F43-70-79, G31-70-79, K71-70-79
412	K80-60-69, K81-60-69, K82-60-69
413	K83-60-69, B18-70-79, C22-70-79, C24-70-79, K83-70-79

414	K83-60-69, K85-60-69, K86-60-69, B18-70-79, C22-70-79, C24-70-79, C25-70-79, K83-70-79, K85-70-79, K86-70-79
415	L98-40-49, M86-40-49, L97-50-59, L98-50-59, M86-50-59, L97-60-69, L98-60-69, M86-60-69, L97-70-79, L98-70-79, M86-70-79
416	L82-60-69, M18-60-69, M54-70-79, M70-70-79, M79-70-79
417	N26-50-59, L84-60-69, N25-60-69, N26-60-69, N18-70-79, N25-70-79, N26-70-79
418	M05-40-49, M05-50-59, M05-60-69, M05-70-79
419	M17-60-69, M21-60-69, M22-60-69, M17-70-79, M21-70-79, M22-70-79
420	M17-70-79, M21-70-79, M22-70-79
421	M66-50-59, M22-60-69, M23-60-69, M24-60-69, M65-60-69, M67-60-69, M71-60-69, M93-60-69, M94-60-69, M24-70-79
422	M16-50-59, M87-50-59, M16-60-69, M87-60-69
423	M16-50-59, M87-50-59, M16-60-69, M41-60-69, M42-60-69, M43-60-69, M62-60-69, M87-60-69, G54-70-79
424	M41-60-69, M42-60-69, M43-60-69, M50-60-69, M62-60-69, M99-60-69, G54-70-79, G55-70-79, M41-70-79, M42-70-79, M43-70-79, M47-70-79, M48-70-79, M50-70-79, M51-70-79, M53-70-79, M99-70-79
425	M70-50-59, M54-60-69, M70-60-69, B00-70-79, G43-70-79, G83-70-79, M76-70-79, M93-70-79
426	M66-60-69, M75-60-69, M77-60-69
427	N30-50-59, N43-50-59, D29-60-69, D30-60-69, N10-60-69, N39-60-69, N43-60-69, N45-60-69, D30-70-79, H10-70-79, N02-70-79, N39-70-79, N40-70-79, N41-70-79, N42-70-79, N43-70-79, N45-70-79, N99-70-79
428	G35-40-49, N31-40-49, G35-50-59, N31-50-59, G35-60-69, G35-70-79
429	N32-60-69, N35-60-69, N99-60-69, N32-70-79, N35-70-79
430	D29-60-69, N40-60-69, N50-70-79
431	I65-60-69, N39-70-79, N45-70-79, N99-70-79
432	C21-50-59, C21-60-69, C20-70-79, C21-70-79, K36-70-79
433	B18-70-79, C22-70-79, C24-70-79
434	B37-70-79, C34-70-79, C38-70-79, J82-70-79
435	C43-40-49, D22-40-49, C43-50-59, D22-50-59, C43-60-69, D22-60-69, C43-70-79
436	H02-60-69, C44-70-79, D22-70-79, D23-70-79, H02-70-79, L57-70-79, L82-70-79
437	C61-70-79, C64-70-79, D40-70-79, D41-70-79, I89-70-79
438	B37-70-79, C34-70-79, C38-70-79, C77-70-79, C79-70-79, C80-70-79, D38-70-79, D48-70-79, D63-70-79, D70-70-79, J82-70-79, M84-70-79
439	D17-70-79, K40-70-79, K46-70-79
440	H44-50-59, H44-60-69, D31-70-79, H04-70-79, H25-70-79, H34-70-79, H44-70-79, H50-70-79
441	N30-50-59, K63-60-69, C71-70-79, D36-70-79, D43-70-79
442	D50-70-79, D62-70-79, K25-70-79, K26-70-79
443	D75-40-49, D75-50-59, D75-60-69, D64-70-79, D68-70-79, D75-70-79, E21-70-79, E83-70-79
444	E10-70-79, E13-70-79, E16-70-79, H43-70-79, H26-70-79, H33-70-79
445	G91-40-49, I60-40-49, G91-50-59, I60-50-59, G91-60-69, G99-60-69, H00-60-69, H04-60-69, H21-60-69, H59-60-69, E11-70-79, E12-70-79, E53-70-79, E55-70-79, G82-70-79, G91-70-79, N08-70-79
446	I73-30-39, I70-40-49, I73-40-49, I74-40-49, I70-50-59, I73-50-59, I74-50-59, I77-50-59, I74-60-69, I77-60-69
447	G47-20-29, G25-30-39, G47-30-39, G25-40-49, G47-40-49
448	F32-70-79, F33-70-79, F41-70-79, F43-70-79, F45-70-79
449	G56-70-79, M65-70-79, M72-70-79
450	H36-60-69, E10-70-79, E13-70-79, E16-70-79, H43-70-79, H26-70-79, H33-70-79, H35-70-79, H36-70-79
451	I31-50-59, I31-60-69, I20-70-79, I21-70-79, I24-70-79, I31-70-79

452	J90-70-79, J91-70-79, J93-70-79, J94-70-79
453	I61-40-49, I61-50-59, I61-60-69, I61-70-79, I62-70-79
454	G40-70-79, G41-70-79, G81-70-79, I61-70-79, I62-70-79
455	J32-70-79, J33-70-79, J34-70-79
456	L97-70-79, L98-70-79, M86-70-79
457	M16-70-79, M41-70-79, M42-70-79, M43-70-79, M87-70-79
458	M19-70-79, M25-70-79, M75-70-79
459	M99-60-69, G55-70-79, M41-70-79, M42-70-79, M43-70-79, M47-70-79, M48-70-79, M50-70-79, M51-70-79, M53-70-79, M99-70-79
460	N13-50-59, N20-50-59, N23-50-59, N13-60-69, N20-60-69, N21-60-69, N23-60-69, N20-70-79, N21-70-79, N23-70-79
461	N41-40-49, D40-50-59, N41-50-59, N42-50-59, D40-60-69, N41-60-69, C61-70-79, C64-70-79, D40-70-79, N41-70-79, N42-70-79

Supplementary Table 6: All identified trajectories in females. trajectories smaller than 100 members and bigger than 2 members

Community_ID	Members of community
1	I88__0-9, K56__0-9, K59__0-9, K60__0-9, K56-10-19
2	A08__0-9, A38-10-19, B01-10-19, H61-10-19, J38-10-19
3	A38__0-9, B97__0-9, E45__0-9, G47__0-9, H04__0-9, J10__0-9, J35-10-19, J36-10-19, B07-20-29, B34-20-29, G51-20-29, H53-20-29, H72-20-29, H74-20-29, J15-20-29
4	A41__0-9, C91__0-9, C91-10-19
5	B00__0-9, B99__0-9, J05__0-9, J31__0-9, J40__0-9, K52__0-9, B99-10-19, J31-10-19, K08-10-19
6	B27__0-9, J03__0-9, L04__0-9
7	B00__0-9, B08__0-9, B37__0-9, B99__0-9, H10__0-9, H66__0-9, H70__0-9, H92__0-9, J02__0-9, J22__0-9, K21__0-9
8	B96__0-9, N10__0-9, N12__0-9, N10-10-19
9	B97__0-9, J10__0-9, J20__0-9, J10-10-19, J98-10-19, L22-10-19
10	C71__0-9, C71-10-19, C71-20-29
11	D50__0-9, J15__0-9, L22__0-9, J15-10-19
12	D64__0-9, D69__0-9, D70__0-9
13	D68__0-9, H61__0-9, H65__0-9, H74__0-9, J34__0-9, J35__0-9, J39__0-9, H72-10-19
14	E10__0-9, E16__0-9, E87__0-9
15	E45__0-9, E66__0-9, G47__0-9, H65-10-19, H90-10-19, D27-20-29, D39-20-29, F81-20-29, F93-20-29, H65-20-29, H66-20-29, H90-20-29, I88-20-29, J00-20-29, N83-20-29
16	F41__0-9, K02__0-9, K04__0-9, K08__0-9, K10__0-9, K12__0-9, L03__0-9
17	F80__0-9, F82__0-9, F83__0-9, F80-10-19, F82-10-19
18	F84__0-9, G40__0-9, G41__0-9, G81__0-9, G91__0-9, F84-10-19, G41-10-19, G81-10-19, F84-20-29, G81-20-29
19	F89__0-9, F89-10-19, F89-20-29
20	F98__0-9, J06__0-9, J11__0-9, J38__0-9, L50__0-9, J11-10-19, J38-10-19
21	G80__0-9, G82__0-9, M62__0-9, G80-10-19, G82-10-19, M62-10-19, M62-20-29
22	G81__0-9, G81-10-19, G81-20-29
23	H50__0-9, H52__0-9, H53__0-9, H50-10-19, H52-10-19, H50-20-29
24	H66__0-9, H70__0-9, H92__0-9, J22__0-9
25	H73__0-9, J00__0-9, J04__0-9, J12__0-9, J38__0-9, L50__0-9

26	H90__0-9, H91__0-9, H90-10-19, H90-20-29
27	J05__0-9, J31__0-9, K52__0-9, J31-10-19, K08-10-19
28	J30__0-9, J45__0-9, J46__0-9
29	J05__0-9, J31__0-9, J40__0-9, B99-10-19, K08-10-19
30	K90__0-9, K90-10-19, K90-20-29
31	L20__0-9, L20-10-19, L20-20-29, L20-30-39, L20-40-49, L20-50-59
32	N11__0-9, N30__0-9, N35__0-9, N39__0-9, N76__0-9
33	N13__0-9, N28__0-9, N31__0-9
34	000__0-9, A02-10-19, A16-10-19, A38-10-19, A68-10-19, A69-10-19, A87-10-19, B01-10-19, B07-10-19, B09-10-19, B35-10-19, B80-10-19, B95-10-19, C74-10-19, D10-10-19, D57-10-19, D59-10-19, D61-10-19, D70-10-19, D80-10-19, E30-10-19, E45-10-19, F95-10-19, G03-10-19, G04-10-19, G47-10-19, G71-10-19, H04-10-19, H05-10-19, H10-10-19, H35-10-19, H49-10-19, H51-10-19, H60-10-19, H68-10-19, H69-10-19, H70-10-19, H73-10-19, H92-10-19, I78-10-19, J02-10-19, J05-10-19, J09-10-19, J12-10-19, J13-10-19, J16-10-19, J21-10-19, J22-10-19, J39-10-19, J40-10-19, J42-10-19, J46-10-19, J90-10-19, J91-10-19, K00-10-19, K10-10-19, K12-10-19, K13-10-19, K14-10-19, K40-10-19, K42-10-19, K43-10-19, L01-10-19, L03-10-19, L04-10-19, L92-10-19, L95-10-19, M02-10-19, M12-10-19, M13-10-19, M60-10-19, N04-10-19, N11-10-19, N32-10-19, N35-10-19
35	B00-10-19, J31-20-29, J35-20-29, K01-30-39, K07-30-39, K07-40-49
36	B27-10-19, J03-10-19, J36-10-19
37	D27-10-19, N70-10-19, N73-10-19, N83-10-19
38	D69-10-19, D69-20-29, D69-30-39
39	E10__0-9, E16__0-9, E06-10-19, E10-10-19, E14-10-19, E16-10-19, E06-20-29, E16-20-29
40	E66-10-19, E78-10-19, F17-10-19, I10-10-19, K76-10-19, K80-10-19, K76-20-29
41	E73-10-19, E74-10-19, E73-20-29, E74-20-29
42	E84-10-19, E84-20-29, K86-20-29, E84-30-39
43	E87-10-19, F10-10-19, F12-10-19
44	F11-10-19, F13-10-19, F19-10-19
45	F23-10-19, F43-10-19, F70-10-19, F81-10-19, F90-10-19, F93-10-19, F90-20-29
46	F32-10-19, F33-10-19, F41-10-19, F44-10-19, F45-10-19, F93-10-19, L98-10-19
47	F41-10-19, F45-10-19, F41-20-29
48	F60-10-19, F91-10-19, F92-10-19, F92-20-29
49	J96__0-9, G40-10-19, G41-10-19, J96-10-19, G41-20-29, J96-20-29
50	H83-10-19, H91-10-19, H93-10-19
51	I10-10-19, I10-20-29, N18-20-29
52	J20-10-19, J00-20-29, J20-20-29
53	J30__0-9, J45__0-9, J46__0-9, L20__0-9, J30-10-19, J45-10-19, L20-10-19
54	J32-10-19, J34-10-19, M95-10-19
55	K02-10-19, K04-10-19, I88-20-29
56	K21-10-19, K29-10-19, K44-10-19, K92-10-19
57	D50-10-19, K50-10-19, K52-10-19, N92-10-19
58	M21-10-19, M21-20-29, M21-30-39
59	M22-10-19, M23-10-19, M25-10-19, M65-10-19, M67-10-19
60	M41-10-19, M51-10-19, M54-10-19
61	N12-10-19, N30-10-19, N39-10-19, N70-10-19, N73-10-19, N76-10-19
62	N10-10-19, N13-10-19, N20-10-19, N23-10-19
63	A08-10-19, E86-10-19, A08-20-29, A09-20-29, E86-20-29

64	A63-20-29, D06-20-29, N87-20-29
65	B17-20-29, B18-20-29, F10-20-29, F11-20-29, F12-20-29, F13-20-29, F14-20-29, F19-20-29
66	C73-20-29, E04-20-29, E05-20-29, E89-20-29
67	D24-20-29, D48-20-29, N60-20-29
68	D25-20-29, N84-20-29, N85-20-29, N92-20-29
69	D27-20-29, K66-20-29, N73-20-29, N80-20-29, N94-20-29
70	D27-20-29, D39-20-29, K66-20-29, N73-20-29, N80-20-29, N83-20-29, N94-20-29
71	E03__0-9, E03-10-19, D50-20-29, D64-20-29, E03-20-29, E03-30-39, E06-30-39, E06-40-49
72	D68-20-29, D68-30-39, D68-40-49
73	E03__0-9, E03-10-19, E03-20-29, E03-30-39, E06-30-39, E06-40-49
74	E06-10-19, E10-10-19, E14-10-19, E16-10-19, E06-20-29, E10-20-29, E14-20-29, E16-20-29
75	E11-20-29, E66-20-29, G47-20-29, M51-20-29, E65-30-39, L02-30-39
76	F20-20-29, F23-20-29, F25-20-29
77	F31-20-29, F31-30-39, F31-40-49
78	F32-20-29, F42-20-29, I95-20-29, I95-30-39, I95-40-49
79	F61-20-29, F61-30-39, F61-40-49, F61-50-59
80	F70-30-39, F70-40-49, F70-50-59
81	F44-20-29, G40-20-29, F44-30-39, G40-30-39, F44-40-49, J15-40-49, F44-50-59
82	F44-10-19, F45-10-19, F45-20-29, F45-30-39, F45-40-49
83	F50-20-29, M81-20-29, M81-30-39, N91-30-39, M81-40-49
84	G43-10-19, G44-10-19, G43-20-29, G44-20-29
85	G82-20-29, G82-30-39, G82-40-49, G82-50-59
86	I26-20-29, I80-20-29, I82-20-29
87	I95-20-29, I95-30-39, I95-40-49, I95-50-59
88	J01-20-29, J32-20-29, J33-20-29
89	G35-20-29, H46-20-29, F32-30-39, F34-30-39, F48-30-39, G35-30-39, M50-30-39
90	L20-30-39, L20-40-49, L20-50-59
91	K01-20-29, K02-20-29, K04-20-29, K02-30-39
92	K21-10-19, K44-10-19, K25-20-29, K29-20-29, K31-20-29, K58-20-29, K63-20-29
93	D27-20-29, K66-20-29, N70-20-29, N71-20-29, N73-20-29, N76-20-29, N80-20-29, N94-20-29
94	K51-20-29, K51-30-39, K51-40-49, K51-50-59, K51-60-69, K51-70-79
95	B37-10-19, K59-20-29, K59-30-39, K59-40-49
96	I84-20-29, K60-20-29, K61-20-29, K62-20-29
97	K80-20-29, K81-20-29, K82-20-29, K83-20-29, K85-20-29
98	L93-20-29, M32-20-29, M32-30-39
99	M22-20-29, M23-20-29, M24-20-29, M65-20-29, M94-20-29
100	M51-20-29, G55-30-39, M43-30-39, M48-30-39, M51-30-39
101	N10-20-29, N13-20-29, N20-20-29, N23-20-29
102	C53-30-39, D06-30-39, N87-30-39
103	B17-20-29, B18-20-29, F10-20-29, F11-20-29, F12-20-29, F13-20-29, F14-20-29, F19-20-29, B18-30-39, F11-30-39, F12-30-39, F13-30-39, F19-30-39, F12-40-49
104	B24-30-39, B24-40-49, B24-50-59

105	D18-30-39, K25-30-39, K26-30-39, K29-30-39, K31-30-39, K57-30-39, K58-30-39
106	C50-30-39, C77-30-39, C78-30-39, C79-30-39, D05-30-39
107	C71__0-9, C71-10-19, C71-20-29, C71-30-39, D43-30-39
108	C73-30-39, E04-30-39, E05-30-39, E89-30-39
109	C77-30-39, C78-30-39, C79-30-39
110	D21-30-39, D25-30-39, N84-30-39, N85-30-39, N88-30-39, N92-30-39, N93-30-39
111	D23-30-39, I83-30-39, I87-30-39
112	D24-30-39, D48-30-39, N60-30-39
113	D35-30-39, D35-40-49, D35-50-59
114	N80-30-39, N84-30-39, N85-30-39, N88-30-39, N99-30-39, N99-40-49
115	D50-20-29, D64-20-29, E03-20-29, K51-20-29, D50-30-39, D64-30-39, E03-30-39, E06-30-39, K51-30-39
116	D86-30-39, D86-40-49, D86-50-59
117	E10-20-29, E14-20-29, E10-30-39, E11-30-39, E14-30-39
118	E73-30-39, E74-30-39, E73-40-49, E74-40-49
119	E78-30-39, E79-30-39, I21-30-39, I25-30-39, I63-30-39
120	F31-20-29, F10-30-39, F31-30-39, K70-30-39, K71-30-39, K71-40-49
121	F14-30-39, F11-40-49, F11-50-59
122	F17-30-39, J20-30-39, J44-30-39, J43-40-49, J44-40-49, J96-40-49
123	F33-30-39, F40-30-39, F42-30-39, F42-40-49, F42-50-59
124	F42-30-39, F42-40-49, F42-50-59
125	E87-20-29, E87-30-39, F50-30-39, E87-40-49
126	F07-40-49, G40-40-49, G93-40-49, G41-50-59, G93-50-59
127	G43-10-19, G44-10-19, G43-20-29, G44-20-29, G43-30-39, G44-30-39, G43-40-49, G44-40-49, G45-40-49, H81-40-49
128	E66-30-39, G47-30-39, K42-30-39, K43-30-39, E65-40-49
129	M53-30-39, M62-30-39, M99-30-39
130	H33-30-39, H50-30-39, H52-30-39
131	H90-30-39, H93-30-39, H93-40-49
132	I10-30-39, I11-30-39, I20-30-39, I47-30-39, I63-30-39
133	I47-30-39, I47-40-49, I49-40-49, I47-50-59, I49-50-59
134	I73-30-39, I70-40-49, I73-40-49, M34-40-49, M34-50-59
135	I84-30-39, K60-30-39, K61-30-39, K62-30-39
136	J01-30-39, J32-30-39, J33-30-39, J01-40-49, J32-40-49, J33-40-49
137	J03-30-39, J35-30-39, J36-30-39
138	J18-30-39, J90-30-39, J96-30-39
139	J44-30-39, J45-30-39, J43-40-49, J44-40-49, J45-40-49, J96-40-49, J06-50-59
140	J44-30-39, J43-40-49, J44-40-49, J96-40-49
141	K01-30-39, K07-30-39, K07-40-49
142	K21-30-39, K22-30-39, K44-30-39, K21-40-49, K22-40-49, K44-40-49
143	K66-30-39, N70-30-39, N71-30-39, N76-30-39
144	N84-40-49, N85-40-49, N88-40-49, N93-40-49, N95-40-49
145	K44-30-39, D37-40-49, K44-40-49

146	K50-30-39, K56-30-39, K63-30-39, D37-40-49, K56-40-49, K63-40-49, K65-40-49
147	K76-30-39, K76-40-49, N28-40-49, B37-50-59
148	K80-30-39, K81-30-39, K82-30-39, K83-30-39
149	K85-40-49, K86-40-49, K85-50-59, K86-50-59
150	L40-30-39, L40-40-49, L40-50-59
151	L93-30-39, L93-40-49, L93-50-59, L93-60-69, M32-60-69, L93-70-79, M32-70-79
152	M06-30-39, M05-40-49, M06-40-49, M13-50-59
153	M17-30-39, M22-30-39, M23-30-39, M24-30-39, M65-30-39, M67-30-39, M94-30-39
154	M19-30-39, M65-30-39, M75-30-39, M65-40-49, M65-50-59, M72-50-59
155	N10-20-29, N13-20-29, N20-20-29, N23-20-29, N10-30-39, N13-30-39, N20-30-39, N23-30-39, N10-40-49
156	E21-30-39, N18-30-39, N19-30-39, E21-40-49, I15-40-49, N18-40-49, N19-40-49, I15-50-59, N17-50-59, N19-50-59
157	N80-30-39, N84-30-39, N85-30-39, N88-30-39, N92-30-39, N93-30-39, N99-30-39, N99-40-49
158	A46-40-49, A46-50-59, B35-50-59
159	B16-40-49, B17-50-59, B18-50-59, B17-60-69, B18-60-69, K73-60-69, B18-70-79
160	G83-50-59, H10-50-59, M54-60-69, M70-60-69, M76-60-69, J04-70-79, J31-70-79, M76-70-79
161	C18-40-49, C18-50-59, C19-50-59
162	C43-40-49, C43-50-59, C43-60-69, C43-70-79
163	C48-40-49, C56-40-49, D39-40-49
164	C50-40-49, D05-40-49, D17-40-49, D24-40-49, D48-40-49, N60-40-49
165	C73-40-49, E89-40-49, C73-50-59, E89-50-59
166	C82-40-49, C83-40-49, C85-40-49
167	C53-30-39, D06-30-39, C53-40-49, C54-40-49, C55-40-49, D06-40-49, N87-40-49
168	D12-40-49, I84-40-49, K64-50-59
169	D17-40-49, D24-40-49, D48-40-49, N60-40-49
170	D21-40-49, D25-40-49, D27-40-49, D62-40-49, K36-40-49
171	C43-40-49, D22-40-49, C43-50-59, D22-50-59
172	I83-40-49, I87-40-49, I83-50-59, I87-50-59
173	D26-40-49, N84-40-49, N85-40-49, N88-40-49, N92-40-49, N93-40-49, N95-40-49
174	D34-40-49, D44-40-49, E04-40-49, E55-40-49, E83-40-49, J38-40-49
175	K51-20-29, K51-30-39, D50-40-49, E61-40-49, K51-40-49, E61-50-59, K51-50-59, K51-60-69, K51-70-79
176	D53-40-49, F10-40-49, G62-40-49, I85-40-49, K71-40-49, K74-40-49, D53-50-59, F12-50-59, G62-50-59, K71-50-59
177	D69-10-19, D69-20-29, D69-30-39, D69-40-49, D70-40-49
178	H25-40-49, H26-40-49, H33-40-49, H35-40-49, H33-50-59
179	E13-40-49, E11-50-59, E12-50-59, E13-50-59, G63-50-59, L03-50-59, G63-60-69, M86-60-69, N08-60-69
180	E65-40-49, L90-40-49, N62-40-49
181	E66-40-49, I89-40-49, K42-40-49, K43-40-49, M16-40-49, E65-50-59, K42-50-59, K43-50-59
182	E73-40-49, E74-40-49, E73-50-59, E74-50-59
183	M42-40-49, M43-40-49, M48-40-49
184	F48-40-49, G25-40-49, G47-40-49, F48-50-59, G25-50-59, G47-50-59
185	F17-40-49, G45-40-49, J38-40-49, J40-40-49, J43-40-49, J98-40-49
186	F20-20-29, F23-20-29, F25-20-29, F20-30-39, F23-30-39, F25-30-39, F20-40-49, F25-40-49

187	F20-50-59, F25-50-59, F20-60-69, F25-60-69, F20-70-79, F25-70-79
188	F79-50-59, F79-60-69, F79-70-79
189	N31-30-39, B00-40-49, G35-40-49, N31-40-49, B00-50-59, G35-50-59, N31-50-59, N31-60-69
190	G25-40-49, G47-40-49, G25-50-59, G47-50-59
191	M51-20-29, G55-30-39, M43-30-39, M48-30-39, M51-30-39, G55-40-49, G57-40-49, M51-40-49, M96-40-49
192	G55-40-49, M42-40-49, M43-40-49, M48-40-49, M50-40-49
193	M51-20-29, G55-30-39, M43-30-39, M48-30-39, M51-30-39, G54-40-49, G55-40-49, G57-40-49, M40-40-49, M51-40-49, M54-40-49, M96-40-49, G54-50-59, M96-50-59, M96-60-69
194	G58-40-49, M40-50-59, M54-50-59, M70-50-59, M76-50-59, M80-50-59, M93-50-59, D16-60-69, D36-60-69, F54-60-69, G83-60-69, H65-60-69, K04-60-69, M40-60-69
195	G70-40-49, G70-50-59, G70-60-69
196	G80-40-49, G80-50-59, G80-60-69
197	G91-40-49, I60-40-49, I61-40-49
198	H02-40-49, H52-40-49, H53-40-49
199	H80-40-49, H90-40-49, H80-50-59
200	H81-40-49, H91-40-49, H93-40-49, H81-50-59, H90-50-59, H91-50-59, H93-50-59, H81-60-69, H90-60-69, H91-60-69, H93-60-69, H91-70-79, H93-70-79
201	M35-30-39, I10-40-49, I11-40-49, I15-40-49, I27-40-49, I35-40-49, I44-40-49, I51-40-49, I74-40-49, I77-40-49, J15-40-49, L30-40-49, M13-40-49, M35-40-49
202	I21-40-49, I25-40-49, I42-40-49, I50-40-49, I20-50-59, I21-50-59, I24-50-59
203	I20-40-49, I21-40-49, I25-40-49, I42-40-49, I50-40-49, I20-50-59, I21-50-59, I24-50-59, I25-50-59, I42-50-59, I44-50-59, I50-50-59, I51-50-59
204	I25-40-49, I42-40-49, I50-40-49, I42-50-59, I44-50-59, I50-50-59, I51-50-59, I42-60-69, I44-60-69, I44-70-79
205	I26-40-49, I80-40-49, I82-40-49
206	I42-40-49, I50-40-49, I42-50-59, I44-50-59, I44-60-69, I44-70-79
207	I45-40-49, I45-50-59, I45-60-69, I45-70-79
208	E78-40-49, E79-40-49, G45-40-49, I20-40-49, I21-40-49, I44-40-49, I51-40-49, M41-40-49, E79-50-59, M41-50-59
209	I63-40-49, I64-40-49, I65-40-49, G46-50-59, I63-50-59, I64-50-59, I65-50-59
210	I67-40-49, I72-40-49, I67-50-59
211	I69-40-49, G91-50-59, I60-50-59, I61-50-59, I69-50-59, G91-60-69, I60-60-69
212	I85-40-49, K74-40-49, I85-50-59, K74-50-59
213	A41-40-49, J18-40-49, J90-40-49, J96-40-49, N17-40-49
214	J34-40-49, J35-40-49, M95-40-49
215	K51-40-49, K51-50-59, K51-60-69, K51-70-79
216	K31-40-49, K31-50-59, K31-60-69
217	F60-50-59, F60-60-69, F61-60-69, F60-70-79
218	K42-40-49, K43-40-49, K42-50-59, K43-50-59
219	D21-30-39, D25-30-39, D27-30-39, K66-30-39, N70-30-39, N71-30-39, N73-30-39, N80-30-39, N83-30-39, N84-30-39, N85-30-39, N88-30-39, N92-30-39, N93-30-39, N94-30-39, N99-30-39, K66-40-49, N70-40-49, N73-40-49
220	K58-40-49, K58-50-59, K58-60-69, K58-70-79
221	K60-40-49, K61-40-49, K60-50-59, K61-50-59
222	K80-40-49, K81-40-49, K82-40-49, K83-40-49
223	K83-40-49, C25-50-59, K83-50-59, K83-60-69, K83-70-79
224	L02-40-49, L02-50-59, L02-60-69

225	D21-50-59, D25-50-59, D27-50-59, D62-50-59, N70-50-59, N73-50-59, N83-50-59, A69-60-69, D21-60-69, D27-60-69, G58-60-69, K35-60-69, N35-60-69, N70-60-69, N73-60-69, N83-60-69
226	C79-60-69, D61-60-69, D70-60-69, M84-60-69
227	M16-40-49, M16-50-59, M87-50-59
228	M17-40-49, M21-40-49, M22-40-49, M23-40-49, M71-40-49, M93-40-49, M94-40-49, M94-50-59
229	M20-30-39, M77-30-39, M20-40-49, M21-40-49, G57-50-59, M20-50-59
230	M22-40-49, M23-40-49, M71-40-49, M93-40-49, M94-40-49, M22-50-59, M23-50-59, M71-50-59, M93-50-59, M94-50-59
231	M23-40-49, M71-40-49, M93-40-49
232	M65-30-39, M24-40-49, M65-40-49, M67-40-49, M24-50-59, M65-50-59, M67-50-59, M72-50-59
233	M34-40-49, M34-50-59, M34-60-69, M34-70-79
234	M45-40-49, M45-50-59, M45-60-69
235	G54-40-49, G55-40-49, M41-40-49, M42-40-49, M43-40-49, M47-40-49, M48-40-49, M50-40-49, M53-40-49, M62-40-49, M99-40-49, E55-50-59, M41-50-59, M42-50-59, M43-50-59, M47-50-59, M48-50-59, M62-50-59, M99-50-59
236	N04-40-49, A69-50-59, B99-50-59, E07-50-59, I10-50-59, I31-50-59, I46-50-59, I71-50-59, J03-50-59, J22-50-59, J30-50-59, J41-50-59, J42-50-59, J81-50-59, J84-50-59, K55-50-59, K75-50-59, L27-50-59, L50-50-59, L98-50-59, M86-50-59, N30-50-59, E26-60-69, N03-60-69
237	N35-40-49, N39-40-49, N76-40-49, N81-40-49, N10-50-59, N30-50-59
238	N13-40-49, N20-40-49, N23-40-49, N13-50-59, N20-50-59, N23-50-59
239	E21-30-39, E21-40-49, N18-40-49, N19-40-49, E21-50-59, N17-50-59, N19-50-59
240	K66-30-39, N70-30-39, N71-30-39, N73-30-39, N76-30-39, K66-40-49, N70-40-49, N73-40-49
241	N83-40-49, N99-40-49, N76-50-59, N99-50-59
242	N97-40-49, D13-50-59, K80-50-59, K81-50-59, K82-50-59, N97-50-59, K91-60-69
243	A04-50-59, K80-60-69, K81-60-69, K82-60-69, D72-70-79
244	A08-50-59, J31-50-59, M46-50-59, A49-60-69, B96-60-69, K29-60-69, K55-60-69, D22-70-79, K20-70-79, L28-70-79, N60-70-79
245	B02-50-59, D13-50-59, E78-50-59, E83-50-59, G51-50-59, G58-50-59, I07-50-59, I15-50-59, I71-50-59, I72-50-59, I72-60-69
246	B17-50-59, B17-60-69, B18-60-69, B18-70-79
247	C16-50-59, C16-60-69, C16-70-79
248	C18-40-49, C18-50-59, C19-50-59, C20-50-59, C21-50-59
249	C22-50-59, C38-50-59, C49-50-59, C78-50-59, D61-50-59, J91-50-59
250	C34-40-49, D38-40-49, C34-50-59, D38-50-59, J82-50-59
251	C48-40-49, C56-40-49, D39-40-49, C48-50-59, C56-50-59, C57-50-59
252	C50-50-59, D05-50-59, D24-50-59, N60-50-59, N62-50-59, N64-50-59, N60-60-69
253	C53-70-79, C54-70-79, C55-70-79
254	C53-50-59, C54-50-59, C55-50-59, C53-60-69, C54-60-69, C55-60-69, C53-70-79
255	C67-50-59, C67-60-69, C67-70-79, C68-70-79
256	C71-40-49, D43-40-49, C71-50-59, D43-50-59
257	C82-50-59, C82-60-69, C82-70-79
258	C83-50-59, C85-50-59, C85-60-69
259	C90-50-59, C90-60-69, C90-70-79
260	C91-50-59, C91-60-69, C91-70-79
261	C92-50-59, C92-60-69, C92-70-79
262	D13-50-59, K80-50-59, K81-50-59, K82-50-59, K91-60-69

263	D17-50-59, D17-60-69, D17-70-79
264	D32-50-59, D32-60-69, D32-70-79
265	M47-40-49, D34-50-59, D44-50-59, E04-50-59, E21-50-59, E55-50-59, E83-50-59, J38-50-59, E07-60-69
266	D47-50-59, D47-60-69, D47-70-79
267	D17-50-59, D48-50-59, D17-60-69, D48-60-69, D17-70-79
268	D50-50-59, D62-60-69, E61-60-69
269	A04-70-79, A09-70-79, A41-70-79, A46-70-79, B35-70-79, K65-70-79
270	C50-60-69, D05-60-69, D24-60-69, N64-60-69, I97-70-79
271	D68-50-59, D68-60-69, D68-70-79
272	D75-50-59, J37-50-59, J44-60-69, J93-60-69, B96-70-79, J93-70-79
273	E03-50-59, E10-50-59, E13-60-69, E16-60-69, E13-70-79
274	E16-50-59, E10-60-69, E10-70-79, E13-70-79, E16-70-79, G63-70-79
275	I99-50-59, A04-60-69, E78-60-69, E83-60-69, G46-60-69, G51-60-69, G58-60-69, I08-60-69, J22-60-69, M10-60-69, M85-60-69, E83-70-79, F50-70-79, N04-70-79, N05-70-79, N62-70-79, N87-70-79
276	M16-40-49, I89-50-59, M16-50-59, M87-50-59, I89-60-69, I89-70-79
277	F06-50-59, F07-50-59, F06-60-69, F07-60-69
278	F10-50-59, F19-50-59, K71-50-59, D52-60-69, E86-60-69, F19-60-69, G93-60-69, I46-60-69, K26-60-69, M80-60-69, G93-70-79, K26-70-79, M80-70-79
279	F06-40-49, F48-40-49, F32-50-59, F40-50-59, F44-50-59, F48-50-59, D16-60-69, F11-60-69, F22-60-69, F23-60-69, F44-60-69, F51-60-69, F55-60-69, J00-60-69, J37-60-69, L28-60-69, N89-60-69
280	F61-20-29, F61-30-39, F61-40-49, F33-50-59, F50-50-59, F61-50-59, F50-60-69
281	G43-30-39, G44-30-39, G43-40-49, G44-40-49, G45-40-49, H81-40-49, G43-50-59, G44-50-59, H81-50-59
282	G35-60-69, G35-70-79, N31-70-79
283	G20-50-59, G20-60-69, G21-70-79
284	F07-40-49, G93-40-49, G40-50-59, G93-50-59, G41-60-69
285	G45-50-59, G45-60-69, G45-70-79
286	G50-50-59, G50-60-69, G50-70-79
287	G56-30-39, G56-40-49, G56-50-59, M18-50-59, M72-50-59
288	G81-50-59, G81-60-69, G81-70-79
289	G91-50-59, I60-50-59, I61-50-59, G91-60-69, I60-60-69
290	H25-40-49, H26-40-49, H33-40-49, H35-40-49, H25-50-59, H26-50-59, H33-50-59
291	H02-40-49, H52-40-49, H53-40-49, H02-50-59, H52-50-59, H53-50-59
292	H90-50-59, H91-50-59, H93-50-59, H81-60-69, H90-60-69, H91-60-69, H93-60-69, H91-70-79, H93-70-79
293	I05-50-59, I05-60-69, I05-70-79
294	I07-40-49, I34-40-49, I07-50-59, I34-50-59, I35-50-59, I36-50-59
295	I11-50-59, I11-60-69, I11-70-79
296	I12-50-59, N18-60-69, N25-60-69, N26-60-69, N18-70-79, N25-70-79, N26-70-79
297	I26-40-49, I80-40-49, I82-40-49, I26-50-59, I80-50-59
298	I35-50-59, I35-60-69, I71-60-69, I35-70-79, I71-70-79
299	I44-50-59, I44-60-69, I44-70-79
300	I70-50-59, I77-50-59, I77-60-69
301	I82-50-59, I82-60-69, I82-70-79
302	I89-50-59, I89-60-69, I89-70-79

303	J01-50-59, J32-50-59, J33-50-59, J01-60-69, J32-60-69, J33-60-69, J34-60-69
304	J98-50-59, J98-60-69, J98-70-79
305	K22-50-59, K22-60-69, K22-70-79
306	J38-50-59, J38-60-69, J38-70-79
307	J43-50-59, J96-50-59, J43-60-69, J96-60-69, J15-70-79, J43-70-79, J96-70-79
308	J84-50-59, J84-60-69, J84-70-79
309	K21-50-59, K22-50-59, K26-50-59, K22-60-69, K22-70-79
310	K42-60-69, K43-60-69, K66-60-69, K42-70-79, K43-70-79, K66-70-79
311	C48-50-59, K56-50-59, K65-50-59, K66-50-59
312	K59-30-39, E65-40-49, K59-40-49, L90-40-49, N62-40-49, K59-50-59, L90-50-59
313	K63-50-59, K65-50-59, D13-60-69
314	K90-50-59, K90-60-69, K90-70-79
315	M16-40-49, E66-50-59, I89-50-59, L30-50-59, M16-50-59, M87-50-59, E65-60-69, I89-60-69, K45-60-69, L27-60-69, N62-60-69, I89-70-79
316	K59-60-69, L90-60-69, L90-70-79
317	L93-50-59, L93-60-69, M32-60-69, L93-70-79, M32-70-79
318	L97-50-59, L97-60-69, L97-70-79, L98-70-79
319	M17-40-49, M21-40-49, M22-40-49, M23-40-49, M71-40-49, M93-40-49, M94-40-49, M17-50-59, M22-50-59, M23-50-59, M71-50-59, M93-50-59, M94-50-59
320	M31-50-59, M31-60-69, M31-70-79
321	M32-50-59, M32-60-69, M32-70-79
322	M35-30-39, M35-40-49, M35-50-59, M35-60-69, M35-70-79
323	M81-30-39, M81-40-49, M81-50-59, M85-60-69
324	N00-50-59, D30-60-69, D46-60-69, E53-60-69, F05-60-69, G31-60-69, H49-60-69, I10-60-69, I12-60-69, I13-60-69, I62-60-69, I97-60-69, I99-60-69, J02-60-69, J04-60-69, J22-60-69, J30-60-69, J41-60-69, J42-60-69, J69-60-69, J94-60-69, J95-60-69, K20-60-69, K73-60-69, K75-60-69, L29-60-69, M10-60-69, M40-60-69, M46-60-69, N10-60-69, N31-60-69, D26-70-79, E24-70-79, L73-70-79
325	E10-40-49, E13-40-49, E14-40-49, E10-50-59, E11-50-59, E12-50-59, E13-50-59, E14-50-59, H43-50-59, G63-50-59, L03-50-59, N08-50-59, A04-60-69, E11-60-69, E12-60-69, E13-60-69, E16-60-69, F01-60-69, H43-60-69, G51-60-69, G63-60-69, I79-60-69, L03-60-69, L89-60-69, L98-60-69, M86-60-69, N08-60-69
326	N28-50-59, N28-60-69, N28-70-79
327	N35-50-59, N39-50-59, N76-50-59, G83-60-69, N30-60-69
328	N70-50-59, N73-50-59, N73-60-69, N83-60-69
329	N80-50-59, N81-50-59, N84-50-59, N85-50-59, N88-50-59, N95-50-59, N80-60-69
330	N81-50-59, N81-60-69, N99-60-69
331	N70-50-59, N73-50-59, N83-50-59, D27-60-69, G58-60-69, N73-60-69, N83-60-69
332	N84-50-59, N88-50-59, N95-50-59
333	C53-50-59, C54-50-59, C55-50-59, N95-50-59
334	N39-60-69, D72-70-79, N32-70-79, N35-70-79
335	A18-60-69, A40-60-69, B36-60-69, B90-60-69, C15-60-69, C23-60-69, C32-60-69, C51-60-69, C52-60-69, C69-60-69, C76-60-69, D03-60-69, D04-60-69, D30-60-69, D31-60-69, D46-60-69, D59-60-69, D89-60-69, E20-60-69, E41-60-69, E46-60-69, E53-60-69, E72-60-69, F05-60-69, F09-60-69, G12-60-69, G24-60-69, G31-60-69, G90-60-69, H01-60-69, H17-60-69, H21-60-69, H31-60-69, H49-60-69, H57-60-69, H82-60-69, I06-60-69, I22-60-69, I33-60-69, I81-60-69, I98-60-69, J11-60-69, J12-60-69, J21-60-69, K06-60-69, K14-60-69, K28-60-69, K41-60-69, K46-60-69, L08-60-69, L24-60-69, L53-60-69, L82-60-69, L84-60-69, L85-60-69, M00-60-69, M11-60-69, M82-60-69, N32-60-69, N36-60-69, 000-70-79
336	A46-40-49, A46-50-59, B35-50-59, A46-60-69, B35-60-69
337	M51-50-59, M96-50-59, A69-60-69, G54-60-69, G57-60-69, M96-60-69, G57-70-79

338	D12-40-49, D12-50-59, C18-60-69, C19-60-69, D12-60-69
339	C18-40-49, C18-50-59, C19-50-59, C20-50-59, C21-50-59, C20-60-69, C21-60-69
340	C34-60-69, D38-60-69, J82-60-69, C34-70-79, D38-70-79
341	C38-60-69, J90-60-69, J91-60-69
342	C43-60-69, C44-60-69, L57-60-69, C43-70-79, C44-70-79, L57-70-79
343	C48-60-69, C48-70-79, C56-70-79, C57-70-79, D39-70-79
344	C38-60-69, C78-60-69, D61-60-69, D70-60-69, J90-60-69, J91-60-69, C80-70-79, D70-70-79
345	C53-60-69, C54-60-69, C55-60-69, C53-70-79, C54-70-79, C55-70-79
346	C48-60-69, C56-60-69, C57-60-69, D39-60-69, C48-70-79, C56-70-79, C57-70-79, D39-70-79
347	C64-60-69, D41-60-69, C64-70-79, D41-70-79
348	C71-60-69, D43-60-69, C71-70-79, D43-70-79
349	C73-40-49, E89-40-49, C73-50-59, E89-50-59, C73-60-69, E89-60-69, C73-70-79
350	C38-60-69, C77-60-69, C78-60-69, C79-60-69, C80-60-69, D61-60-69, D63-60-69, D70-60-69, J90-60-69, J91-60-69, M84-60-69, C23-70-79, C77-70-79, C78-70-79, C79-70-79, C80-70-79, D48-70-79, D63-70-79, D70-70-79, M84-70-79
351	D63-50-59, D69-50-59, D70-50-59, D61-60-69, D63-60-69, D69-60-69, D70-60-69, D46-70-79, D63-70-79, D69-70-79
352	K50-60-69, A02-70-79, B17-70-79, B34-70-79, D52-70-79, D53-70-79, E07-70-79, E46-70-79, E72-70-79, F09-70-79, G60-70-79, G70-70-79, G83-70-79, G90-70-79, H49-70-79, H61-70-79, I10-70-79, I30-70-79, I99-70-79, J02-70-79, J04-70-79, J11-70-79, J30-70-79, J41-70-79, J94-70-79, K20-70-79, K27-70-79, K28-70-79, K30-70-79, K46-70-79, K50-70-79, K73-70-79, K75-70-79, L08-70-79, L28-70-79, L29-70-79, L50-70-79, L82-70-79, L85-70-79, M00-70-79, M34-70-79, M40-70-79, M46-70-79, N12-70-79
353	D18-40-49, D18-50-59, D18-60-69, D18-70-79
354	L72-40-49, N92-50-59, D21-60-69, D23-60-69, D72-60-69, E65-60-69, K35-60-69, K91-60-69, L50-60-69, L72-60-69, M84-60-69, N35-60-69, N87-60-69, N94-60-69
355	C53-60-69, C54-60-69, C55-60-69, D25-60-69, C53-70-79, C54-70-79, C55-70-79, K81-70-79
356	D35-30-39, D35-40-49, D35-50-59, D35-60-69, D35-70-79
357	D38-60-69, C34-70-79, D38-70-79
358	L25-60-69, D53-70-79, N39-70-79, N76-70-79
359	D61-60-69, D69-60-69, D70-60-69, D46-70-79, D61-70-79, D69-70-79
360	E21-50-59, E21-60-69, E21-70-79
361	D69-60-69, D46-70-79, D61-70-79, D69-70-79
362	D86-30-39, D86-40-49, D86-50-59, D86-60-69, D86-70-79
363	E13-60-69, E10-70-79, E13-70-79, E16-70-79
364	E14-60-69, E12-70-79, E14-70-79, E16-70-79, I79-70-79, L03-70-79, M86-70-79, N08-70-79
365	D34-50-59, E01-50-59, J38-50-59, E01-60-69, E04-60-69, E55-60-69, J38-60-69, J38-70-79, L72-70-79, N35-70-79
366	E73-60-69, E74-60-69, E73-70-79, E74-70-79
367	F00-60-69, F01-60-69, F03-60-69, G30-60-69, F00-70-79, F05-70-79, G30-70-79
368	F01-60-69, F03-60-69, G30-60-69
369	H43-60-69, H33-60-69, H43-70-79, H18-70-79, H26-70-79, H33-70-79
370	F17-60-69, A04-70-79, A09-70-79, A41-70-79, A46-70-79, B35-70-79, B37-70-79, I24-70-79, I77-70-79, J41-70-79, K65-70-79, L30-70-79
371	F31-20-29, F31-30-39, F31-40-49, F31-50-59, F31-60-69, F31-70-79
372	F32-60-69, F48-60-69, F07-70-79, F19-70-79, F22-70-79, F40-70-79, F44-70-79, F48-70-79, F51-70-79, G58-70-79, L29-70-79, L82-70-79
373	H90-50-59, H91-50-59, H93-50-59, H90-60-69, H91-60-69, H93-60-69, H91-70-79, H93-70-79

374	H10-50-59, F13-60-69, F33-60-69, F41-60-69, F43-60-69, F45-60-69, F13-70-79, F33-70-79, F41-70-79, F43-70-79, F45-70-79, K59-70-79
375	K20-40-49, K51-40-49, A49-50-59, B96-50-59, K20-50-59, K29-50-59, K51-50-59, H65-60-69, J37-60-69, K51-60-69, N89-60-69, N93-60-69, K51-70-79
376	G21-60-69, F02-70-79, G20-70-79
377	G81-50-59, G40-60-69, G41-60-69, G81-60-69, G40-70-79, G41-70-79, G81-70-79
378	I72-50-59, I67-60-69, I72-60-69, I67-70-79
379	M96-50-59, G55-60-69, M51-60-69, M96-60-69
380	G56-70-79, M15-70-79, M18-70-79
381	G82-30-39, G82-40-49, G82-50-59, G82-60-69, G82-70-79
382	G93-60-69, I46-60-69, G93-70-79
383	G95-60-69, M48-70-79, M50-70-79
384	H10-60-69, H11-60-69, H18-60-69, H25-60-69, H27-60-69, H34-60-69, H44-60-69, H34-70-79
385	H40-40-49, H25-50-59, H26-50-59, H40-50-59, H26-60-69
386	H34-60-69, H54-60-69, D31-70-79, H43-70-79, H04-70-79, H11-70-79, H16-70-79, H17-70-79, H18-70-79, H25-70-79, H26-70-79, H27-70-79, H33-70-79, H34-70-79, H35-70-79, H44-70-79, H54-70-79
387	H40-60-69, H47-60-69, H40-70-79, H47-70-79
388	H50-60-69, H52-60-69, H53-60-69, H50-70-79, H52-70-79, H53-70-79
389	H90-50-59, H91-50-59, H93-50-59, H90-60-69
390	I26-40-49, I80-40-49, I82-40-49, I26-50-59, I80-50-59, I82-50-59, I26-60-69, I80-60-69, I82-60-69, I26-70-79, I82-70-79
391	I07-40-49, I34-40-49, I07-50-59, I34-50-59, I35-50-59, I36-50-59, I07-60-69, I34-60-69, I35-60-69, I36-60-69
392	I49-40-49, I49-50-59, I49-60-69, I49-70-79
393	G46-60-69, I63-60-69, I66-60-69, I63-70-79
394	I73-30-39, I70-40-49, I73-40-49, M34-40-49, I73-50-59, I74-50-59, M34-50-59, I74-60-69
395	I84-60-69, K60-60-69, K60-70-79, K64-70-79
396	I83-40-49, I87-40-49, I83-50-59, I87-50-59, I87-60-69, I87-70-79
397	F61-20-29, F61-30-39, F61-40-49, F33-50-59, F43-50-59, F50-50-59, F60-50-59, F61-50-59, B37-60-69, F40-60-69, F50-60-69, F60-60-69, F61-60-69, L30-60-69, F60-70-79
398	J32-60-69, J33-60-69, J34-60-69
399	J20-50-59, J20-60-69, J20-70-79
400	K42-40-49, K43-40-49, K42-50-59, K43-50-59, K42-60-69, K43-60-69, K42-70-79, K43-70-79
401	J98-50-59, J45-60-69, J98-60-69, J45-70-79, J96-70-79, J98-70-79
402	K59-30-39, E65-40-49, K59-40-49, L90-40-49, N62-40-49, K59-50-59, L90-50-59, K59-60-69, L90-60-69, L90-70-79
403	I85-60-69, K70-60-69, K72-60-69, K74-60-69, I85-70-79, K70-70-79, K74-70-79
404	K85-60-69, K86-60-69, K85-70-79, K86-70-79
405	L23-40-49, L23-50-59, L23-60-69, L23-70-79
406	G50-50-59, G50-60-69, E78-70-79, G43-70-79, G50-70-79, G51-70-79, G55-70-79, G57-70-79, G58-70-79, G91-70-79, G95-70-79, H10-70-79, H90-70-79, I06-70-79, I60-70-79, I99-70-79, M40-70-79
407	M06-30-39, M05-40-49, M06-40-49, M05-50-59, M06-50-59, M13-50-59, M05-60-69, M06-60-69, M13-60-69, M05-70-79, M06-70-79, M13-70-79
408	M17-70-79, M22-70-79, M23-70-79, M24-70-79, M65-70-79, M67-70-79, M71-70-79, M87-70-79, M94-70-79
409	G57-60-69, M20-60-69, M77-60-69, G57-70-79
410	M17-60-69, M21-60-69, M22-60-69, M23-60-69, M24-60-69, M67-60-69, M71-60-69, M94-60-69, M00-70-79, M17-70-79, M21-70-79, M22-70-79

411	G95-60-69, M41-60-69, M42-60-69, M43-60-69, M48-60-69, M50-60-69, M62-60-69, M99-60-69, G54-70-79, G95-70-79, M41-70-79, M42-70-79, M43-70-79, M47-70-79, M48-70-79, M50-70-79, M51-70-79, M53-70-79, M54-70-79, M62-70-79, M70-70-79, M93-70-79, M96-70-79, M99-70-79
412	G95-60-69, M48-60-69, M50-60-69, G95-70-79, M48-70-79, M50-70-79
413	N13-60-69, N20-60-69, N23-60-69, N13-70-79, N20-70-79, N23-70-79
414	N26-40-49, N26-50-59, N26-60-69, N26-70-79
415	C50-70-79, D05-70-79, D24-70-79, L90-70-79, N64-70-79
416	D17-50-59, N84-60-69, N88-60-69
417	C48-60-69, C18-70-79, C19-70-79, C20-70-79, C21-70-79, C48-70-79, D12-70-79
418	C19-70-79, C20-70-79, C21-70-79
419	C77-60-69, C80-60-69, D63-60-69, D70-60-69, C23-70-79, C77-70-79, C78-70-79, C79-70-79, C80-70-79, D48-70-79, D63-70-79, D70-70-79, M84-70-79
420	C43-70-79, C44-70-79, L57-70-79
421	C83-50-59, C85-50-59, C83-60-69, C85-60-69, C83-70-79, C85-70-79
422	D25-70-79, N81-70-79, N99-70-79
423	H34-60-69, D31-70-79, H04-70-79, H11-70-79, H16-70-79, H17-70-79, H25-70-79, H27-70-79, H34-70-79, H44-70-79
424	D50-70-79, D62-70-79, E61-70-79
425	F31-60-69, A49-70-79, E11-70-79, E12-70-79, E53-70-79, F31-70-79, G51-70-79, I12-70-79, I13-70-79, I62-70-79, K71-70-79, K72-70-79, L02-70-79, N08-70-79, N10-70-79
426	E79-40-49, E79-50-59, E79-60-69, E79-70-79, M10-70-79
427	E88-40-49, E88-50-59, E88-60-69, E88-70-79
428	F03-70-79, F13-70-79, F20-70-79, F22-70-79, G31-70-79
429	H43-70-79, H18-70-79, H26-70-79, H33-70-79
430	G40-70-79, G41-70-79, G81-70-79
431	M96-50-59, G55-60-69, G95-60-69, M48-60-69, M50-60-69, M51-60-69, M96-60-69, G54-70-79, G55-70-79, G95-70-79, M47-70-79, M48-70-79, M50-70-79, M51-70-79, M54-70-79, M70-70-79, M93-70-79, M96-70-79
432	H02-60-69, H02-70-79, H18-70-79, H26-70-79
433	I21-70-79, I24-70-79, I46-70-79
434	A49-70-79, I25-70-79, I31-70-79, I48-70-79, I50-70-79, I62-70-79, I72-70-79, I97-70-79, J06-70-79, J22-70-79, J42-70-79, J81-70-79, J90-70-79, J91-70-79, J95-70-79, L27-70-79, M10-70-79
435	I07-40-49, I34-40-49, I07-50-59, I34-50-59, I35-50-59, I36-50-59, I07-60-69, I34-60-69, I35-60-69, I36-60-69, I07-70-79, I34-70-79, I36-70-79
436	I50-60-69, J81-60-69, I50-70-79, J22-70-79, J90-70-79, J91-70-79, J95-70-79
437	J44-70-79, J47-70-79, J93-70-79
438	K85-60-69, K86-60-69, C23-70-79, K80-70-79, K81-70-79, K82-70-79, K85-70-79, K86-70-79
439	L40-30-39, L40-40-49, L40-50-59, L40-60-69, L40-70-79
440	G55-60-69, G95-60-69, M41-60-69, M43-60-69, M48-60-69, M50-60-69, M51-60-69, M53-60-69, M62-60-69, M99-60-69, G54-70-79, G55-70-79, G95-70-79, M16-70-79, M25-70-79, M41-70-79, M42-70-79, M43-70-79, M47-70-79, M48-70-79, M50-70-79, M51-70-79, M53-70-79, M54-70-79, M62-70-79, M70-70-79, M87-70-79, M93-70-79, M96-70-79, M99-70-79
441	M19-70-79, M20-70-79, M77-70-79
442	G95-60-69, M47-70-79, M48-70-79, M50-70-79, M93-70-79
443	G35-50-59, N31-50-59, G35-60-69, N31-60-69, G35-70-79, N31-70-79
444	D25-70-79, D27-70-79, N81-70-79, N83-70-79, N84-70-79, N85-70-79, N99-70-79

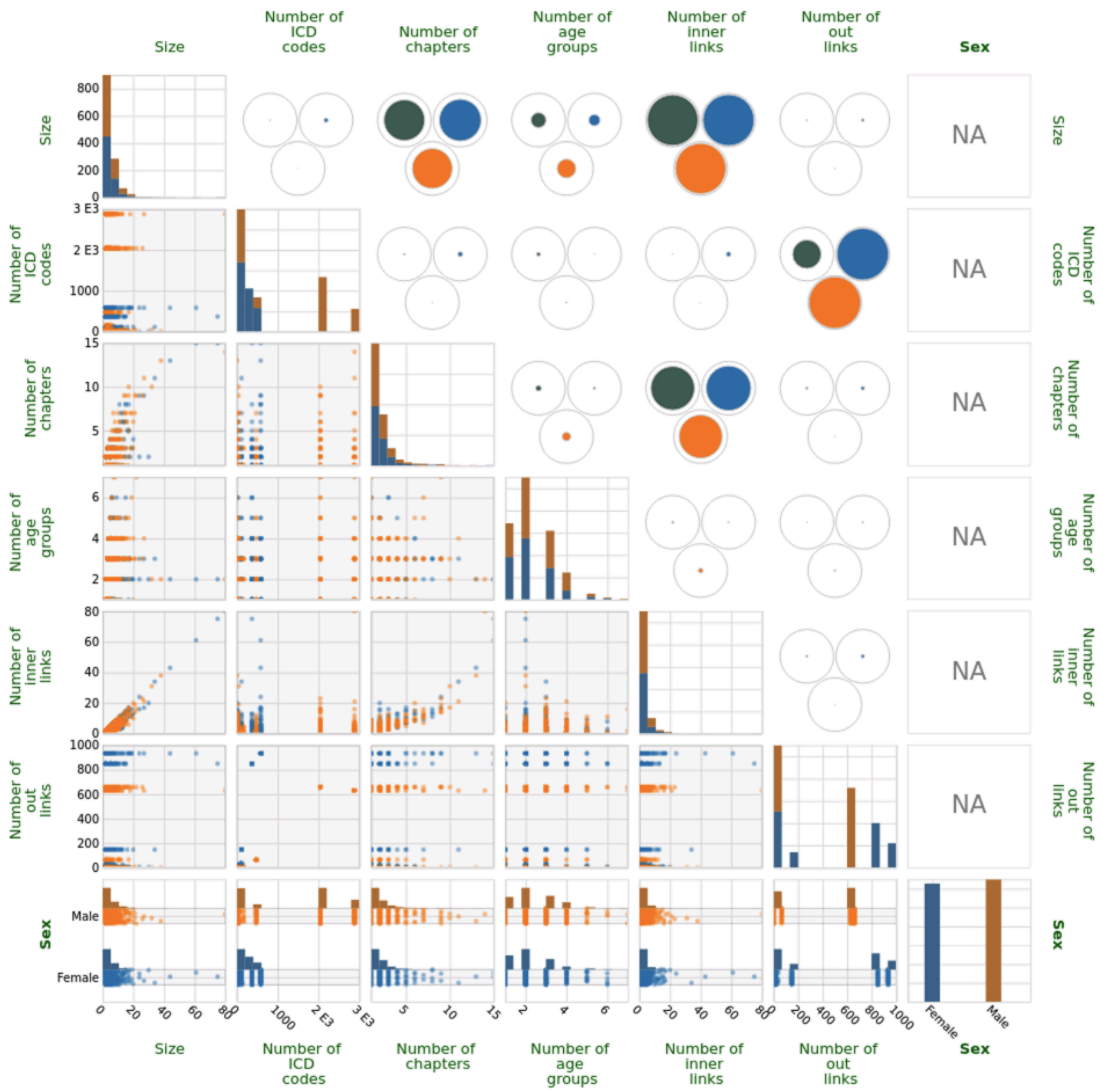

Supplementary Figure 7: Grid of scatterplots that shows the relationship between size, number of ICD codes, number of ICD chapters, and number of age groups, number of inner and out links of trajectories in females and males (trajectories smaller than 100 members and bigger than 2 members), online dynamic version: https://vis.csh.ac.at/comorbidity_network_graphics/matrix_cluster/

Supplementary Table 8: Literature Validation of Trajectories for Males: Trajectories that span two or more ICD chapters and have a '1' in the 'PubMed confirmed' column are supported by literature. However, trajectories marked with a '0' indicate that some diseases within that trajectory don't have evident associations with others, according to PubMed records.

Community_ID	Number of age groups	Number of ICD chapters	Number of ICD codes	PubMed confirmed
1	1	1	4	
2	1	4	5	1
3	1	3	3	1
4	2	3	3	1
5	2	4	8	1
6	1	6	20	1
7	4	1	1	
8	1	3	3	1
9	2	2	2	1
10	2	4	6	1
11	2	2	3	1
12	1	1	3	
13	3	9	16	1
14	3	1	1	
15	2	1	3	
16	3	1	1	
17	3	1	1	
18	2	4	5	1
19	4	2	2	1
20	3	2	2	1
21	1	3	5	1
22	2	1	5	
23	3	5	8	1
24	2	1	2	
25	1	1	4	
26	3	1	1	
27	2	1	5	
28	3	1	1	
29	1	1	6	
30	3	1	1	
31	2	1	3	
32	1	3	6	1
33	1	2	5	1
34	1	2	5	1

**35**	1	2	4	1
**36**	2	1	2	
**37**	3	1	1	
**38**	2	1	4	
**39**	2	1	4	
**40**	2	1	2	
**41**	2	2	3	1
**42**	1	1	3	
**43**	4	1	1	
**44**	7	1	2	
**45**	1	2	4	1
**46**	3	1	1	
**47**	3	1	2	
**48**	2	1	3	
**49**	3	1	1	
**50**	1	2	3	1
**51**	3	2	2	1
**52**	3	1	1	
**53**	3	2	2	1
**54**	1	2	3	1
**55**	1	1	3	
**56**	1	1	4	
**57**	2	2	8	1
**58**	4	1	1	
**59**	3	1	4	
**60**	4	1	2	
**61**	4	1	2	
**62**	3	1	5	
**63**	1	2	4	1
**64**	4	1	2	
**65**	1	1	3	
**66**	1	2	3	1
**67**	2	1	2	
**68**	1	2	4	1
**69**	3	1	1	
**70**	1	2	4	1
**71**	2	2	2	0
**72**	4	2	3	1
**73**	3	1	3	
**74**	2	2	5	1

75	2	1	2	
76	2	2	3	1
77	3	2	9	1
78	4	1	6	
79	4	3	10	1
80	1	2	3	0
81	2	2	3	1
82	3	1	3	
83	6	1	1	
84	1	1	3	
85	2	1	2	
86	3	1	1	
87	4	1	3	
88	3	1	1	
89	3	1	1	
90	1	1	3	
91	2	1	2	
92	2	1	2	
93	1	1	3	
94	2	2	4	1
95	1	1	3	
96	1	1	4	
97	2	1	8	
98	2	1	6	
99	1	1	3	
100	3	2	4	1
101	1	1	3	
102	2	2	2	1
103	1	1	3	
104	1	1	8	
105	1	1	4	
106	1	1	4	
107	3	1	1	
108	3	3	9	1
109	2	1	3	
110	4	2	2	1
111	4	3	5	1
112	4	3	6	1
113	2	2	2	0

**114**	4	2	5	1
**115**	2	2	2	0
**116**	4	1	1	
**117**	2	3	3	1
**118**	5	6	9	1
**119**	7	9	16	1
**120**	3	1	1	
**121**	3	1	3	
**122**	2	2	3	1
**123**	3	7	11	1
**124**	3	1	2	
**125**	2	1	5	
**126**	3	4	7	1
**127**	3	1	1	
**128**	3	1	2	
**129**	3	5	8	1
**130**	3	1	1	
**131**	3	1	6	
**132**	3	1	1	
**133**	3	3	5	1
**134**	2	2	7	1
**135**	2	1	3	
**136**	4	1	6	
**137**	3	1	3	
**138**	5	1	1	
**139**	4	1	3	
**140**	2	1	4	
**141**	2	1	3	
**142**	3	1	2	
**143**	3	1	1	
**144**	2	1	2	
**145**	2	1	3	
**146**	1	1	3	
**147**	2	1	4	
**148**	4	1	1	
**149**	3	1	2	
**150**	1	1	3	
**151**	5	2	5	1
**152**	1	1	4	

153	1	1	3	
154	5	2	3	1
155	2	2	5	0
156	4	3	8	1
157	1	1	4	
158	2	2	2	1
159	1	2	4	1
160	2	1	2	
161	1	1	5	
162	3	1	3	
163	2	1	3	
164	2	1	2	
165	3	3	5	1
166	2	1	2	
167	3	1	9	
168	4	2	6	1
169	4	1	3	
170	3	3	3	1
171	3	1	1	
172	1	2	3	1
173	2	1	2	
174	1	1	3	
175	4	2	4	1
176	4	4	6	1
177	2	2	3	1
178	2	2	2	1
179	2	3	4	1
180	2	2	2	0
181	2	2	2	1
182	2	1	3	
183	4	1	1	
184	4	2	2	1
185	3	3	7	1
186	4	2	3	1
187	1	1	3	
188	4	1	1	
189	6	7	11	1
190	2	3	5	1
191	3	2	4	1
192	4	1	1	

193	4	1	1	
194	2	2	3	1
195	2	5	8	1
196	2	1	2	
197	4	4	6	1
198	4	4	5	1
199	4	1	1	
200	3	9	14	1
201	4	1	2	
202	3	1	1	
203	2	3	7	1
204	4	1	1	
205	3	6	15	1
206	3	1	1	
207	4	2	2	1
208	3	1	1	
209	1	2	4	1
210	4	3	8	1
211	3	1	1	
212	4	3	9	1
213	2	2	2	1
214	4	1	1	
215	2	1	3	
216	3	1	4	
217	4	2	5	1
218	3	9	23	1
219	2	2	4	1
220	4	1	3	
221	4	1	4	
222	4	2	2	1
223	2	2	3	1
224	3	2	3	1
225	2	3	5	1
226	3	2	3	1
227	1	2	4	1
228	3	4	9	1
229	3	1	2	
230	1	1	3	
231	2	1	4	
232	5	1	6	

233	2	1	2	
234	1	2	3	1
235	2	3	6	1
236	2	3	3	0
237	4	1	1	
238	2	4	7	1
239	3	4	7	1
240	2	1	4	
241	4	1	1	
242	1	1	3	
243	4	4	4	1
244	4	5	5	1
245	3	1	1	
246	2	1	2	
247	1	1	4	
248	2	1	8	
249	4	1	1	
250	4	1	1	
251	3	1	5	
252	3	1	3	
253	3	2	4	1
254	3	1	1	
255	2	1	2	
256	2	2	2	1
257	3	10	17	1
258	3	1	1	
259	2	1	2	
260	3	1	4	
261	4	3	6	1
262	3	4	8	1
263	3	1	1	
264	2	2	4	1
265	2	1	2	
266	2	2	3	1
267	2	1	2	
268	3	9	14	1
269	1	3	3	1
270	3	6	9	1
271	3	5	9	1
272	4	4	6	1

273	1	4	7	1
274	3	7	13	1
275	3	1	1	
276	3	1	2	
277	2	1	3	
278	3	1	1	
279	3	2	7	1
280	3	1	1	
281	4	3	5	1
282	3	2	2	1
283	3	2	2	0
284	1	3	4	1
285	3	1	1	
286	3	1	1	
287	3	1	1	
288	3	1	1	
289	4	3	5	1
290	5	7	11	1
291	3	5	9	1
292	2	1	3	
293	4	1	1	
294	5	2	2	1
295	4	1	2	
296	5	1	6	
297	2	2	3	1
298	3	2	2	1
299	2	3	3	1
300	4	2	2	1
301	3	2	3	1
302	2	2	2	1
303	2	2	4	1
304	3	1	1	
305	4	3	7	1
306	5	1	2	
307	3	7	10	1
308	3	1	5	
309	3	1	1	
310	3	1	2	
311	3	1	1	
312	3	1	1	

**313**	3	1	3	
**314**	3	1	4	
**315**	2	3	3	0
**316**	3	1	1	
**317**	2	1	3	
**318**	5	1	7	
**319**	3	1	2	
**320**	2	4	5	1
**321**	3	1	1	
**322**	3	1	2	
**323**	3	3	4	1
**324**	2	1	3	
**325**	3	3	7	1
**326**	2	1	3	
**327**	1	2	3	1
**328**	3	1	1	
**329**	4	3	3	1
**330**	2	1	2	
**331**	3	1	1	
**332**	3	1	4	
**333**	1	1	3	
**334**	3	7	7	1
**335**	3	1	1	
**336**	3	2	2	1
**337**	3	1	1	
**338**	1	1	5	
**339**	3	1	1	
**340**	3	1	4	
**341**	6	1	1	
**342**	3	1	3	
**343**	3	3	4	1
**344**	2	1	3	
**345**	3	1	1	
**346**	3	4	8	1
**347**	3	1	4	
**348**	3	1	5	
**349**	3	1	1	
**350**	3	1	1	
**351**	2	14	80	1

**352**	4	2	2	0
**353**	3	2	3	1
**354**	1	3	3	1
**355**	2	2	2	1
**356**	2	3	4	1
**357**	3	1	2	
**358**	2	1	2	
**359**	2	2	3	1
**360**	3	1	2	
**361**	4	1	2	
**362**	2	2	2	1
**363**	2	2	5	1
**364**	2	3	6	1
**365**	3	3	4	1
**366**	4	2	6	1
**367**	4	2	2	1
**368**	1	2	3	1
**369**	3	2	8	1
**370**	2	2	4	1
**371**	5	1	1	
**372**	3	1	1	
**373**	3	2	4	1
**374**	2	10	31	1
**375**	3	9	19	1
**376**	3	4	8	1
**377**	2	1	2	
**378**	2	5	8	1
**379**	2	1	6	
**380**	2	1	3	
**381**	3	5	7	1
**382**	3	4	8	1
**383**	2	6	11	1
**384**	3	3	4	1
**385**	3	1	2	
**386**	3	7	14	1
**387**	2	1	2	
**388**	3	1	8	
**389**	1	2	8	1
**390**	4	11	21	1
**391**	5	1	2	
**392**	3	3	7	1

**393**	2	1	3	
**394**	2	13	38	1
**395**	2	1	3	
**396**	4	2	5	1
**397**	2	6	8	1
**398**	3	7	16	1
**399**	2	1	2	
**400**	2	1	2	
**401**	2	3	6	1
**402**	2	3	5	1
**403**	4	1	1	
**404**	1	1	3	
**405**	2	3	10	1
**406**	3	2	4	1
**407**	2	2	5	1
**408**	2	3	10	1
**409**	7	1	1	
**410**	2	2	5	1
**411**	2	3	4	1
**412**	1	1	3	
**413**	2	3	4	1
**414**	2	3	7	1
**415**	4	2	3	1
**416**	2	2	5	1
**417**	3	2	4	1
**418**	4	1	1	
**419**	2	1	3	
**420**	1	1	3	
**421**	3	1	9	
**422**	2	1	2	
**423**	3	2	7	1
**424**	2	2	12	1
**425**	3	3	7	1
**426**	1	1	3	
**427**	3	3	13	1
**428**	4	2	2	1
**429**	2	1	3	
**430**	2	2	3	1
**431**	2	2	4	1
**432**	3	2	3	1

**433**	1	2	3	1
**434**	1	3	4	1
**435**	4	2	2	1
**436**	2	4	6	1
**437**	1	3	5	1
**438**	1	5	12	1
**439**	1	2	3	1
**440**	3	2	6	1
**441**	3	4	5	1
**442**	1	2	4	1
**443**	4	2	5	1
**444**	1	2	6	1
**445**	4	5	13	1
**446**	4	1	4	
**447**	3	1	2	
**448**	1	1	5	
**449**	1	2	3	1
**450**	2	2	8	1
**451**	3	1	4	
**452**	1	1	4	
**453**	4	1	2	
**454**	1	2	5	1
**455**	1	1	3	
**456**	1	2	3	1
**457**	1	1	5	
**458**	1	1	3	
**459**	2	2	10	1
**460**	3	1	4	
**461**	4	3	5	1

Supplementary Table 9: Literature Validation of Trajectories for Females: Trajectories that span two or more ICD chapters and have a '1' in the 'PubMed confirmed' column are supported by literature. However, trajectories marked with a '0' indicate that some diseases within that trajectory don't have evident associations with others, according to PubMed records.

Community_ID	Number of age groups	Number of ICD chapters	Number of ICD codes	PubMed confirmed
**1**	2	2	4	1
**2**	2	4	5	1
**3**	3	6	15	1
**4**	2	2	2	1

**5**	2	3	7	1
**6**	1	3	3	1
**7**	1	4	11	1
**8**	2	2	3	1
**9**	2	3	5	1
**10**	3	1	1	
**11**	2	3	3	1
**12**	1	1	3	
**13**	2	3	8	1
**14**	1	1	3	
**15**	3	8	13	1
**16**	1	3	7	1
**17**	2	1	3	
**18**	3	2	5	1
**19**	3	1	1	
**20**	2	3	5	0
**21**	3	2	3	1
**22**	3	1	1	
**23**	3	1	3	
**24**	1	2	4	1
**25**	1	3	6	1
**26**	3	1	2	
**27**	2	2	4	0
**28**	1	1	3	
**29**	2	3	5	1
**30**	3	1	1	
**31**	6	1	1	
**32**	1	1	5	
**33**	1	1	3	
**34**	2	15	75	1
**35**	4	3	5	0
**36**	1	2	3	1
**37**	1	2	4	1
**38**	3	1	1	
**39**	3	1	4	
**40**	2	4	6	1
**41**	2	1	2	
**42**	3	2	2	1
**43**	1	2	3	1
**44**	1	1	3	

45	2	1	6	
46	1	2	7	1
47	2	1	2	
48	2	1	3	
49	3	2	3	1
50	1	1	3	
51	2	2	2	1
52	2	1	2	
53	2	2	4	1
54	1	2	3	1
55	2	2	3	1
56	1	1	4	
57	1	3	4	1
58	3	1	1	
59	1	1	5	
60	1	1	3	
61	1	1	6	
62	1	1	4	
63	2	2	3	0
64	1	3	3	1
65	1	2	8	1
66	1	2	4	1
67	1	2	3	1
68	1	2	4	1
69	1	3	5	1
70	1	3	7	1
71	5	2	4	1
72	3	1	1	
73	5	1	2	
74	2	1	4	
75	2	4	6	1
76	1	1	3	
77	3	1	1	
78	3	2	3	1
79	4	1	1	
80	3	1	1	
81	4	3	3	1
82	4	1	2	
83	3	3	3	0
84	2	1	2	

**85**	4	1	1	
**86**	1	1	3	
**87**	4	1	1	
**88**	1	1	3	
**89**	2	4	6	1
**90**	3	1	1	
**91**	2	1	3	
**92**	2	1	7	
**93**	1	3	8	1
**94**	6	1	1	
**95**	4	2	2	1
**96**	1	2	4	1
**97**	1	1	5	
**98**	2	2	2	1
**99**	1	1	5	
**100**	2	2	4	1
**101**	1	1	4	
**102**	1	3	3	1
**103**	3	2	8	1
**104**	3	1	1	
**105**	1	2	7	1
**106**	1	2	5	1
**107**	4	2	2	1
**108**	1	2	4	1
**109**	1	1	3	
**110**	1	2	7	1
**111**	1	2	3	1
**112**	1	2	3	1
**113**	3	1	1	
**114**	2	1	5	
**115**	2	3	5	1
**116**	3	1	1	
**117**	2	1	3	
**118**	2	1	2	
**119**	1	2	5	1
**120**	3	2	4	1
**121**	3	1	2	
**122**	2	2	5	1
**123**	3	1	3	
**124**	3	1	1	

**125**	3	2	2	1
**126**	2	2	4	1
**127**	4	2	4	1
**128**	2	3	5	1
**129**	1	1	3	
**130**	1	1	3	
**131**	2	1	2	
**132**	1	1	5	
**133**	3	1	2	
**134**	3	2	3	1
**135**	1	2	4	1
**136**	2	1	3	
**137**	1	1	3	
**138**	1	1	3	
**139**	3	1	5	
**140**	2	1	3	
**141**	2	1	2	
**142**	2	1	3	
**143**	1	2	4	1
**144**	1	1	5	
**145**	2	2	2	0
**146**	2	2	5	1
**147**	3	3	3	1
**148**	1	1	4	
**149**	2	1	2	
**150**	3	1	1	
**151**	5	2	2	1
**152**	3	1	3	
**153**	1	1	7	
**154**	3	1	4	
**155**	3	1	4	
**156**	3	3	5	1
**157**	2	1	7	
**158**	2	2	2	1
**159**	4	2	4	1
**160**	3	4	7	1
**161**	2	1	2	
**162**	4	1	1	
**163**	1	2	3	1
**164**	1	3	6	1

**165**	2	2	2	0
**166**	1	1	3	
**167**	2	3	5	1
**168**	2	3	3	1
**169**	1	2	4	1
**170**	1	2	5	1
**171**	2	2	2	1
**172**	2	1	2	
**173**	1	2	7	1
**174**	1	3	6	1
**175**	6	3	3	1
**176**	2	5	7	1
**177**	4	1	2	
**178**	2	1	4	
**179**	3	5	7	1
**180**	1	3	3	1
**181**	2	4	6	1
**182**	2	1	2	
**183**	1	1	3	
**184**	2	2	3	1
**185**	1	3	6	1
**186**	3	1	3	
**187**	3	1	2	
**188**	3	1	1	
**189**	4	3	3	1
**190**	2	1	2	
**191**	3	2	6	1
**192**	1	2	5	1
**193**	5	2	9	1
**194**	3	6	13	1
**195**	3	1	1	
**196**	3	1	1	
**197**	1	2	3	1
**198**	1	1	3	
**199**	2	1	2	
**200**	4	1	4	
**201**	2	4	13	1
**202**	2	1	6	
**203**	2	1	8	
**204**	4	1	5	

205	1	1	3	
206	4	1	3	
207	4	1	1	
208	2	4	8	1
209	2	2	4	1
210	2	1	2	
211	3	2	4	1
212	2	2	2	1
213	1	3	5	1
214	1	2	3	1
215	4	1	1	
216	3	1	1	
217	3	1	2	
218	2	1	2	
219	2	3	16	1
220	4	1	1	
221	2	1	2	
222	1	1	4	
223	4	2	2	1
224	3	1	1	
225	2	5	11	1
226	1	3	4	1
227	2	1	2	
228	2	1	7	
229	3	2	4	1
230	2	1	5	
231	1	1	3	
232	3	1	4	
233	4	1	1	
234	3	1	1	
235	2	3	12	1
236	3	9	24	1
237	2	1	6	
238	2	1	3	
239	3	2	4	1
240	2	2	5	1
241	2	1	3	
242	3	3	6	1
243	3	3	5	1
244	3	8	11	1

**245**	2	5	10	1
**246**	3	1	2	
**247**	3	1	1	
**248**	2	1	4	
**249**	1	3	6	0
**250**	2	3	3	1
**251**	2	2	4	1
**252**	2	3	6	1
**253**	1	1	3	
**254**	3	1	3	
**255**	3	1	2	
**256**	2	2	2	1
**257**	3	1	1	
**258**	2	1	2	
**259**	3	1	1	
**260**	3	1	1	
**261**	3	1	1	
**262**	2	2	5	1
**263**	3	1	1	
**264**	3	1	1	
**265**	3	4	9	1
**266**	3	1	1	
**267**	3	1	2	
**268**	2	2	3	1
**269**	1	3	6	1
**270**	2	4	5	1
**271**	3	1	1	
**272**	3	3	5	1
**273**	3	1	4	
**274**	3	2	4	1
**275**	3	8	16	1
**276**	4	2	3	1
**277**	2	1	2	
**278**	3	7	9	1
**279**	3	5	15	1
**280**	5	1	3	
**281**	3	2	4	1
**282**	2	2	2	1
**283**	3	1	2	
**284**	3	2	4	1

285	3	1	1	
286	3	1	1	
287	3	2	3	1
288	3	1	1	
289	2	2	3	1
290	2	1	4	
291	2	1	3	
292	3	1	4	
293	3	1	1	
294	2	1	4	
295	3	1	1	
296	3	2	4	1
297	2	1	3	
298	3	1	2	
299	3	1	1	
300	2	1	2	
301	3	1	1	
302	3	1	1	
303	2	1	4	
304	3	1	1	
305	3	1	1	
306	3	1	1	
307	3	1	3	
308	3	1	1	
309	3	1	3	
310	2	1	3	
311	1	2	4	1
312	3	4	4	1
313	2	2	3	1
314	3	1	1	
315	4	6	9	1
316	2	2	2	1
317	3	2	2	1
318	3	1	2	
319	2	1	7	
320	3	1	1	
321	3	1	1	
322	5	1	1	
323	4	1	2	

**324**	3	11	34	1
**325**	3	9	17	1
**326**	3	1	1	
**327**	2	2	5	1
**328**	2	1	3	
**329**	2	1	6	
**330**	2	1	2	
**331**	2	3	5	1
**332**	1	1	3	
**333**	1	2	4	1
**334**	2	2	4	1
**335**	2	15	61	1
**336**	3	2	2	1
**337**	3	3	5	0
**338**	3	2	3	1
**339**	3	1	4	
**340**	2	3	3	1
**341**	1	2	3	1
**342**	2	2	3	1
**343**	2	2	4	1
**344**	2	3	7	1
**345**	2	1	3	
**346**	2	2	4	1
**347**	2	2	2	0
**348**	2	2	2	1
**349**	4	2	2	0
**350**	2	4	13	1
**351**	3	1	5	
**352**	2	13	43	1
**353**	4	1	1	
**354**	3	6	13	1
**355**	2	3	5	1
**356**	5	1	1	
**357**	2	2	2	0
**358**	2	3	4	1
**359**	2	1	4	
**360**	3	1	1	
**361**	2	1	3	
**362**	5	1	1	
**363**	2	1	3	

**364**	2	5	7	1
**365**	3	5	7	1
**366**	2	1	2	
**367**	2	2	5	1
**368**	1	2	3	1
**369**	2	1	4	
**370**	2	7	12	1
**371**	6	1	1	
**372**	2	3	11	1
**373**	3	1	3	
**374**	3	3	7	1
**375**	4	6	9	1
**376**	2	2	3	1
**377**	3	1	3	
**378**	3	1	2	
**379**	2	2	3	0
**380**	1	2	3	0
**381**	5	1	1	
**382**	2	2	2	1
**383**	2	2	3	1
**384**	2	1	7	
**385**	3	1	3	
**386**	2	2	15	1
**387**	2	1	2	
**388**	2	1	3	
**389**	2	1	3	
**390**	4	1	3	
**391**	3	1	4	
**392**	4	1	1	
**393**	2	2	3	1
**394**	4	2	4	1
**395**	2	2	3	1
**396**	4	1	2	
**397**	6	3	8	1
**398**	1	1	3	
**399**	3	1	1	
**400**	4	1	2	
**401**	3	1	3	
**402**	5	4	4	1
**403**	2	2	4	1

**404**	2	1	2	
**405**	4	1	1	
**406**	3	5	15	1
**407**	5	1	3	
**408**	1	1	9	
**409**	2	2	3	0
**410**	2	1	9	
**411**	2	2	16	1
**412**	2	2	3	1
**413**	2	1	3	
**414**	4	1	1	
**415**	1	4	5	1
**416**	2	2	3	0
**417**	2	2	6	1
**418**	1	1	3	
**419**	2	3	9	1
**420**	1	2	3	1
**421**	3	1	2	
**422**	1	2	3	1
**423**	2	2	9	1
**424**	1	2	3	1
**425**	2	8	14	1
**426**	4	2	2	1
**427**	4	1	1	
**428**	1	2	5	1
**429**	1	1	4	
**430**	1	1	3	
**431**	3	2	11	1
**432**	2	1	3	
**433**	1	1	3	
**434**	1	5	17	1
**435**	4	1	4	
**436**	2	2	6	1
**437**	1	1	3	
**438**	2	2	6	1
**439**	5	1	1	
**440**	2	2	20	1
**441**	1	1	3	
**442**	2	2	5	1
**443**	3	2	2	1

**444**	1	2	7	1

Supplementary Table 10: All identified diverging trajectories in males.

Community_1	Community_2	
N32-40-49, N35-40-49, N32-50-59, N35-50-59	N32-40-49, N40-40-49, N41-40-49, N21-50-59, N40-50-59, N41-50-59, N42-50-59, N41-60-69, N42-60-69	Diverge
D52-40-49, F54-40-49, F55-40-49, J41-40-49, L71-40-49, F17-50-59, J41-50-59, J42-50-59, J95-50-59, J13-60-69, J39-60-69, K04-60-69, K08-60-69, K12-60-69, L08-60-69, N62-60-69	L71-40-49, K70-50-59, D53-60-69, F05-60-69, G31-60-69, G31-70-79	Diverge
N25-40-49, N26-40-49, N18-50-59, N25-50-59, N26-50-59, A04-60-69, B02-60-69, N03-60-69, N26-60-69, N26-70-79	F54-40-49, N26-40-49, I25-50-59, I97-50-59, I13-60-69, I30-60-69, I33-60-69, K04-60-69	Diverge
A02-60-69, A18-60-69, A26-60-69, A40-60-69, A48-60-69, A69-60-69, A84-60-69, B07-60-69, B36-60-69, B44-60-69, B49-60-69, B86-60-69, B91-60-69, C00-60-69, C06-60-69, C11-60-69, C12-60-69, C14-60-69, C26-60-69, C39-60-69, C41-60-69, C45-60-69, C50-60-69, C60-60-69, C66-60-69, C69-60-69, C76-60-69, C84-60-69, C88-60-69, D01-60-69, D03-60-69, D04-60-69, D07-60-69, D11-60-69, D15-60-69, D31-60-69, D34-60-69, D51-60-69, D89-60-69, E07-60-69, E22-60-69, E23-60-69, E27-60-69, E41-60-69, E46-60-69, E74-60-69, E85-60-69, F52-60-69, G06-60-69, G24-60-69, G52-60-69, G90-60-69, G92-60-69, H21-60-69, H31-60-69, H55-60-69, H61-60-69, H70-60-69, J02-60-69, J10-60-69, J11-60-69, J12-60-69, J21-60-69, J61-60-69, J85-60-69, K11-60-69, K28-60-69, K41-60-69, L25-60-69, L58-60-69, L60-60-69, L80-60-69, L81-60-69, M12-60-69, M60-60-69, N11-60-69, N34-60-69, N36-60-69, N50-60-69, 000-70-79	B36-60-69, D34-60-69, E72-60-69, F09-60-69, G90-60-69, I10-60-69, I30-60-69, I33-60-69, I60-60-69, I78-60-69, J13-60-69, J30-60-69, K73-60-69, L08-60-69, L21-60-69, L23-60-69, L27-60-69, L29-60-69, L71-60-69, L85-60-69, N02-60-69, N04-60-69, C05-70-79, C74-70-79, D15-70-79, E26-70-79, E29-70-79, F55-70-79, G37-70-79, G96-70-79, H72-70-79, I68-70-79, J35-70-79, K09-70-79, L70-70-79, M14-70-79, N03-70-79, N49-70-79	Diverge
E87-10-19, F10-10-19, F12-10-19, F17-10-19, F20-10-19, F23-10-19, F60-10-19, F60-20-29, F63-20-29	F20-10-19, F23-10-19, F12-20-29, F15-20-29, F20-20-29, F23-20-29, F25-20-29, F31-20-29, F20-30-39, F23-30-39, F25-30-39, F20-40-49, F25-40-49	Diverge
K29-30-39, K31-30-39, K51-30-39, K58-30-39, K59-30-39, K59-40-49	K51-30-39, K51-40-49, D73-50-59, J04-50-59, K51-50-59, B96-60-69, K29-60-69, K51-60-69, A69-70-79, D16-70-79, D21-70-79, K51-70-79, L50-70-79, N10-70-79, N62-70-79	Diverge
C61-40-49, N32-40-49, N40-40-49, C61-50-59, D40-50-59, N32-50-59	N32-40-49, N40-40-49, N41-40-49, N21-50-59, N40-50-59, N41-50-59, N42-50-59, N41-60-69, N42-60-69	Diverge
A41__0-9, C91__0-9, N10__0-9, A41-10-19, C91-10-19	N10__0-9, N13__0-9, N28__0-9, N28-10-19	Diverge
G35-20-29, E78-30-39, E87-30-39, G35-30-39, G45-30-39, H81-30-39, M42-30-39, M47-30-39, M47-40-49	G35-20-29, G35-30-39, G35-40-49, N31-40-49, N31-50-59	Diverge
E10-30-39, E16-30-39, H36-30-39, E10-40-49, E16-40-49, H36-40-49	H33-30-39, H35-30-39, H36-30-39, H43-40-49, H33-40-49, H34-40-49, H35-40-49, H36-40-49, H43-50-59, H33-50-59, H35-50-59, H36-50-59, H43-60-69, H26-60-69, H33-60-69	Diverge
E11-20-29, E66-20-29, E78-20-29, E79-20-29, G47-20-29, G47-30-39	G47-20-29, G25-30-39, G47-30-39, E66-40-49, G25-40-49, G47-40-49, G25-50-59, H66-50-59, K46-50-59	Diverge
G20-50-59, F02-60-69, G20-60-69, G21-60-69, G21-70-79	F32-50-59, G20-50-59, G35-50-59, F48-60-69, F61-60-69, G35-60-69, K02-60-69, N47-60-69, N48-60-69, G35-70-79, N47-70-79, N48-70-79	Diverge
E11-20-29, E66-20-29, E78-20-29, E79-20-29, G47-20-29, G47-30-39	E78-20-29, E79-20-29, E79-30-39	Diverge

B36-60-69, D34-60-69, E72-60-69, F09-60-69, G90-60-69, I10-60-69, I30-60-69, I33-60-69, I60-60-69, I78-60-69, J13-60-69, J30-60-69, K73-60-69, L08-60-69, L21-60-69, L23-60-69, L27-60-69, L29-60-69, L71-60-69, L85-60-69, N02-60-69, N04-60-69, C05-70-79, C74-70-79, D15-70-79, E26-70-79, E29-70-79, F55-70-79, G37-70-79, G96-70-79, H72-70-79, I68-70-79, J35-70-79, K09-70-79, L70-70-79, M14-70-79, N03-70-79, N49-70-79	F09-60-69, J62-60-69, J92-60-69, J44-70-79, J47-70-79, J62-70-79, K20-70-79	Diverge
D52-40-49, F54-40-49, F55-40-49, J41-40-49, L71-40-49, F17-50-59, J41-50-59, J42-50-59, J95-50-59, J13-60-69, J39-60-69, K04-60-69, K08-60-69, K12-60-69, L08-60-69, N62-60-69	J41-40-49, J44-50-59, J47-50-59, J95-50-59, B90-60-69, G61-60-69, H10-60-69, J40-60-69, J47-60-69, J82-60-69, J95-60-69	Diverge
N30-50-59, N43-50-59, D29-60-69, D30-60-69, N10-60-69, N39-60-69, N43-60-69, N45-60-69, D30-70-79, H10-70-79, N02-70-79, N39-70-79, N40-70-79, N41-70-79, N42-70-79, N43-70-79, N45-70-79, N99-70-79	N30-50-59, K63-60-69, C71-70-79, D36-70-79, D43-70-79	Diverge
L97-40-49, L98-40-49, L97-50-59, L98-50-59	L98-40-49, M86-40-49, L97-50-59, L98-50-59, M86-50-59, L97-60-69, L98-60-69, M86-60-69, L97-70-79, L98-70-79, M86-70-79	Diverge
M25-30-39, M25-40-49, M66-40-49, M87-40-49	M19-30-39, M24-30-39, M25-30-39, M65-30-39, M67-30-39, M75-30-39, M19-40-49, M24-40-49, M65-40-49, M67-40-49, M75-40-49, M77-40-49, M66-50-59, M75-50-59, M76-50-59, M77-50-59	Diverge
G40-40-49, G41-40-49, G81-40-49, J69-40-49, F70-50-59, G41-50-59, F70-60-69	G81-40-49, G81-50-59	Diverge
E87-10-19, F10-10-19, F12-10-19, F17-10-19, F20-10-19, F23-10-19, F60-10-19, F60-20-29, F63-20-29	F20-10-19, F23-10-19, F20-20-29, F23-20-29, F25-20-29, F20-30-39, F23-30-39, F25-30-39	Diverge
G20-50-59, F02-60-69, G20-60-69, G21-60-69, F02-70-79, G20-70-79, G21-70-79	F32-50-59, G20-50-59, G35-50-59, F48-60-69, F61-60-69, G35-60-69, K02-60-69, N47-60-69, N48-60-69, G35-70-79, N47-70-79, N48-70-79	Diverge
F17-40-49, G58-40-49, J01-40-49, J37-40-49, J38-40-49, J42-40-49, J43-40-49, J93-40-49, J02-50-59, J37-50-59, J38-50-59, J37-60-69, J37-70-79	J43-40-49, J43-50-59, J93-60-69	Diverge
F70-10-19, F70-20-29, D53-30-39, F10-30-39, F34-30-39, F70-30-39, D53-40-49, F05-40-49, F34-40-49, F70-40-49, G31-40-49, H10-40-49, K72-40-49, L21-40-49, D53-50-59	F70-10-19, F70-20-29, D75-30-39, F70-30-39, D52-40-49, F10-40-49, F22-40-49, F23-40-49, F70-40-49, G31-40-49, L21-40-49, C09-50-59, C10-50-59, C13-50-59, E53-50-59, F22-50-59, F55-50-59, H10-50-59, I86-50-59, N62-50-59, F22-60-69, F22-70-79	Diverge
B94-20-29, B16-30-39, B17-30-39, B18-30-39, F11-30-39, F12-30-39, F13-30-39, F14-30-39, F19-30-39, K73-30-39, B17-40-49, B18-40-49, B94-40-49, F11-40-49, F12-40-49, F14-40-49, F19-40-49, K73-40-49, F12-50-59, K73-50-59	B94-20-29, F17-30-39, G45-30-39, J06-30-39, J15-30-39, J20-30-39, J93-30-39, L05-40-49	Diverge
H33-30-39, H35-30-39, H36-30-39, H43-40-49, H33-40-49, H34-40-49, H35-40-49, H36-40-49, H43-50-59, H33-50-59, H35-50-59, H36-50-59, H43-60-69, H26-60-69, H33-60-69	E10-30-39, E16-30-39, H36-30-39, E16-40-49	Diverge
E88__0-9, F79__0-9, G25__0-9, G41__0-9, I47__0-9, J69__0-9, G40-10-19, G41-10-19, J96-10-19, J96-20-29	G40__0-9, G41__0-9, G47__0-9, G81__0-9, G91__0-9, G41-10-19, G47-10-19, G81-10-19, G91-10-19	Diverge
N32-40-49, N40-40-49, N41-40-49, N21-50-59, N40-50-59, N41-50-59, N42-50-59, N41-60-69, N42-60-69	N41-40-49, D40-50-59, N41-50-59, N42-50-59, D40-60-69, N41-60-69, C61-70-79, C64-70-79, D40-70-79, N41-70-79, N42-70-79	Diverge
B97__0-9, J20__0-9, J30__0-9, J42__0-9, L30__0-9, D80-10-19	J30__0-9, J30-10-19, J30-20-29	Diverge
E11-20-29, E66-20-29, E78-20-29, E79-20-29, G47-20-29, G47-30-39	G47-20-29, G25-30-39, G47-30-39, G25-40-49, G47-40-49	Diverge
D12-40-49, I84-40-49, K60-40-49, K61-40-49, K60-50-59, K61-50-59, K64-50-59	K60-40-49, K61-40-49, I84-50-59, K60-50-59, K61-50-59, D21-60-69	Diverge

I12-40-49, I13-40-49, N25-40-49, D44-50-59, E72-50-59, G61-50-59, I10-50-59, I12-50-59, I13-50-59, I30-50-59, I62-50-59, I99-50-59, J22-50-59, J91-50-59, L23-50-59, L27-50-59, L73-50-59, M35-50-59, M46-50-59, M80-50-59, N05-50-59, C31-60-69, E26-60-69, L05-60-69, N05-60-69, N12-60-69	N25-40-49, N26-40-49, N18-50-59, N25-50-59, N26-50-59, A04-60-69, B02-60-69, N03-60-69, N26-60-69, N26-70-79	Diverge
G91-40-49, I60-40-49, I61-40-49, G91-50-59, I60-50-59, I61-50-59	G91-40-49, I60-40-49, D73-50-59, F52-50-59, G30-50-59, G91-50-59, G99-50-59, I60-50-59, L28-50-59, E11-60-69, E55-60-69, F00-60-69, F05-60-69, G30-60-69, G91-60-69, H49-60-69, A26-70-79, A48-70-79, C76-70-79, D34-70-79, G91-70-79, H59-70-79, H60-70-79, L08-70-79, L28-70-79, M00-70-79, N04-70-79	Diverge
E87-10-19, F10-10-19, F12-10-19, F17-10-19, F20-10-19, F23-10-19, F60-10-19, F60-20-29, F63-20-29	F20-10-19, F23-10-19, F20-20-29, F23-20-29, F25-20-29, F20-30-39, F23-30-39, F25-30-39, F20-40-49, F25-40-49	Diverge
C32-40-49, J37-40-49, J38-40-49, C32-50-59, J37-50-59, J38-50-59	F17-40-49, G58-40-49, J01-40-49, J37-40-49, J38-40-49, J42-40-49, J43-40-49, J93-40-49, J02-50-59, J37-50-59, J38-50-59, J37-60-69, J37-70-79	Diverge
M70-50-59, E79-60-69, L82-60-69, M18-60-69, M70-60-69, H61-70-79, L21-70-79, M54-70-79, M70-70-79, M79-70-79	M70-50-59, M54-60-69, M70-60-69, B00-70-79, G43-70-79, G83-70-79, M76-70-79, M93-70-79	Diverge

Supplementary Table 11: All identified diverging trajectories in females.

Community_1	Community_2	
F48-40-49, G25-40-49, G47-40-49, F48-50-59, G25-50-59, G47-50-59	F06-40-49, F48-40-49, F32-50-59, F40-50-59, F44-50-59, F48-50-59, D16-60-69, F11-60-69, F22-60-69, F23-60-69, F44-60-69, F51-60-69, F55-60-69, J00-60-69, J37-60-69, L28-60-69, N89-60-69	Diverge
A38__0-9, B97__0-9, E45__0-9, G47__0-9, H04__0-9, J10__0-9, J35-10-19, J36-10-19, B07-20-29, B34-20-29, G51-20-29, H53-20-29, H72-20-29, H74-20-29, J15-20-29	B97__0-9, J10__0-9, J20__0-9, J10-10-19, J98-10-19, L22-10-19	Diverge
G83-50-59, H10-50-59, M54-60-69, M70-60-69, M76-60-69, J04-70-79, J31-70-79, M76-70-79	H10-50-59, F13-60-69, F33-60-69, F41-60-69, F43-60-69, F45-60-69, F13-70-79, F33-70-79, F41-70-79, F43-70-79, F45-70-79, K59-70-79	Diverge
N80-30-39, N84-30-39, N85-30-39, N88-30-39, N99-30-39, N99-40-49	D21-30-39, D25-30-39, D27-30-39, K66-30-39, N70-30-39, N71-30-39, N73-30-39, N80-30-39, N83-30-39, N84-30-39, N85-30-39, N88-30-39, N92-30-39, N93-30-39, N94-30-39, N99-30-39, K66-40-49, N70-40-49, N73-40-49	Diverge
F41-10-19, F45-10-19, F41-20-29	F44-10-19, F45-10-19, F45-20-29, F45-30-39, F45-40-49	Diverge
I21-40-49, I25-40-49, I42-40-49, I50-40-49, I20-50-59, I21-50-59, I24-50-59	I42-40-49, I50-40-49, I42-50-59, I44-50-59, I44-60-69, I44-70-79	Diverge
E66-40-49, I89-40-49, K42-40-49, K43-40-49, M16-40-49, E65-50-59, K42-50-59, K43-50-59	M16-40-49, E66-50-59, I89-50-59, L30-50-59, M16-50-59, M87-50-59, E65-60-69, I89-60-69, K45-60-69, L27-60-69, N62-60-69, I89-70-79	Diverge
M51-50-59, M96-50-59, A69-60-69, G54-60-69, G57-60-69, M96-60-69, G57-70-79	M96-50-59, G55-60-69, M51-60-69, M96-60-69	Diverge
B02-50-59, D13-50-59, E78-50-59, E83-50-59, G51-50-59, G58-50-59, I07-50-59, I15-50-59, I71-50-59, I72-50-59, I72-60-69	D13-50-59, K80-50-59, K81-50-59, K82-50-59, K91-60-69	Diverge
D17-50-59, D17-60-69, D17-70-79	D17-50-59, N84-60-69, N88-60-69	Diverge
C38-60-69, C78-60-69, D61-60-69, D70-60-69, J90-60-69, J91-60-69, C80-70-79, D70-70-79	D61-60-69, D69-60-69, D70-60-69, D46-70-79, D61-70-79, D69-70-79	Diverge
D61-60-69, D69-60-69, D70-60-69, D46-70-79, D61-70-79, D69-70-79	C77-60-69, C80-60-69, D63-60-69, D70-60-69, C23-70-79, C77-70-79, C78-70-79, C79-70-79, C80-70-79, D48-70-79, D63-70-79, D70-70-79, M84-70-79	Diverge
F31-20-29, F31-30-39, F31-40-49	F31-20-29, F10-30-39, F31-30-39, K70-30-39, K71-30-39, K71-40-49	Diverge

G54-40-49, G55-40-49, M41-40-49, M42-40-49, M43-40-49, M47-40-49, M48-40-49, M50-40-49, M53-40-49, M62-40-49, M99-40-49, E55-50-59, M41-50-59, M42-50-59, M43-50-59, M47-50-59, M48-50-59, M62-50-59, M99-50-59	M47-40-49, D34-50-59, D44-50-59, E04-50-59, E21-50-59, E55-50-59, E83-50-59, J38-50-59, E07-60-69	Diverge
A38__0-9, B97__0-9, E45__0-9, G47__0-9, H04__0-9, J10__0-9, J35-10-19, J36-10-19, B07-20-29, B34-20-29, G51-20-29, H53-20-29, H72-20-29, H74-20-29, J15-20-29	E45__0-9, E66__0-9, G47__0-9, H65-10-19, H90-10-19, D27-20-29, D39-20-29, F81-20-29, F93-20-29, H65-20-29, H66-20-29, H90-20-29, I88-20-29, J00-20-29, N83-20-29	Diverge
E66-40-49, I89-40-49, K42-40-49, K43-40-49, M16-40-49, E65-50-59, K42-50-59, K43-50-59	M16-40-49, I89-50-59, M16-50-59, M87-50-59, I89-60-69, I89-70-79	Diverge
E66-10-19, E78-10-19, F17-10-19, I10-10-19, K76-10-19, K80-10-19, K76-20-29	I10-10-19, I10-20-29, N18-20-29	Diverge
N80-30-39, N84-30-39, N85-30-39, N88-30-39, N92-30-39, N93-30-39, N99-30-39, N99-40-49	D21-30-39, D25-30-39, D27-30-39, K66-30-39, N70-30-39, N71-30-39, N73-30-39, N80-30-39, N83-30-39, N84-30-39, N85-30-39, N88-30-39, N92-30-39, N93-30-39, N94-30-39, N99-30-39, K66-40-49, N70-40-49, N73-40-49	Diverge
F31-20-29, F10-30-39, F31-30-39, K70-30-39, K71-30-39, K71-40-49	F31-20-29, F31-30-39, F31-40-49, F31-50-59, F31-60-69, F31-70-79	Diverge
F17-30-39, J20-30-39, J44-30-39, J43-40-49, J44-40-49, J96-40-49	J44-30-39, J45-30-39, J43-40-49, J44-40-49, J45-40-49, J96-40-49, J06-50-59	Diverge
C38-60-69, C77-60-69, C78-60-69, C79-60-69, C80-60-69, D61-60-69, D63-60-69, D70-60-69, J90-60-69, J91-60-69, M84-60-69, C23-70-79, C77-70-79, C78-70-79, C79-70-79, C80-70-79, D48-70-79, D63-70-79, D70-70-79, M84-70-79	D61-60-69, D69-60-69, D70-60-69, D46-70-79, D61-70-79, D69-70-79	Diverge
D53-40-49, F10-40-49, G62-40-49, I85-40-49, K71-40-49, K74-40-49, D53-50-59, F12-50-59, G62-50-59, K71-50-59	I85-40-49, K74-40-49, I85-50-59, K74-50-59	Diverge
F07-40-49, G40-40-49, G93-40-49, G41-50-59, G93-50-59	F07-40-49, G93-40-49, G40-50-59, G93-50-59, G41-60-69	Diverge
E11-20-29, E66-20-29, G47-20-29, M51-20-29, E65-30-39, L02-30-39	M51-20-29, G55-30-39, M43-30-39, M48-30-39, M51-30-39	Diverge
I21-40-49, I25-40-49, I42-40-49, I50-40-49, I20-50-59, I21-50-59, I24-50-59	E78-40-49, E79-40-49, G45-40-49, I20-40-49, I21-40-49, I44-40-49, I51-40-49, M41-40-49, E79-50-59, M41-50-59	Diverge
I21-40-49, I25-40-49, I42-40-49, I50-40-49, I20-50-59, I21-50-59, I24-50-59	I25-40-49, I42-40-49, I50-40-49, I42-50-59, I44-50-59, I50-50-59, I51-50-59, I42-60-69, I44-60-69, I44-70-79	Diverge
N80-50-59, N81-50-59, N84-50-59, N85-50-59, N88-50-59, N95-50-59, N80-60-69	N81-50-59, N81-60-69, N99-60-69	Diverge
D12-40-49, I84-40-49, K64-50-59	D12-40-49, D12-50-59, C18-60-69, C19-60-69, D12-60-69	Diverge
B02-50-59, D13-50-59, E78-50-59, E83-50-59, G51-50-59, G58-50-59, I07-50-59, I15-50-59, I71-50-59, I72-50-59, I72-60-69	I72-50-59, I67-60-69, I72-60-69, I67-70-79	Diverge
D17-50-59, D48-50-59, D17-60-69, D48-60-69, D17-70-79	D17-50-59, N84-60-69, N88-60-69	Diverge
E11-20-29, E66-20-29, G47-20-29, M51-20-29, E65-30-39, L02-30-39	M51-20-29, G55-30-39, M43-30-39, M48-30-39, M51-30-39, G54-40-49, G55-40-49, G57-40-49, M40-40-49, M51-40-49, M54-40-49, M96-40-49, G54-50-59, M96-50-59, M96-60-69	Diverge
E66-40-49, I89-40-49, K42-40-49, K43-40-49, M16-40-49, E65-50-59, K42-50-59, K43-50-59	M16-40-49, M16-50-59, M87-50-59	Diverge
I20-40-49, I21-40-49, I25-40-49, I42-40-49, I50-40-49, I20-50-59, I21-50-59, I24-50-59, I25-50-59, I42-50-59, I44-50-59, I50-50-59, I51-50-59	E78-40-49, E79-40-49, G45-40-49, I20-40-49, I21-40-49, I44-40-49, I51-40-49, M41-40-49, E79-50-59, M41-50-59	Diverge
E11-20-29, E66-20-29, G47-20-29, M51-20-29, E65-30-39, L02-30-39	M51-20-29, G55-30-39, M43-30-39, M48-30-39, M51-30-39, G55-40-49, G57-40-49, M51-40-49, M96-40-49	Diverge

M51-50-59, M96-50-59, A69-60-69, G54-60-69, G57-60-69, M96-60-69, G57-70-79	M96-50-59, G55-60-69, G95-60-69, M48-60-69, M50-60-69, M51-60-69, M96-60-69, G54-70-79, G55-70-79, G95-70-79, M47-70-79, M48-70-79, M50-70-79, M51-70-79, M54-70-79, M70-70-79, M93-70-79, M96-70-79	Diverge

Supplementary Table 12: Outcome of diverging trajectories in males.

															Ratio of average number (first vs. second trajectory) of number of					
Pair	Age	Number of the same patients	Number of patients of the first trajectory exclusively	Number of patients of the first trajectex clusively	Number of the patients from this age group, who are not following these two trajectories	Same diagnoses of trajectories, before they diverge	Exclusive diagnoses of the first trajectory	Exclusive diagnoses of the second trajectory	Average number of diagnosis of patients of the first trajectory	Average number of diagnosis of patients of the second trajectory	Average number of hospital days of patients of the first trajectory	Average number of hospital days of patients of the second trajectory	Average number of hospital stays of patients of the first trajectory	Average number of hospital stays of patients of the second trajectory	hospital diagnoses	days spent in hospital	hospital stays	Mortality of the first trajectory	Mortality of the sencond ttrajectory	Ratio of mortality
1	5	65	1496	339	330106	N32-40-49	N35-40-49<br>N32-50-59<br>N35-50-59	N40-40-49<br>N41-40-49<br>N21-50-59<br>N40-50-59<br>N41-50-59<br>N42-50-59<br>N41-60-69<br>N42-60-69	4.806	4.932	29.478	28.091	6.084	6.758	0.974	1.049	0.9	0	0.0006400468518	0.00
1	6	119	2215	1442	325503	N32-40-49	N35-40-49<br>N32-50-59<br>N35-50-59	N40-40-49<br>N41-40-49<br>N21-50-59<br>N40-50-59<br>N41-50-59<br>N42-50-59<br>N41-60-69<br>N42-60-69	6.469	5.741	41.978	35.267	8.864	8.877	1.127	1.19	0.999	0.00185890944	0.001249571169	1.49

5	2	6	30	920	160614	F20-10-19<br>F23-10-19	E87-10-19<br>F10-10-19<br>F12-10-19<br>F17-10-19<br>F60-10-19<br>F60-20-29<br>F63-20-29	F12-20-29<br>F15-20-29<br>F20-20-29<br>F23-20-29<br>F25-20-29<br>F31-20-29<br>F20-30-39<br>F23-30-39<br>F25-30-39<br>F20-40-49<br>F25-40-49	2.167	5.565	12.267	99.203	2.9	4.36	0.389	0.124	0.665	0.0008869430205	0.00296735905	0.30
5	3	33	3490	268	224345	F20-10-19<br>F23-10-19	E87-10-19<br>F10-10-19<br>F12-10-19<br>F17-10-19<br>F60-10-19<br>F60-20-29<br>F63-20-29	F12-20-29<br>F15-20-29<br>F20-20-29<br>F23-20-29<br>F25-20-29<br>F31-20-29<br>F20-30-39<br>F23-30-39<br>F25-30-39<br>F20-40-49<br>F25-40-49	5.242	4.287	69.627	95.642	6.085	4.049	1.223	0.728	1.503	0	0.001162097556	0.00

7	5	33	279	371	331323	N32-40-49<br>N40-40-49	C61-40-49<br>C61-50-59<br>D40-50-59<br>N32-50-59	N41-40-49<br>N21-50-59<br>N40-50-59<br>N41-50-59<br>N42-50-59<br>N41-60-69<br>N42-60-69	6.652	5.043	32.434	28.273	7.341	6.786	1.319	1.147	1.082	0.0003707346282	0.0006400468518	0.58
7	6	28	942	1533	326775	N32-40-49<br>N40-40-49	C61-40-49<br>C61-50-59<br>D40-50-59<br>N32-50-59	N41-40-49<br>N21-50-59<br>N40-50-59<br>N41-50-59<br>N42-50-59<br>N41-60-69<br>N42-60-69	6.224	5.879	33.037	35.95	7.055	9.01	1.059	0.919	0.783	0.004772711511	0.001249571169	3.82
8	1	11	192	610	293266	N10__0-9	A41__0-9<br>C91__0-9<br>A41-10-19<br>C91-10-19	N13__0-9<br>N28__0-9<br>N28-10-19	13.99	3.831	50.161	16.319	6.104	5.356	3.652	3.074	1.14	0.0007495765927	0	Inf
8	2	2	471	163	160934	N10__0-9	A41__0-9<br>C91__0-9<br>A41-10-19<br>C91-10-19	N13__0-9<br>N28__0-9<br>N28-10-19	28.968	5.822	117.386	29.117	8.344	6.184	4.976	4.032	1.349	0.04987296748	0.006060606061	8.23

10	4	1	213	226	268024	H36-30-39<br>H36-40-49	E10-30-39<br>E16-30-39<br>E10-40-49<br>E16-40-49	H33-30-39<br>H35-30-39<br>H43-40-49<br>H33-40-49<br>H34-40-49<br>H35-40-49<br>H43-50-59<br>H33-50-59<br>H35-50-59<br>H36-50-59<br>H43-60-69<br>H26-60-69<br>H33-60-69	4.056	4.509	26.925	19.279	6.216	5.301	0.9	1.397	1.173	0	0	NA
10	5	0	361	116	331529	H36-30-39<br>H36-40-49	E10-30-39<br>E16-30-39<br>E10-40-49<br>E16-40-49	H33-30-39<br>H35-30-39<br>H43-40-49<br>H33-40-49<br>H34-40-49<br>H35-40-49<br>H43-50-59<br>H33-50-59<br>H35-50-59<br>H36-50-59<br>H43-60-69<br>H26-60-69<br>H33-60-69	6.675	5.517	50.764	27.181	10.314	6.819	1.21	1.868	1.513	0.003736442512	0.00243793546	1.53

11	3	22	172	2372	225570	G47-20-29<br>G47-30-39	E11-20-29<br>E66-20-29<br>E78-20-29<br>E79-20-29	G25-30-39<br>E66-40-49<br>G25-40-49<br>G47-40-49<br>G25-50-59<br>H66-50-59<br>K46-50-59	3.343	3.274	25.326	16.366	5.878	3.797	1.021	1.547	1.548	0.0004152145326	0.000417710944	0.99
11	4	6778	115	316	261256	G47-20-29<br>G47-30-39	E11-20-29<br>E66-20-29<br>E78-20-29<br>E79-20-29	G25-30-39<br>E66-40-49<br>G25-40-49<br>G47-40-49<br>G25-50-59<br>H66-50-59<br>K46-50-59	3.87	5.095	19.383	58.595	4.383	7.959	0.76	0.331	0.551	0.000290149427	0.000290149427	1.00
13	3	107	87	3422	224520	E78-20-29<br>E79-20-29	E11-20-29<br>E66-20-29<br>G47-20-29<br>G47-30-39	E79-30-39	3.379	3.836	19.115	33.534	5.632	6.128	0.881	0.57	0.919	0.0004152145326	0	Inf
13	4	107	6786	2362	259210	E78-20-29<br>E79-20-29	E11-20-29<br>E66-20-29<br>G47-20-29<br>G47-30-39	E79-30-39	3.545	4.43	16.011	34.592	4.126	7.923	0.8	0.463	0.521	0.000290149427	0.0004050222762	0.72

17	5	695	436	545	330330	L98-40-49<br>L97-50-59<br>L98-50-59	L97-40-4<br>9	M86-40-4<br>9<br>M86-50-5<br>9<br>L97-60-69<br>L98-60-69<br>M86-60-6<br>9<br>L97-70-79<br>L98-70-79<br>M86-70-7<br>9	7.312	5.512	65.617	47.108	11.161	8.327	1.327	1.393	1.34	0.00550<br>9527755	0.0045267<br>04995	1.22
17	6	234	1627	77	327341	L98-40-49<br>L97-50-59<br>L98-50-59	L97-40-4<br>9	M86-40-4<br>9<br>M86-50-5<br>9<br>L97-60-69<br>L98-60-69<br>M86-60-6<br>9<br>L97-70-79<br>L98-70-79<br>M86-70-7<br>9	7.95	6.558	76.56	79.104	13.338	13.39	1.212	0.968	0.996	0.01529<br>484971	0.0085354<br>76411	1.79
18	4	40	2377	214	265834	M25-30-39	M25-40-<br>49<br>M66-40-<br>49<br>M87-40-<br>49	M19-30-3<br>9<br>M24-30-3<br>9<br>M65-30-3<br>9<br>M67-30-3<br>9<br>M75-30-3<br>9<br>M19-40-4<br>9<br>M24-40-4<br>9<br>M65-40-4<br>9<br>M67-40-4<br>9<br>M75-40-4<br>9<br>M77-40-4<br>9<br>M66-50-5<br>9<br>M75-50-5<br>9<br>M76-50-5<br>9	3.125	2.439	17.008	10.785	5.21	3.808	1.281	1.577	1.368	0.00041<br>3736036<br>4	0	Inf

								M77-50-59												
18	5	1	249	413	331342	M25-30-39	M25-40-49<br>M66-40-49<br>M87-40-49	M19-30-39<br>M24-30-39<br>M65-30-39<br>M67-30-39<br>M75-30-39<br>M19-40-49<br>M24-40-49<br>M65-40-49<br>M67-40-49<br>M75-40-49<br>M77-40-49<br>M66-50-59<br>M75-50-59<br>M76-50-59<br>M77-50-59	4.072	4.019	25.614	18.918	6.329	5.525	1.013	1.354	1.146	0.0005633802817	0.0003393282894	1.66
19	2	6	30	920	160614	F20-10-19<br>F23-10-19	E87-10-19<br>F10-10-19<br>F12-10-19<br>F17-10-19<br>F60-10-19<br>F60-20-29<br>F63-20-29	F20-20-29<br>F23-20-29<br>F25-20-29<br>F20-30-39<br>F23-30-39<br>F25-30-39	2.167	5.565	12.267	99.203	2.9	4.36	0.389	0.124	0.665	0.000886943020 5	0.00296735905	0.30

19	3	79	3444	683	223930	F20-10-19<br>F23-10-19	E87-10-19<br>F10-10-19<br>F12-10-19<br>F17-10-19<br>F60-10-19<br>F60-20-29<br>F63-20-29	F20-20-29<br>F23-20-29<br>F25-20-29<br>F20-30-39<br>F23-30-39<br>F25-30-39	5.192	4.682	68.315	84.318	6.073	3.958	1.109	0.81	1.534	0	0.001288053936	0.00
24	4	1	226	213	268024	H36-30-39	H33-30-39<br>H35-30-39<br>H43-40-49<br>H33-40-49<br>H34-40-49<br>H35-40-49<br>H36-40-49<br>H43-50-59<br>H33-50-59<br>H35-50-59<br>H36-50-59<br>H43-60-69<br>H26-60-69<br>H33-60-69	E10-30-39<br>E16-30-39<br>E16-40-49	4.509	4.056	19.279	26.925	5.301	6.216	1.112	0.716	0.853	0	0	NA

24	5	1	115	335	331555	H36-30-39	H33-30-39<br>H35-30-39<br>H43-40-49<br>H33-40-49<br>H34-40-49<br>H35-40-49<br>H36-40-49<br>H43-50-59<br>H33-50-59<br>H35-50-59<br>H36-50-59<br>H43-60-69<br>H26-60-69<br>H33-60-69	E10-30-39<br>E16-30-39<br>E16-40-49	5.296	6.934	25.548	51.332	6.583	10.617	0.764	0.498	0.62	0.00243793546	0.02380952381	0.10
26	5	157	247	1402	330200	N41-40-49<br>N41-50-59<br>N42-50-59<br>N41-60-69	N32-40-49<br>N40-40-49<br>N21-50-59<br>N40-50-59<br>N42-60-69	D40-50-59<br>D40-60-69<br>C61-70-79<br>C64-70-79<br>D40-70-79<br>N41-70-79<br>N42-70-79	6.004	4.248	35.887	24.705	7.668	6.108	1.413	1.453	1.255	0.0006400468518	0.0006414368185	1.00
26	6	56	1505	420	327297	N41-40-49<br>N41-50-59<br>N42-50-59<br>N41-60-69	N32-40-49<br>N40-40-49<br>N21-50-59<br>N40-50-59	D40-50-59<br>D40-60-69<br>C61-70-79<br>C64-70-79<br>D40-70-7	5.934	5.55	36.086	30.707	9.105	7.61	1.069	1.175	1.196	0.001249571169	0.001252051143	1.00

							N42-60-<br>69	9<br>N41-70-7<br>9<br>N42-70-7<br>9												
26	7	3253	1220	3601	260446	N41-40-49<br>N41-50-59<br>N42-50-59<br>N41-60-69	N32-40-<br>49<br>N40-40-<br>49<br>N21-50-<br>59<br>N40-50-<br>59<br>N42-60-<br>69	D40-50-5<br>9<br>D40-60-6<br>9<br>C61-70-7<br>9<br>C64-70-7<br>9<br>D40-70-7<br>9<br>N41-70-7<br>9<br>N42-70-7<br>9	6.988	7.16	41.662	38.714	10.234	9.041	0.976	1.076	1.132	0	0.0011158<br>43341	0.00
27	3	22	172	2372	225570	G47-20-29<br>G47-30-39	E11-20-2<br>9<br>E66-20-<br>29<br>E78-20-<br>29<br>E79-20-<br>29	G25-30-3<br>9<br>G25-40-4<br>9<br>G47-40-4<br>9	3.343	3.274	25.326	16.366	5.878	3.797	1.021	1.547	1.548	0.00041<br>5214532<br>6	0.0004177<br>10944	0.99
27	4	6778	115	316	261256	G47-20-29<br>G47-30-39	E11-20-2<br>9<br>E66-20-<br>29<br>E78-20-<br>29<br>E79-20-<br>29	G25-30-3<br>9<br>G25-40-4<br>9<br>G47-40-4<br>9	3.87	5.095	19.383	58.595	4.383	7.959	0.76	0.331	0.551	0.00029<br>0149427	0.0002901<br>49427	1.00
28	5	836	1319	5872	323979	K60-40-49<br>K61-40-49<br>K60-50-59<br>K61-50-59	D12-40-<br>49<br>I84-40-4<br>9<br>K64-50-<br>59	I84-50-59<br>D21-60-6<br>9	4.087	3.929	18.363	21.105	5.647	5.123	1.04	0.87	1.102	0.00119<br>1236991	0.0011927<br>4659	1.00
28	6	51	620	1172	327436	K60-40-49<br>K61-40-49<br>K60-50-59<br>K61-50-59	D12-40-<br>49<br>I84-40-4<br>9<br>K64-50-<br>59	I84-50-59<br>D21-60-6<br>9	5.582	4.781	34.89	26.066	7.997	7.713	1.168	1.339	1.037	0.00207<br>2950899	0.0042545<br>18971	0.49

31	2	6	30	920	160614	F20-10-19<br>F23-10-19	E87-10-19<br>F10-10-19<br>F12-10-19<br>F17-10-19<br>F60-10-19<br>F60-20-29<br>F63-20-29	F20-20-29<br>F23-20-29<br>F25-20-29<br>F20-30-39<br>F23-30-39<br>F25-30-39<br>F20-40-49<br>F25-40-49	2.167	5.565	12.267	99.203	2.9	4.36	0.389	0.124	0.665	0.0008869430205	0.00296735905	0.30
31	3	79	3444	683	223930	F20-10-19<br>F23-10-19	E87-10-19<br>F10-10-19<br>F12-10-19<br>F17-10-19<br>F60-10-19<br>F60-20-29<br>F63-20-29	F20-20-29<br>F23-20-29<br>F25-20-29<br>F20-30-39<br>F23-30-39<br>F25-30-39<br>F20-40-49<br>F25-40-49	5.192	4.682	68.315	84.318	6.073	3.958	1.109	0.81	1.534	0	0.001288053936	0.00

Supplementary Table 13: Outcome of diverging trajectories in females.

															Ratio of average number (first vs. second trajectory) of number of			

Pair	Age	Number of the same patients	Number of patients of the first trajectory	Number of patients of the first trajectory	Number of the patients from this age group, who are not following these two trajectories	Same diagnoses of trajectories, before they diverge	Exclusive diagnoses of the first trajectory	Exclusive diagnoses of the second trajectory	Average number of diagnosis of patients of the first trajectory	Average number of diagnosis of patients of the second trajectory	Average number of hospital days of patients of the first trajectory	Average number of hospital days of patients of the second trajectory	Average number of hospital stays of patients of the first trajectory	Average number of hospital stays of patients of the second trajectory	hospital diagnoses	days spent in hospital	hospital stays	Mortality of the first trajectory	Mortality of the sencond ttrajectory	Ratio of mortality
1	5	35	122	1345	338227	F48-40-49<br>F48-50-59	G25-40-49<br>G47-40-49<br>G25-50-59<br>G47-50-59	F06-40-49<br>F32-50-59<br>F40-50-59<br>F44-50-59<br>D16-60-69<br>F11-60-69<br>F22-60-69<br>F23-60-69<br>F44-60-69<br>F51-60-69<br>F55-60-69<br>J00-60-69<br>J37-60-69<br>L28-60-69<br>N89-60-69	5.82	6.779	39.885	63.533	7.926	9.541	0.859	0.628	0.831	0.0003247397693	0.002050280693	0.16
1	6	2	240	464	293330	F48-40-49<br>F48-50-59	G25-40-49<br>G47-40-49<br>G25-50-59<br>G47-50-59	F06-40-49<br>F32-50-59<br>F40-50-59<br>F44-50-59<br>D16-60-69<br>F11-60-69<br>F22-60-69<br>F23-60-69<br>F44-60-69<br>F51-60-69<br>F55-60-69<br>J00-60-69<br>J37-60-69<br>L28-60-69<br>N89-60-69	6.567	6.504	40.654	56.933	9.583	10.478	1.01	0.714	0.915	0.001935872287	0.001375372001	1.41
5	2	2772	1366	810	174853	F45-10-19	F41-10-19<br>F41-20-29	F44-10-19<br>F45-20-29<br>F45-30-39<br>F45-40-49	3.889	4.921	32.892	43.328	4.537	5.621	0.79	0.759	0.807	0	0	NA
5	3	281	3243	1876	256213	F45-10-19	F41-10-19<br>F41-20-29	F44-10-19<br>F45-20-29<br>F45-30-39<br>F45-40-49	3.953	3.778	36.607	31.165	5.075	5.201	1.046	1.175	0.976	0	0	NA

6	5	89	1083	1020	337537	I42-40-49<br>I50-40-49	I21-40-49<br>I25-40-49<br>I20-50-59<br>I21-50-59<br>I24-50-59	I42-50-59<br>I44-50-59<br>I44-60-69<br>I44-70-79	5.149	6.715	33.148	50.691	8.803	11.01	0.767	0.654	0.8	0.09892<br>979526	0.103745<br>361	0.95
6	6	34	635	2008	291360	I42-40-49<br>I50-40-49	I21-40-49<br>I25-40-49<br>I20-50-59<br>I21-50-59<br>I24-50-59	I42-50-59<br>I44-50-59<br>I44-60-69<br>I44-70-79	5.011	6.243	30.602	46.577	9	11.405	0.803	0.657	0.7<br>89	0.02460<br>949692	0.005544<br>134826	4.44
7	5	10	50	3283	336386	M16-40-49	E66-40-49<br>I89-40-49<br>K42-40-49<br>K43-40-49<br>E65-50-59<br>K42-50-59<br>K43-50-59	E66-50-59<br>I89-50-59<br>L30-50-59<br>M16-50-59<br>M87-50-59<br>E65-60-69<br>I89-60-69<br>K45-60-69<br>L27-60-69<br>N62-60-69<br>I89-70-79	4.48	4.25	28.76	30.951	8.32	5.875	1.054	0.929	1.4<br>16	0	0	NA
7	6	0	250	70	293717	M16-40-49	E66-40-49<br>I89-40-49<br>K42-40-49<br>K43-40-49<br>E65-50-59<br>K42-50-59<br>K43-50-59	E66-50-59<br>I89-50-59<br>L30-50-59<br>M16-50-59<br>M87-50-59<br>E65-60-69<br>I89-60-69<br>K45-60-69<br>L27-60-69<br>N62-60-69<br>I89-70-79	6.647	6.057	42.462	53.6	9.353	10.743	1.097	0.792	0.8<br>71	0.00390<br>0074178	0	Inf
8	6	380	12579	120	280958	M96-50-59<br>M96-60-69	M51-50-59<br>A69-60-69<br>G54-60-69<br>G57-60-69<br>G57-70-79	G55-60-69<br>M51-60-69	5.748	7.333	37.359	54.067	8.693	10.458	0.784	0.691	0.8<br>31	0.00199<br>8589563	0.002	1.00
8	7	0	29	596	232659	M96-50-59<br>M96-60-69	M51-50-59<br>A69-60-69<br>G54-60-69<br>G57-60-69<br>G57-70-79	G55-60-69<br>M51-60-69	6.586	7.653	40.483	53.196	10.586	12.201	0.861	0.761	0.8<br>68	0.00218<br>7571886	0.002204<br>067934	0.99
11	7	12	241	367	232664	D61-60-69<br>D70-60-69	C38-60-69<br>C78-60-69<br>J90-60-69	D69-60-69<br>D46-70-79	15.614	16.777	88.859	99.747	13.519	16.033	0.931	0.891	0.8<br>43	0.06222<br>659619	0.036940<br>51803	1.68

							J91-60-69<br>C80-70-79<br>D70-70-79	D61-70-79<br>D69-70-79												
11	8	29	2138	226	217231	D61-60-69<br>D70-60-69	C38-60-69<br>C78-60-69<br>J90-60-69<br>J91-60-69<br>C80-70-79<br>D70-70-79	D69-60-69<br>D46-70-79<br>D61-70-79<br>D69-70-79	14.27	11.487	90.006	84.168	14.745	17.584	1.242	1.069	0.839	0.05858531023	0.1096844325	0.53
12	7	27	352	773	232132	D70-60-69	D61-60-69<br>D69-60-69<br>D46-70-79<br>D61-70-79<br>D69-70-79	C77-60-69<br>C80-60-69<br>D63-60-69<br>C23-70-79<br>C77-70-79<br>C78-70-79<br>C79-70-79<br>C80-70-79<br>D48-70-79<br>D63-70-79<br>D70-70-79<br>M84-70-79	16.753	19.331	100.199	95.03	15.79	13.287	0.867	1.054	1.188	0.03694051803	0.02535817937	1.46
12	8	0	255	54	219314	D70-60-69	D61-60-69<br>D69-60-69<br>D46-70-79<br>D61-70-79<br>D69-70-79	C77-60-69<br>C80-60-69<br>D63-60-69<br>C23-70-79<br>C77-70-79<br>C78-70-79<br>C79-70-79<br>C80-70-79<br>D48-70-79<br>D63-70-79<br>D70-70-79<br>M84-70-79	13.204	13.019	89.655	88.907	17.875	13.093	1.014	1.008	1.365	0.1096844325	0.005873087513	18.68
16	5	10	50	3283	336386	M16-40-49	E66-40-49<br>I89-40-49<br>K42-40-49<br>K43-40-49<br>E65-50-59<br>K42-50-59<br>K43-50-59	I89-50-59<br>M16-50-59<br>M87-50-59<br>I89-60-69<br>I89-70-79	4.48	4.25	28.76	30.951	8.32	5.875	1.054	0.929	1.416	0	0	NA
16	6	1	249	322	293465	M16-40-49	E66-40-49<br>I89-40-49<br>K42-40-49<br>K43-40-49<br>E65-50-59<br>K42-50-59<br>K43-50-59	I89-50-59<br>M16-50-59<br>M87-50-59<br>I89-60-69<br>I89-70-79	6.593	6.034	42.46	43.913	9.347	8.376	1.093	0.967	1.116	0.003900074178	0	Inf

17	2	19	106	683	178993	I10-10-19	E66-10-19<br>E78-10-19<br>F17-10-19<br>K76-10-19<br>K80-10-19<br>K76-20-29	I10-20-29<br>N18-20-29	3.104	4.812	20.453	27.959	4.745	6.161	0.645	0.732	0.7<br>7	0	0.002849<br>002849	0.00
17	3	92	1027	2289	258205	I10-10-19	E66-10-19<br>E78-10-19<br>F17-10-19<br>K76-10-19<br>K80-10-19<br>K76-20-29	I10-20-29<br>N18-20-29	4.261	4.477	30.73	29.216	6.904	6.3	0.952	1.052	1.0<br>96	0.00357<br>4620197	0.017129<br>88314	0.21
20	4	60	294	3442	290156	J44-30-39<br>J43-40-49<br>J44-40-49<br>J96-40-49	F17-30-39<br>J20-30-39	J45-30-39<br>J45-40-49<br>J06-50-59	5.214	4.575	39.316	29.991	7.384	6.69	1.14	1.311	1.1<br>04	0.00333<br>9778235	0.001725<br>505169	1.94
20	5	28	259	710	338731	J44-30-39<br>J43-40-49<br>J44-40-49<br>J96-40-49	F17-30-39<br>J20-30-39	J45-30-39<br>J45-40-49<br>J06-50-59	6.965	6.161	51.815	40.262	10.166	9.325	1.13	1.287	1.0<br>9	0.16878<br>94148	0.168070<br>014	1.00
21	7	0	45	379	232860	D61-60-69<br>D70-60-69	C38-60-69<br>C77-60-69<br>C78-60-69<br>C79-60-69<br>C80-60-69<br>D63-60-69<br>J90-60-69<br>J91-60-69<br>M84-60-69<br>C23-70-79<br>C77-70-79<br>C78-70-79<br>C79-70-79<br>C80-70-79<br>D48-70-79<br>D63-70-79<br>D70-70-79<br>M84-70-79	D69-60-69<br>D46-70-79<br>D61-70-79<br>D69-70-79	19.644	16.897	82.711	100.145	12.978	16.032	1.163	0.826	0.8<br>1	0.00586<br>4607725	0.036940<br>51803	0.16

21	8	0	54	255	219314	D61-60-69<br>D70-60-69	C38-60-69<br>C77-60-69<br>C78-60-69<br>C79-60-69<br>C80-60-69<br>D63-60-69<br>J90-60-69<br>J91-60-69<br>M84-60-69<br>C23-70-79<br>C77-70-79<br>C78-70-79<br>C79-70-79<br>C80-70-79<br>D48-70-79<br>D63-70-79<br>D70-70-79<br>M84-70-79	D69-60-69<br>D46-70-79<br>D61-70-79<br>D69-70-79	13.019	13.204	88.907	89.655	13.093	17.875	0.986	0.992	0.7<br>32	0.00587<br>3087513	0.109684<br>4325	0.05
22	5	21	178	661	338869	I85-40-49<br>K74-40-49	D53-40-49<br>F10-40-49<br>G62-40-49<br>K71-40-49<br>D53-50-59<br>F12-50-59<br>G62-50-59<br>K71-50-59	I85-50-59<br>K74-50-59	6.461	7.261	73.77	62.861	8.5	11.992	0.89	1.174	0.7<br>09	0.08494<br>135819	0.089667<br>53033	0.95
22	6	6	64	1194	292773	I85-40-49<br>K74-40-49	D53-40-49<br>F10-40-49<br>G62-40-49<br>K71-40-49<br>D53-50-59<br>F12-50-59<br>G62-50-59<br>K71-50-59	I85-50-59<br>K74-50-59	10.594	7.163	81.469	62.547	14.484	12.906	1.479	1.303	1.1<br>22	0.01490<br>278797	0.124659<br>2251	0.12
23	5	49	164	1090	338426	F07-40-49<br>G93-40-49<br>G93-50-59	G40-40-49<br>G41-50-59	G40-50-59<br>G41-60-69	6.726	7.009	47.86	80.524	8.689	9.997	0.96	0.594	0.8<br>69	0.20643<br>90873	0.210690<br>8247	0.98
23	6	915	142	2937	290043	F07-40-49<br>G93-40-49<br>G93-50-59	G40-40-49<br>G41-50-59	G40-50-59<br>G41-60-69	6.789	6.748	62.951	57.157	10.43	9.668	1.006	1.101	1.0<br>79	0.21375<br>21155	0.224390<br>5331	0.95
24	3	127	315	2837	258334	M51-20-29	E11-20-29<br>E66-20-29<br>G47-20-29	G55-30-39<br>M43-30-39	3.467	3.18	22.508	19.25	4.921	4.105	1.09	1.169	1.1<br>99	0	0	NA

							E65-30-39<br>L02-30-39	M48-30-39<br>M51-30-39												
24	4	7	3904	738	289303	M51-20-29	E11-20-29<br>E66-20-29<br>G47-20-29<br>E65-30-39<br>L02-30-39	G55-30-39<br>M43-30-39<br>M48-30-39<br>M51-30-39	3.83	3.449	20.633	21.692	4.649	4.585	1.11	0.951	1.0<br>14	0	0.000220<br>6184518	0.00
26	5	100	1072	306	338251	I25-40-49<br>I42-40-49<br>I50-40-49	I21-40-49<br>I20-50-59<br>I21-50-59<br>I24-50-59	I42-50-59<br>I44-50-59<br>I50-50-59<br>I51-50-59<br>I42-60-69<br>I44-60-69<br>I44-70-79	5.349	6.634	35.91	39.02	9.243	9.703	0.806	0.92	0.9<br>53	0.09892<br>979526	0.102757<br>0063	0.96
26	6	15	654	764	292604	I25-40-49<br>I42-40-49<br>I50-40-49	I21-40-49<br>I20-50-59<br>I21-50-59<br>I24-50-59	I42-50-59<br>I44-50-59<br>I50-50-59<br>I51-50-59<br>I42-60-69<br>I44-60-69<br>I44-70-79	5.057	7.379	31.115	59.641	9.089	12.743	0.685	0.522	0.7<br>13	0.02460<br>949692	0.021293<br>97016	1.16
27	6	139	451	6013	287434	N81-50-59	N80-50-59<br>N84-50-59<br>N85-50-59<br>N88-50-59<br>N95-50-59<br>N80-60-69	N81-60-69<br>N99-60-69	4.508	4.284	20.665	23.969	6.215	6.133	1.052	0.862	1.0<br>13	0.00032<br>5311724<br>3	0.000162<br>5487646	2.00
27	7	94	183	6538	226470	N81-50-59	N80-50-59<br>N84-50-59<br>N85-50-59<br>N88-50-59<br>N95-50-59<br>N80-60-69	N81-60-69<br>N99-60-69	6.749	5.364	42.59	33.923	10.847	7.876	1.258	1.255	1.3<br>77	0	0.027866<br>66857	0.00
28	5	1571	6213	0	331945	D12-40-49	I84-40-49<br>K64-50-59	D12-50-59<br>C18-60-69<br>C19-60-69<br>D12-60-69	NA	NA	NA	NA	NA	NA	NA	NA	NA	0.00029<br>8183936<br>4	0.000636<br>5372374	0.47
28	6	38	629	2750	290620	D12-40-49	I84-40-49<br>K64-50-59	D12-50-59<br>C18-60-69<br>C19-60-69<br>D12-60-69	4.308	5.666	20.44	28.875	7.323	8.235	0.76	0.708	0.8<br>89	0.00149<br>9250375	0.001434<br>72023	1.04

30	6	3194	4600	0	286243	D17-50-59	D48-50-59<br>D17-60-69<br>D48-60-69<br>D17-70-79	N84-60-69<br>N88-60-69	NA	NA	NA	NA	NA	NA	NA	NA	NA	0.00613<br>9917014	0.000626<br>1740764	9.81
30	7	182	5446	7633	220024	D17-50-59	D48-50-59<br>D17-60-69<br>D48-60-69<br>D17-70-79	N84-60-69<br>N88-60-69	8.099	5.242	45.367	28.376	9.778	7.525	1.545	1.599	1.2<br>99	0.01113<br>610784	0	Inf
31	3	127	315	2837	258334	M51-20-29	E11-20-29<br>E66-20-29<br>G47-20-29<br>E65-30-39<br>L02-30-39	G55-30-39<br>M43-30-39<br>M48-30-39<br>M51-30-39<br>G54-40-49<br>G55-40-49<br>G57-40-49<br>M40-40-49<br>M51-40-49<br>M54-40-49<br>M96-40-49<br>G54-50-59<br>M96-50-59<br>M96-60-69	3.467	3.18	22.508	19.25	4.921	4.105	1.09	1.169	1.1<br>99	0	0	NA
31	4	7	3904	738	289303	M51-20-29	E11-20-29<br>E66-20-29<br>G47-20-29<br>E65-30-39<br>L02-30-39	G55-30-39<br>M43-30-39<br>M48-30-39<br>M51-30-39<br>G54-40-49<br>G55-40-49<br>G57-40-49<br>M40-40-49<br>M51-40-49<br>M54-40-49<br>M96-40-49<br>G54-50-59<br>M96-50-59<br>M96-60-69	3.83	3.449	20.633	21.692	4.649	4.585	1.11	0.951	1.0<br>14	0	0.000220<br>6184518	0.00
32	5	10	50	3283	336386	M16-40-49	E66-40-49<br>I89-40-49<br>K42-40-49<br>K43-40-49<br>E65-50-59<br>K42-50-59<br>K43-50-59	M16-50-59<br>M87-50-59	4.48	4.25	28.76	30.951	8.32	5.875	1.054	0.929	1.4<br>16	0	0	NA
32	6	10	240	8275	285512	M16-40-49	E66-40-49<br>I89-40-49<br>K42-40-49<br>K43-40-49	M16-50-59<br>M87-50-59	6.037	5.042	38.625	34.802	8.817	7.195	1.197	1.11	1.2<br>25	0.00390<br>0074178	0	Inf

							E65-50-59<br>K42-50-59<br>K43-50-59													
34	3	127	315	2837	258334	M51-20-29	E11-20-29<br>E66-20-29<br>G47-20-29<br>E65-30-39<br>L02-30-39	G55-30-39<br>M43-30-39<br>M48-30-39<br>M51-30-39<br>G55-40-49<br>G57-40-49<br>M51-40-49<br>M96-40-49	3.467	3.18	22.508	19.25	4.921	4.105	1.09	1.169	1.1<br>99	0	0	NA
34	4	7	3904	738	289303	M51-20-29	E11-20-29<br>E66-20-29<br>G47-20-29<br>E65-30-39<br>L02-30-39	G55-30-39<br>M43-30-39<br>M48-30-39<br>M51-30-39<br>G55-40-49<br>G57-40-49<br>M51-40-49<br>M96-40-49	3.83	3.449	20.633	21.692	4.649	4.585	1.11	0.951	1.0<br>14	0	0.000220<br>6184518	0.00
35	6	380	12579	120	280958	M96-50-59<br>M96-60-69	M51-50-59<br>A69-60-69<br>G54-60-69<br>G57-60-69<br>G57-70-79	G55-60-69<br>G95-60-69<br>M48-60-69<br>M50-60-69<br>M51-60-69<br>G54-70-79<br>G55-70-79<br>G95-70-79<br>M47-70-79<br>M48-70-79<br>M50-70-79<br>M51-70-79<br>M54-70-79<br>M70-70-79<br>M93-70-79<br>M96-70-79	5.748	7.333	37.359	54.067	8.693	10.458	0.784	0.691	0.8<br>31	0.00199<br>8589563	0.002	1.00
35	7	0	29	352	232903	M96-50-59<br>M96-60-69	M51-50-59<br>A69-60-69<br>G54-60-69<br>G57-60-69<br>G57-70-79	G55-60-69<br>G95-60-69<br>M48-60-69<br>M50-60-69<br>M51-60-69<br>G54-70-79<br>G55-70-79<br>G95-70-79<br>M47-70-79<br>M48-70-79<br>M50-70-79<br>M51-70-79<br>M54-70-79	6.586	8.065	40.483	57.426	10.586	12.716	0.817	0.705	0.8<br>32	0.00218<br>7571886	0.002127<br>753604	1.03

								M70-70-79<br>M93-70-79<br>M96-70-79											
35	8	1	584	41	218998	M96-50-59<br>M96-60-69	M51-50-59<br>A69-60-69<br>G54-60-69<br>G57-60-69<br>G57-70-79	G55-60-69<br>G95-60-69<br>M48-60-69<br>M50-60-69<br>M51-60-69<br>G54-70-79<br>G55-70-79<br>G95-70-79<br>M47-70-79<br>M48-70-79<br>M50-70-79<br>M51-70-79<br>M54-70-79<br>M70-70-79<br>M93-70-79<br>M96-70-79	8.691	8.439	82.165	67.195	16.407	15.902	1.03	1.223	1.0<br>32	0.01025<br>641026	0.006390<br>357284	1.60
